# BODIPY-tagged β-lactams as selective quenched activity-based probes to target human neutrophil elastase

**DOI:** 10.1039/d6cb00206d

**Published:** 2026-10-29

**Authors:** Rita Felix, Luís Carvalho, Ismael Rufino, Rita Guedes, Ana Margarida Madureira, Ana Mallo-Abreu, Lídia Gonçalves, Olga Genilloud, Rosario Fernandez-Godino, Maria C. Ramos, Rui Moreira

**Affiliations:** a Research Institute for Medicines (iMed.ULisboa), Faculty of Pharmacy, Universidade de Lisboa Lisboa Portugal ritafelix@ff.ulisboa.pt; b Faculty of Pharmacy, Universidade de Lisboa Lisboa Portugal; c The Francis Crick Institute 1 Midland Road London NW1 1AT UK; d Centro Singular de Investigación en Química Biolóxica e Materiais Moleculares (CIQUS), Universidade de Santiago de Compostela 15782 Santiago de Compostela Spain; e Departamento de Química Orgánica, Facultade de Farmacia, Universidade de Santiago de Compostela 15782 Santiago de Compostela Spain; f Fundación MEDINA Av. Conocimiento 34, Health Sciences Technology Park 18016 Granada Spain

## Abstract

Activity-based probes are indispensable tools for interrogating protease function, and quenched fluorescent variants enable dynamic, real-time imaging of enzymatic activity. Despite these advances, very few quenched activity-based probes (qABPs) have been reported for serine proteases, which constitute the largest and most diverse mechanistic class of proteases. β-Lactams have been extensively used to develop molecular tools and drugs designed to bind or be hydrolysed by serine-dependent bacterial enzymes. Here, we report the first monocyclic β-lactam-containing qABPs for detecting serine proteases. Both the enzyme-triggered activation mechanism and intrinsic reactivity of the probes were highly dependent on the relative position of the BODIPY-FL fluorophore and quencher moiety at the β-lactam core. qABPs displaying the most efficient turn-on mechanism were shown to selectively target human neutrophil elastase (HNE) in different human cell lysates. The most successful qABP was rapidly internalised and targeted HNE in U937 cells and human neutrophils. These results demonstrate the potential of the modular β-lactam warhead to develop turn-on probes to track neutrophil serine proteases in live cells.

## Abstract 2

BODIPY-tagged quenched activity-based probes enable selective detection of active human neutrophil elastase with low background fluorescence and enhanced imaging performance.

## Graphical abstract

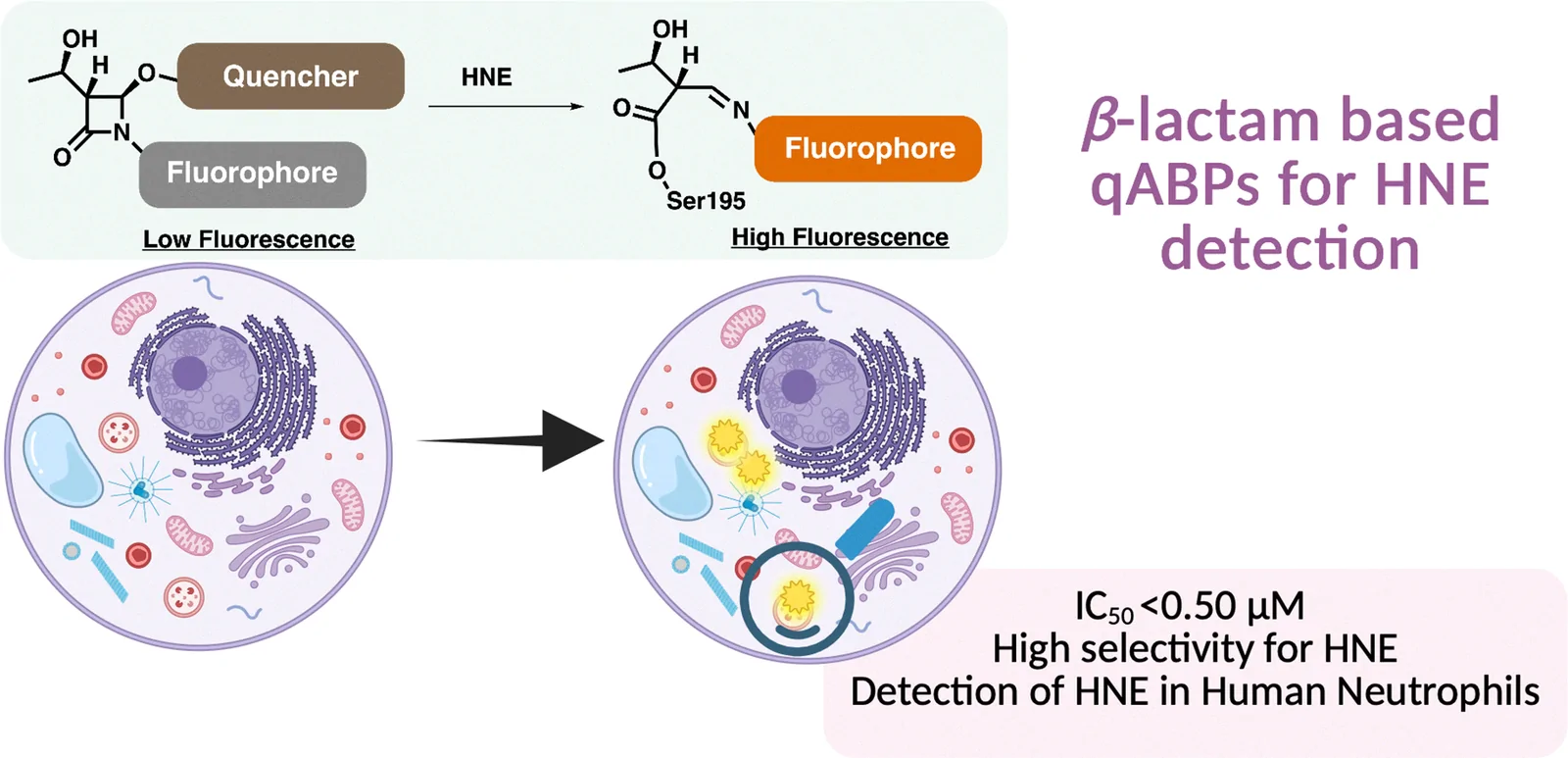

## 1. Introduction

Serine hydrolases encompass a large superfamily of enzymes that play key functions in biological processes in mammals, bacteria and viruses. Despite their importance, the role of many serine hydrolases in the development and progress of several diseases remains poorly characterised.^1–3^ Human neutrophil elastase (HNE) is a serine hydrolase of the chymotrypsin family expressed in polymorphonuclear neutrophils and has been associated with lung related diseases, such as cystic fibrosis, chronic obstructive pulmonary disease and acute respiratory distress syndrome.^4,5^ It has also been recently reported that HNE is present in the tumour microenvironment, promoting tumour growth and metastasis.^6–8^ However, the impact of HNE released to the tumour microenvironment on cancer progression remains largely unexplored due to the lack of selective chemical tools to study enzyme localisation and dynamics in cells and tissues.

Activity-based probes (ABPs) are small molecule tools used in chemical proteomics and cell imaging, incorporating an electrophilic group that covalently reacts with catalytic amino acid residues and a reporter tag for detection and enrichment of probe-labelled enzymes.^1,9^ The selectivity displayed by ABPs is strongly dependent on the structural motifs required for optimal molecular recognition by the target enzyme and the intrinsic reactivity of the warhead.^9^ ABPs containing a fluorophore tag enable both sensitive gel-based visualisation and fluorescence imaging of enzymatic activity, thus being extensively used as reagents for the detection and study of enzymes in cells. However, background signal generated by intrinsic fluorescence of the probe prevents successful application in real-time imaging with ABPs. Achieving significant signal-to-background ratio typically requires extensive washing steps *in vitro* or clearing times *in vivo* to reduce the background signal. A solution for this problem is offered by quenched ABPs (qABPs), in which a fluorescence quencher is incorporated as a leaving group. A requirement for this approach is the presence of a warhead with a single leaving group connected to a fluorescence quencher. A key advantage of qABPs over traditional ABPs is their fluorescence activation exclusively upon target enzyme binding, eliminating background signal from unreacted probe in solution and thereby overcoming major imaging limitations (Fig. 1).^10–13^

**Fig. 1:**
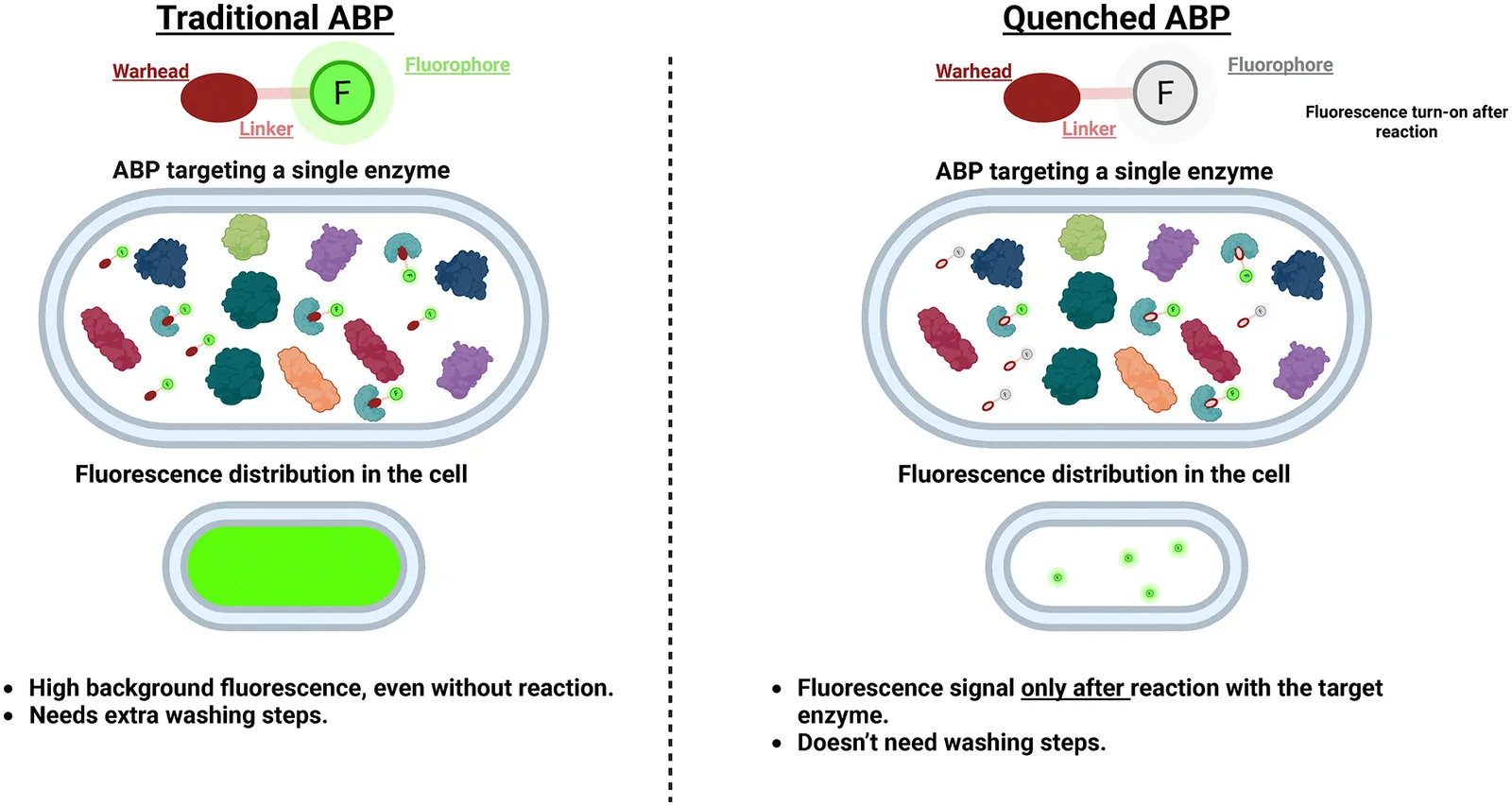
Activity-based probes (ABPs) *vs.* quenched activity-based probes (qABPs) in biological applications. Created in BioRender. Moreira, R. (2026) https://BioRender.com/x6jfe5m.

While qABPs for cysteine proteases are well established experimental tools, their counterparts designed to target serine hydrolases, including HNE, remain largely underexplored.^14–23^ The first near-infrared fluorogenic off–on probe reported for HNE, incorporating a hemicyanine fluorophore, was shown to restore fluorescence upon reaction with HNE (Fig. 2A).^24^ However, because the fluorophore is released into solution, this strategy is not well suited to gel-based assays or pull-down experiments. Alternatively, ABPs can incorporate a linker between the reactive group and the tag, which functions not only as a spacer but also as a key determinant of selectivity. A common approach in cysteine-targeted ABPP involves introducing a short amino-acid sequence to direct a broadly reactive chemotype toward a specific protein. Using this principle, Rios *et al.*^25^ developed “triple qABPs” containing three fluorescein moieties paired with three DABSYL quenchers *via* a peptide sequence, which were selectively cleaved by HNE between isoleucine and norleucine (Fig. 2B). While multiple fluorophore/quencher pairs is a well-established strategy to reduce background and amplify signal, it substantially increases the overall molecular weight. More recently, cell-permeable qABPs based on the broadly reactive phosphonate ester warhead enabled fine-tuning of selectivity across different protein targets by varying the amino-acid sequence between the fluorophore and the linker (Fig. 2C and D), underscoring the critical role of linker design in qABP performance.^26,27^ Nevertheless, incorporating peptide-based linkers increases both structural complexity and synthetic demands.

**Fig. 2:**
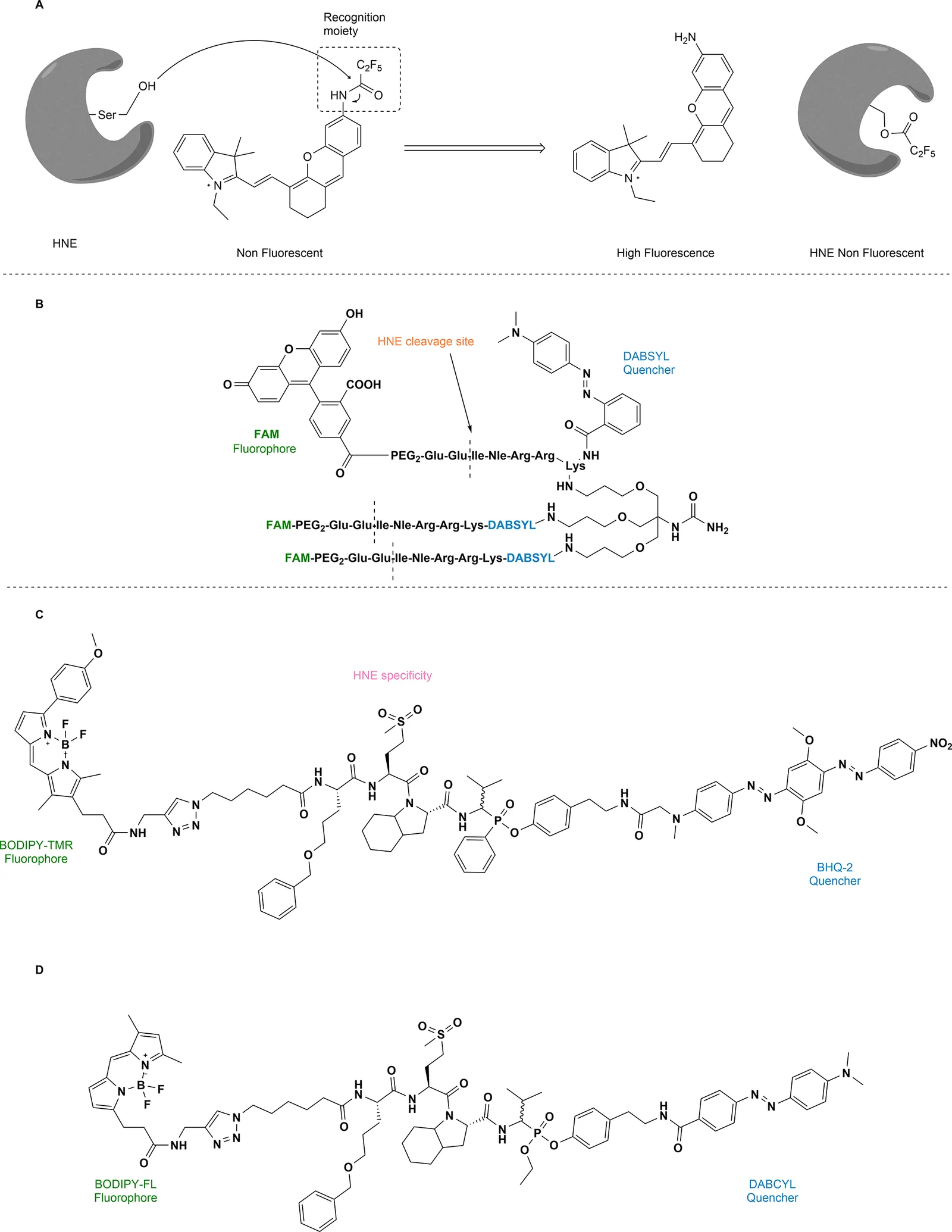
Examples of qABPs that target HNE. (A) qABP where selective activation by HNE releases the amino xanthene fluorophore. (B) Peptide-based “triple qABP”, comprising three fluorophore/quencher pairs connected through a peptide sequence selectively cleaved by HNE. (C/D). Phosphonate ester qABPs, where the selectivity is determined by the amino acid sequence in the linker.

Monobactams are a class of narrow-spectrum β-lactam antibiotics with a unique monocyclic ring structure, that exert their activity by reacting with a conserved active-site serine residue in penicillin-binding proteins. While being used primarily to tackle infections caused by aerobic Gram-negative bacteria, monobactam moieties have also been employed to inhibit human serine hydrolases, including HNE^28–31^ and dipeptidyl peptidases 8 and 9.^32^ Herein, we report the first qABPs featuring a monocyclic β-lactam warhead and endowed with a novel turn-on mechanism. These probes demonstrated strong inhibition and high selectivity toward HNE, enabling its detection in complex proteomes.

## 2. Results and discussion

### 2.1. Design and chemistry

The design of the monobactam qABPs relies on a mechanism-based enzyme inhibition to enable the formation of a stable acyl–enzyme complex. The probes were designed based on reported mechanism-based β-lactam inhibitors of HNE. The β-lactam ring was selected as the reactive moiety responsible for acylation of the catalytic serine residue, whereas the phenol group was introduced to enable the fragmentation process after enzyme acylation. This strategy follows earlier studies showing that β-lactams containing a phenol group at C-4, or another leaving group at the 1′ position, can function as mechanism-based inhibitors.^33,34^ While the high selectivity towards HNE was only confirmed experimentally, the scaffold was chosen on the basis of the favourable results previously obtained with related β-lactam HNE inhibitors.^4,29^ Two types of qABPs are reported, differing in the positions where fluorophore and quencher are installed, as well as on their activation and quencher-releasing mechanism (Fig. 3). While type I probes contain the quencher as part of a *N*-1 acyloxymethyl moiety, their type II counterparts present the quencher at C-4 of the 4-membered ring. In both cases, β-lactam ring-opening upon reaction with a catalytic serine residue (Ser195) can promote a 1,2-elimination to release the quencher and form an electrophile that can react with a second amino acid residue close to the active site, resulting in an irreversible inhibition mechanism.

**Fig. 3:**
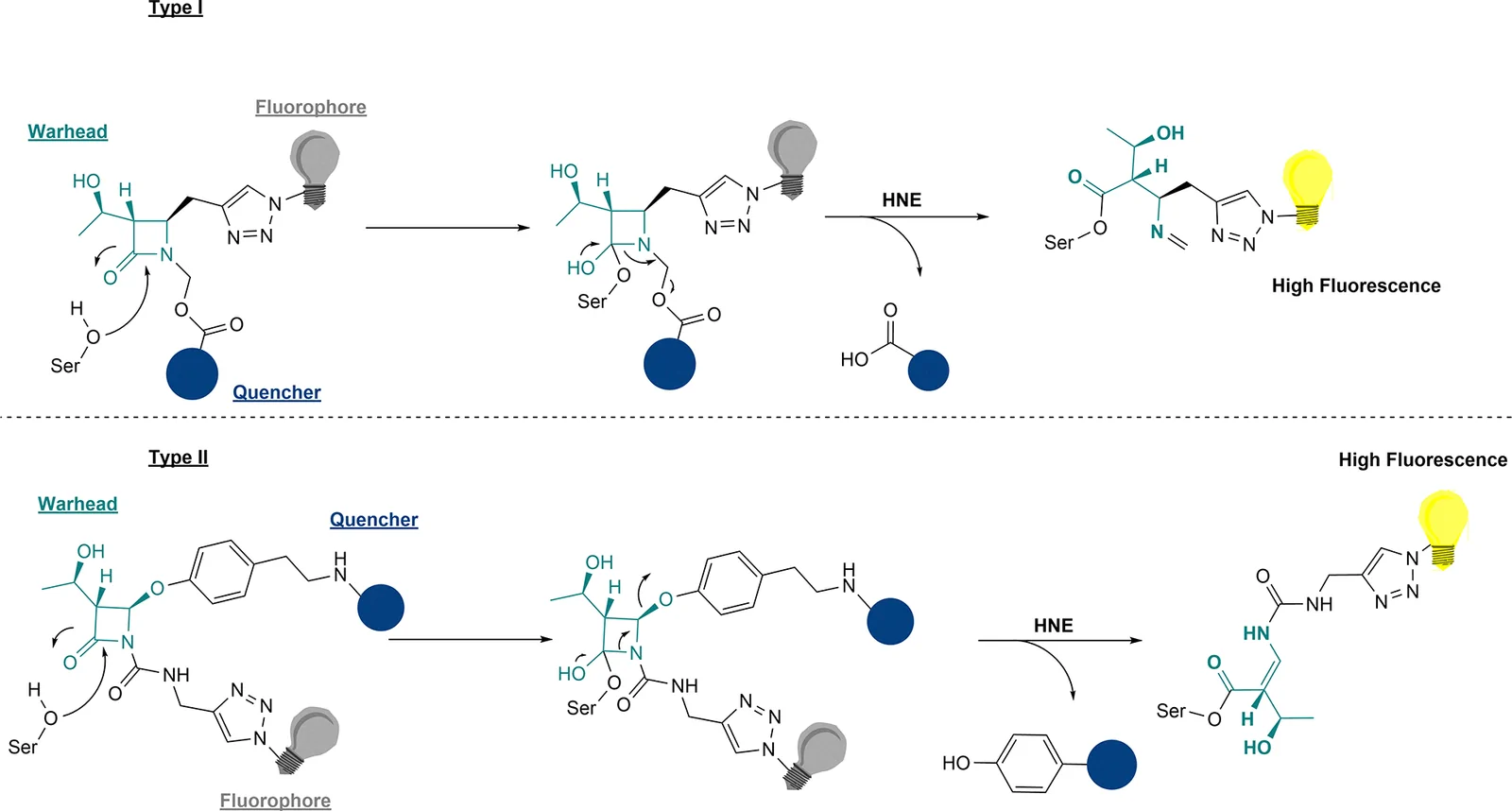
General qABP activation mechanism.

The qABPs incorporate the fluorophore BODIPY-FL and either a dinitrobenzene moiety or cAB40 as quenchers. For type I probes (Fig. 4), synthesis began from the commercially available azetidine-2-one 1. A Barbier-type reaction with propargyl bromide and zinc afforded the alkyne intermediate 2, with retention of stereochemistry at the chiral centre, as confirmed by ^1^H NMR.^35^ Subsequent hydroxymethylation at N-1 and coupling of the quencher, yielded compounds 7i-iii in good yields. Deprotection of the hydroxyethyl group using tetrabutylammonium fluoride, followed by the copper(i)-catalysed azide–alkyne cycloaddition with the previously synthesised BODIPY-FL azide 9, furnished to the final type I qABPs 10–12.

**Fig. 4:**
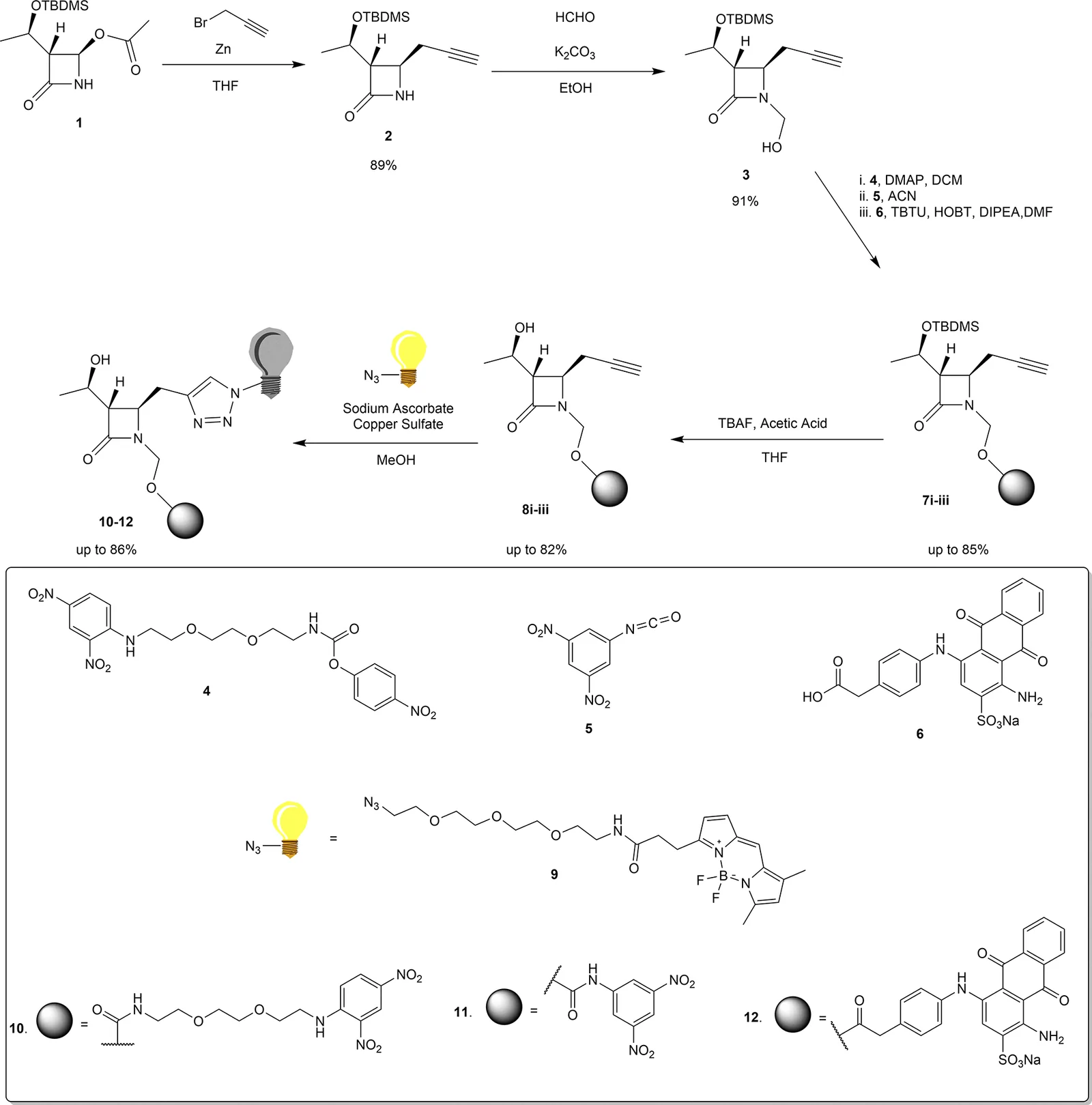
Synthetic strategy used to obtain type I qABPs. Compound 1 reacts with propargyl bromide through a Barbier-type reaction to achieve the enantiopure lactam 2. Followed by a hydromethylation of the lactam ring to obtain compound 3. The quencher moiety previously synthesised (4–6) was coupled with 3 generating intermediates 7i-iii that was further deprotected (8i-iii) and finally a click reaction was performed to achieve the final qABPs (10–12).

The synthesis of type II probes began with azetidin-2-one 1, followed by substitution of the acetoxy group with a phenoxy moiety bearing the quencher (Fig. 5). Reaction with carbamate 17 afforded the alkyne intermediates 18i-iii, which were subsequently deprotected. Final coupling with the BODIPY-FL azide *via* a copper(i)-catalysed click reaction yielded the type II probes 20–22.

**Fig. 5:**
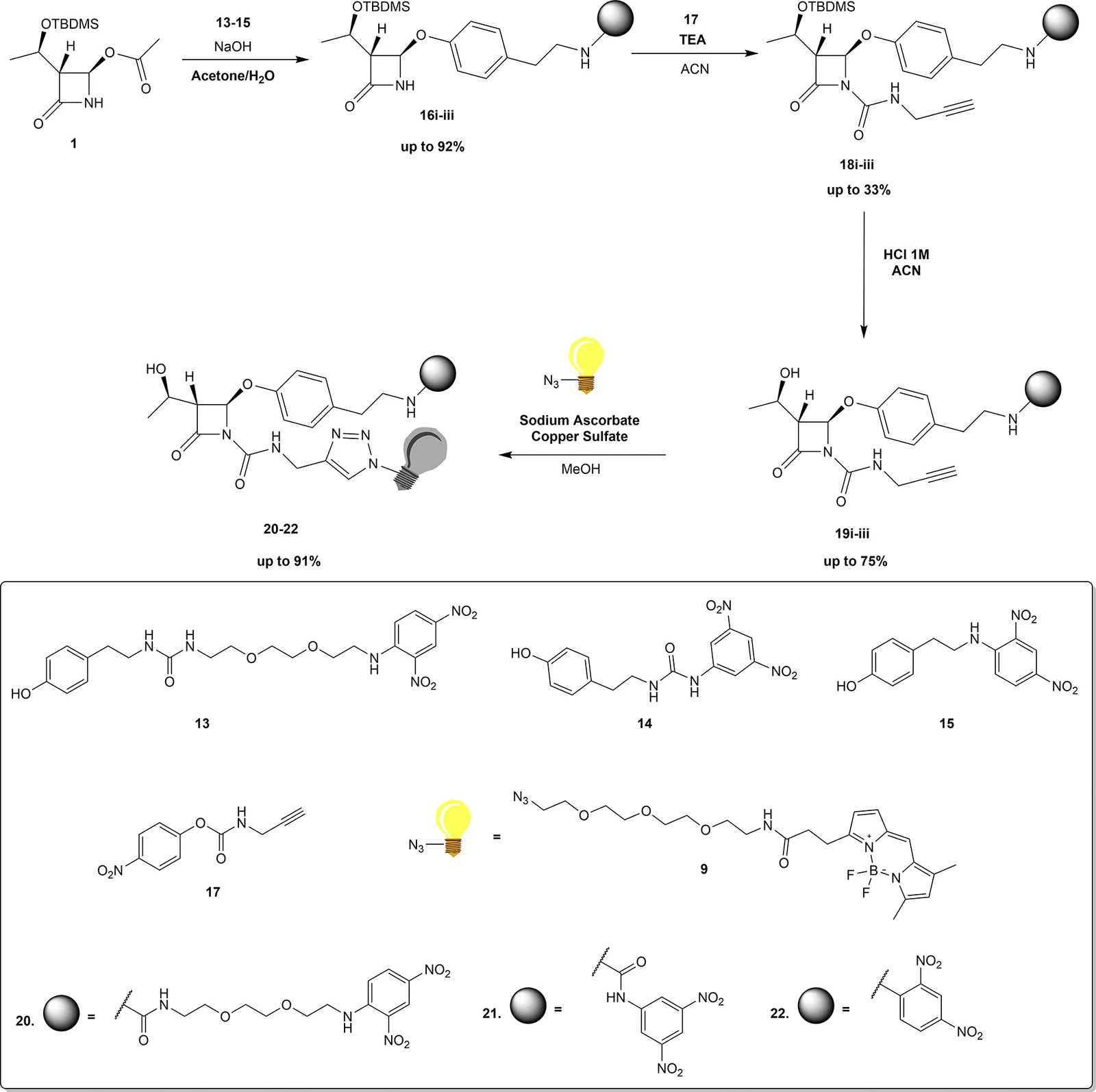
Synthetic strategy used to obtain type II qABPs. Compound 1 reacts with a phenol intermediate previously synthesised (13–15) that contains the quencher to obtain compounds 16i-iii. Followed by a urea formation on the lactam ring to obtain compounds 18i-iii. Compounds were deprotected (19i-iii) and finally a click reaction was performed to achieve the final qABPs (20–22).

#### 2.1.1. Photophysical properties and enzymatic activation

The photochemical properties were evaluated, and the stability of the probes was analysed in different pH values. All qABPs were stable in PBS (pH 7.4) up to 48 hours. The alkaline hydrolysis of lactams was used as simple guide to determine the usefulness of the probes as enzyme acylating agents. To simulate the attack of the catalytic serine residue, the stability of the probes was assessed by UV-vis spectrophotometry in alkaline solutions. Inspection of the spectra revealed that type I probes, 10 and 11, were hydrolysed only at pH 12 (Fig. S20 and S22), while 12 was hydrolysed at pH 10 (Fig. S24). In contrast, their type II counterparts displayed a shift in the UV spectra at pH 10, consistent with β-lactam ring-opening and release of the quencher moiety (Fig. S26, S28 and 30). To validate β-lactam ring opening and the consequent release of the quencher (turn-on of the probe) the reaction leading to quencher release was monitored by fluorescence scan. For type I qABPs, the fluorescence increase persisted for up to 12 hours. In contrast, type II qABPs reacted more rapidly, achieving a comparable increase in fluorescence within 2 hours. This difference indicates that, in type II probes, quencher release is faster and more efficient, an essential feature for the intended application.

To evaluate enzyme activation, porcine pancreatic elastase (PPE) was used, given its high homology with HNE. The compounds were incubated with PPE, and fluorescence was measured over time. With type II qABPs, fluorescence increased up to sixfold within 3 hours; however, with type I qABPs, only a small intensification was observed after 5 hours (Fig. S43). These results showed that, in the case of type II, the reaction to release the extinguishing fraction was significantly faster compared to type I (Fig. S44).

The fluorescence quantum yields (*Φ*_F_) were calculated for all the compounds (Table 1) using an indirect method.^36^ A solution of fluorescein in 0.1 M NaOH (*Φ*_F_ = 89%) was used as a reference. While the azide used in probe synthesis had a fluorescence quantum yield of 100%, most probes synthesised in this work had a value lower than 11%, suggesting efficient quenching of the probe fluorescence. Probes 11 and 21 were the only exceptions, with a value higher than 20%, which could suggest that the amide bond to the dinitrobenzene moiety decreased quencher efficacy. After activation the fluorescence quantum yield increased up to 92%, validating our proposed turn-on mechanism and denoting that the compounds can be used as qABPs to detect the targets with low background fluorescence (Table 1).

**Table 1:** Fluorescence quantum yields before (PBS) and after activation (NaOH)

	qABP	*Φ*_F_ (PBS), %	*Φ*_F_ (after activation), %
Type I	10	4	54
	11	26	86
	12	9	92
Type II	20	11	79
	21	21	77
	22	6	80

After the validation with PPE, we additionally validated probe 22 using pure HNE and human neutrophil lysate. These were incubated with the probe and the generation of fluorescence was measured through time (Fig. 6).

**Fig. 6:**
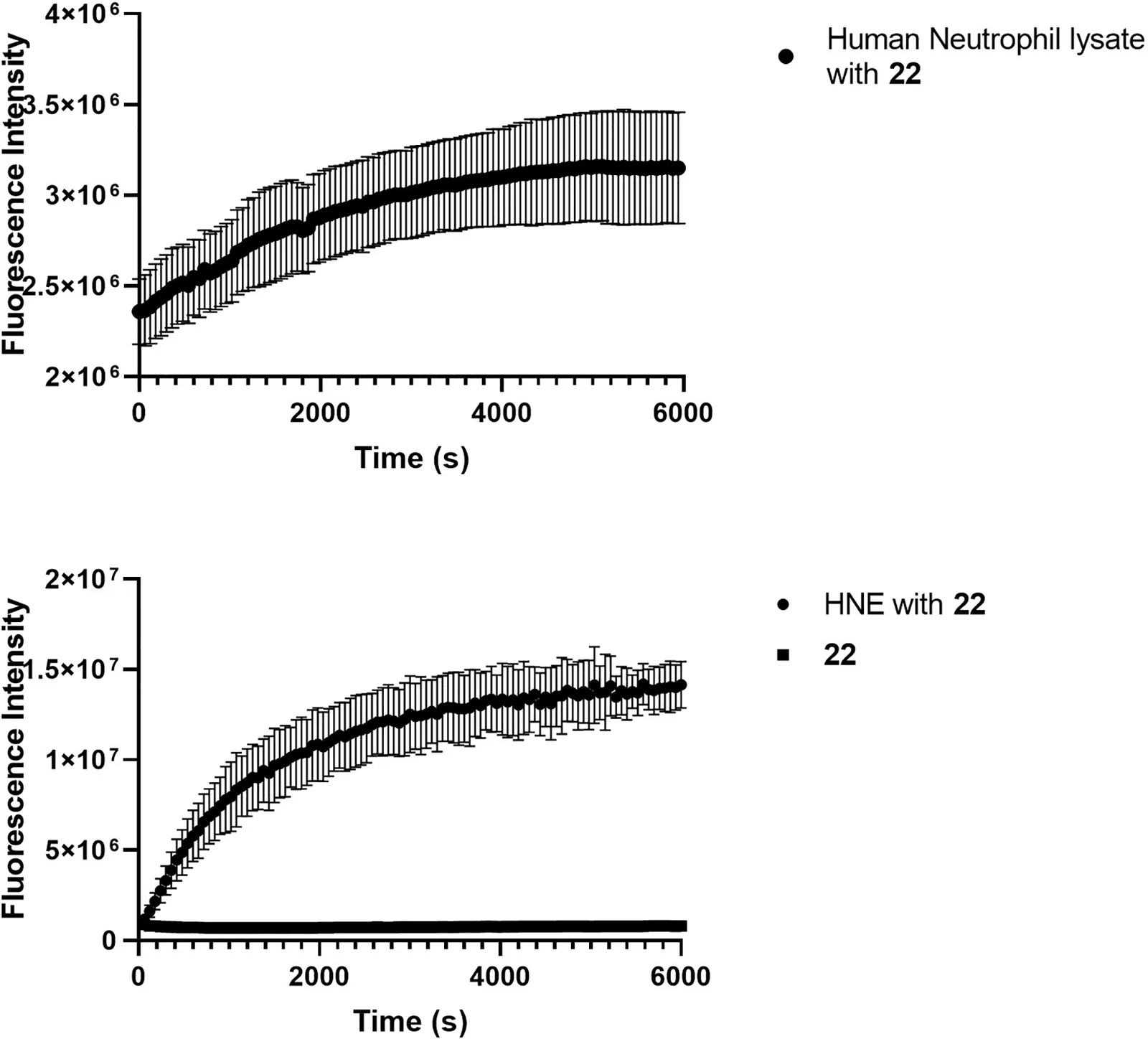
(A) Incubation of human neutrophils lysate (1 mg ml^−1^) with 22 (1 µM). (B) Human neutrophil elastase with 22 (1 µM)and 22 (1 µM) in assay buffer. Fluorescence was measured every minute for 2 hours, using as excitation wavelength 485 nm and emission wavelength 535 nm. Assays were performed in triplicate.

In both cases, an increase in the fluorescence signal was observed (more significant in the case of pure HNE), confirming the reaction of the probe with the target and sequential release of the quencher moiety. The fluorescence of the probe in the buffer assay was also tested and remained very low and stable during the assay. Complementarily, a jump-dilution assay was performed to determine whether the qABPs act as irreversible or reversible inhibitors. The assay showed that, after pre-incubation with the qABPs, the enzyme was no longer able to degrade its substrate, indicating that the inhibition induced by the qABPs is irreversible. (Fig. S45)

#### 2.1.2. Target selectivity

Enzymatic assays were performed against HNE and other serine proteases including chymotrypsin, thrombin, kallikrein and urokinase. These assays was based on competitive inhibition, once that at first the qABPs were incubated with the enzyme and after a fluorogenic substrate was added. If the enzyme was inactivated by the qABP there were no changes in the fluorescence signal, however, if there was some active enzyme it would cleave the fluorogenic substrate and there would be detected changes in the fluorescence signal All probes displayed strong inhibitory activity against HNE, with IC_50_ values below 0.5 µM. Type I qABPs 10 and 12 exhibited IC_50_ values of 0.50 and 0.12 µM, respectively. However, due to their slower quencher release and delayed fluorescence response compared to type II probes, which limits their practical application, subsequent biological evaluations focused exclusively on type II qABPs. In contrast, type II qABPs demonstrated high selectivity for HNE over related serine proteases, with IC_50_ values exceeding 10 µM across the tested panel (Table 2). This selectivity is particularly noteworthy given the close similarity between PPE and HNE, which share approximately 43% sequence homology and highly similar active sites; consequently, PPE is often used in preliminary assays for HNE inhibitors.^37^

**Table 2:** Inhibitory potency of probes 20–22 against different serine proteases

IC_50_ (µM)			
	20	21	22
Chymotrypsin	>10	>10	>10
Thrombin	>10	>10	>10
Kallikrein	>10	>10	>10
Urokinase	>10	>10	>10
PPE	>10	>10	>10
HNE	0.18	0.13	0.19

To further assess the selectivity of qABPs toward HNE in more complex environments, SDS-PAGE experiments were performed using both pure enzyme and HNE spiked into different proteomes. When incubated with purified HNE and analysed by gel electrophoresis, fluorescence was observed exclusively for type II probes (Fig. 7). Notably, pre-incubation of the enzyme with ONO-6818, a potent and selective HNE inhibitor,^38^ abolished the fluorescence signal (Fig. 7), indicating that the probes compete for binding at the HNE active site.

**Fig. 7:**
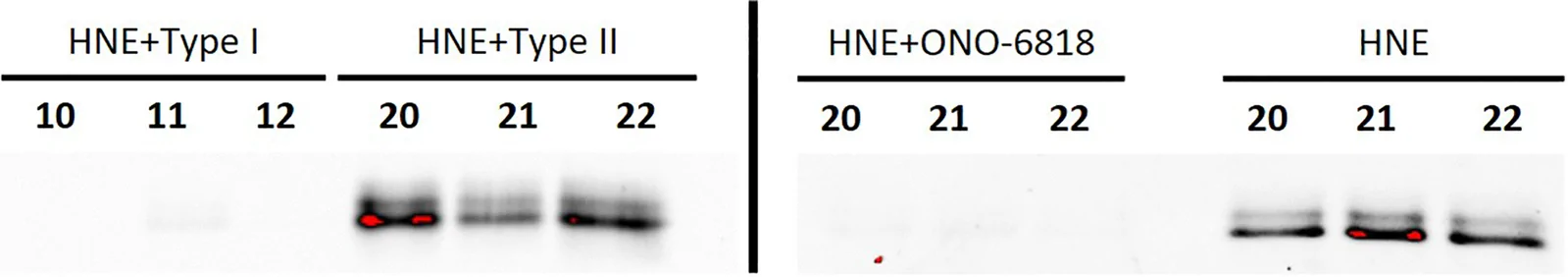
Incubation of the probes (1 µM) with HNE (left); pre-incubation with ONO-6818 (1 µM) (right).

After confirming HNE engagement by gel assays, human embryonic kidney cell (HEK293) and epidermal carcinoma cell (A431) lysates were spiked with HNE and incubated with type II probes. The probes selectively detected HNE in the HEK293 proteome (Fig. 8A). Probe 22 was also tested with decreasing concentrations of HNE, with minimal increase in background labelling and remaining selective for HNE (Fig. 8B). Similarly, the A431 cell lysate experiment showed that all probes of type II could selectively detect HNE (Fig. 8C), with probe 22 selectively detecting HNE. (Fig. 8D). To evaluate the sensitivity of probe 22 the fluorescence intensity was analysed in both cases, showing a linear response with the increase of HNE concentration over the tested range (*R*^2^ = 0.971 for HEK293 and *R*^2^ = 0.948 for A431 lysates). The limit of detection (LOD), calculated from the slope of the calibration curve and the standard deviation of the blank samples, was estimated to be 30.7 nM in HEK293 lysates and 7.4 nM in A431 lysates. These results indicate that probe 22 is able to detect low nanomolar concentrations of HNE in biologically relevant matrices (Fig. S48 an S49).

**Fig. 8:**
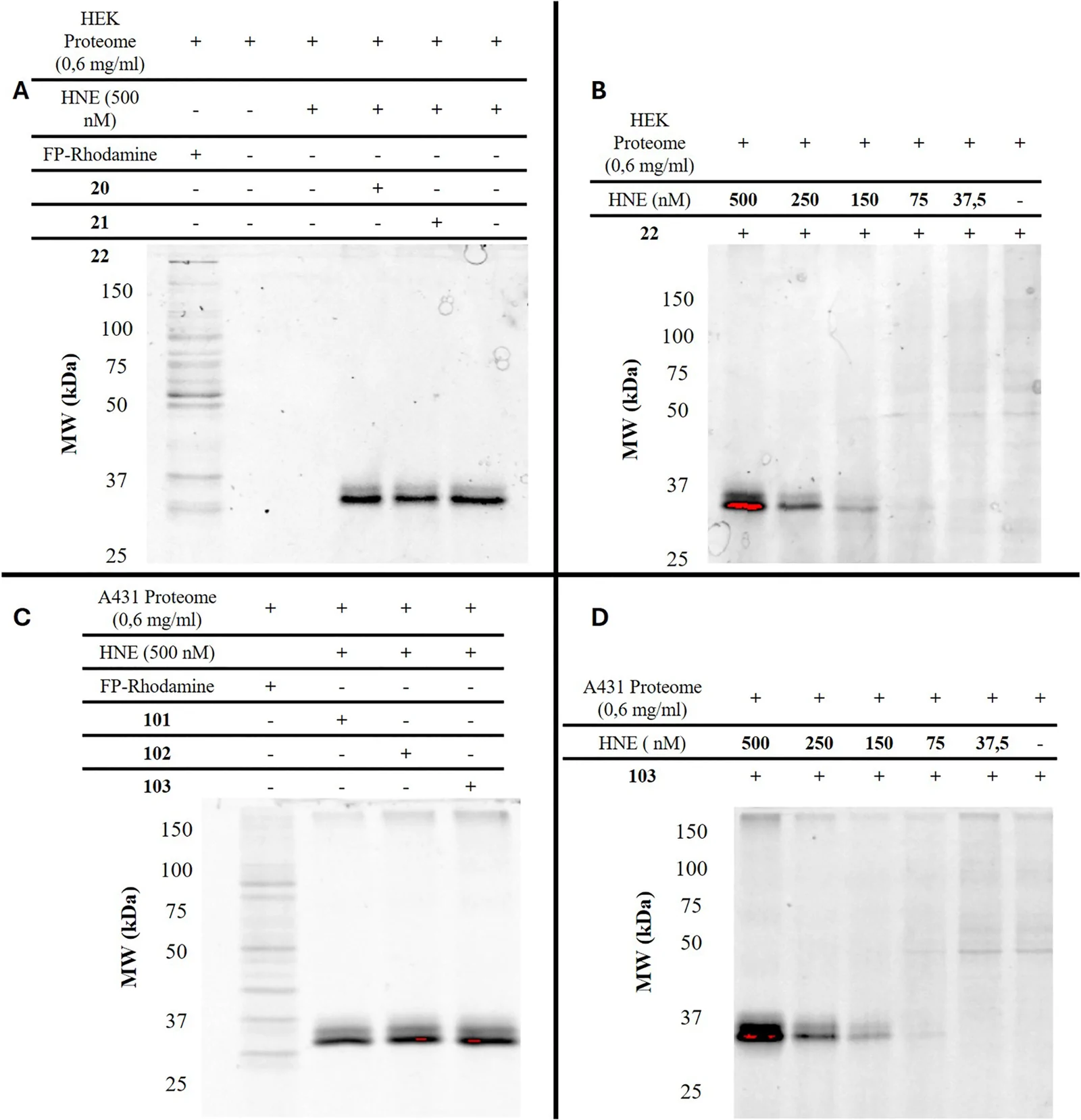
(A) Type II probes incubated with HEK293 lysate spiked with HNE (500 nM); (B) 22 (1 µM) incubated with HEK293 lysate spiked with decreasing concentrations of HNE; (C) type II probes incubated with A431 lysate spiked with HNE (500 nM); (D) 22 (1 µM) incubated with A431 lysate spiked with decreasing concentrations of HNE.

Once the potential of the probes to target HNE was validated in a complex proteome, 22 was incubated with human neutrophils and U937 cell lysates (U937 is a monocytic cell line that natively expresses HNE^32^). As presented in Fig. 9, in both cases probe 22 was able to identify endogenous HNE in a complex proteome without requiring overexpression.

**Fig. 9:**
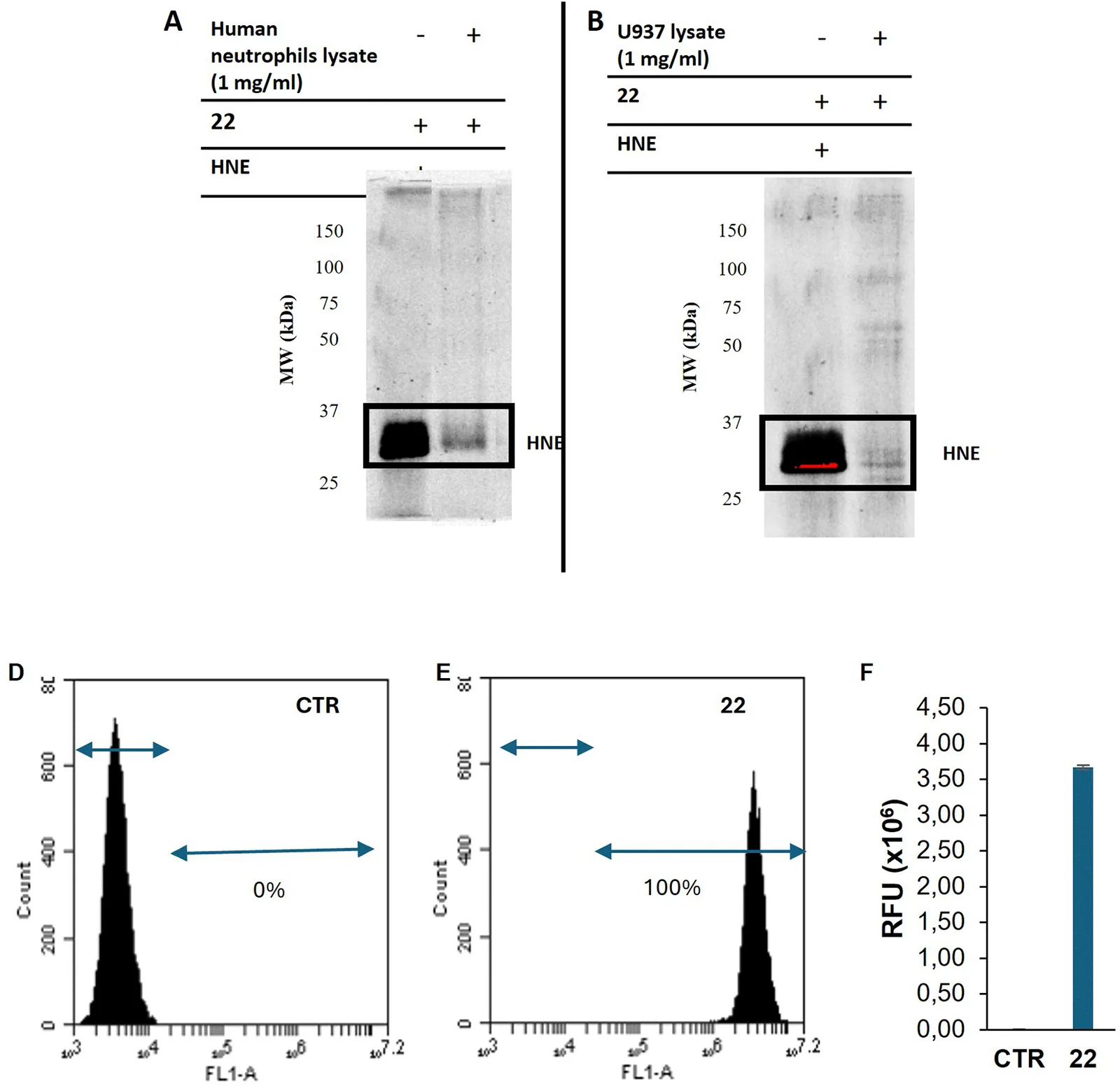
(A) Probe 22 (1 µM) incubated with a lysate of human neutrophils. (B) Probe 22 (1 µM) incubated with U937 cell lysate. (D) Flow cytometry of living U937 cells. (E) Flow cytometry of living U937 cells treated with 22 (1 µM) for 2 h; in both cases the *x*-axis represents the fluorescence intensity measure, and the cell count is showed in the *y*-axis. (F) Bars graph (mean ± SD) represents the median fluorescence intensity of the control and the cells treated with 22.

Encouraged by these promising results, we next evaluated whether the synthesised probes could be internalised by living cells. Cellular uptake was assessed in U937 cells and human neutrophils using flow cytometry and fluorescence imaging, respectively. In U937 cells, flow cytometry analysis showed that control cells (untreated with 22) exhibited no detectable fluorescence, whereas cells treated with 22 displayed a marked increase in fluorescence intensity, indicating efficient probe internalization and activation (Fig. 9).

For human neutrophils, internalisation was assessed by fluorescence imaging using a high-content analysis system. A strong fluorescence signal was observed in the cytoplasm following incubation with 22, whereas the negative control showed no detectable fluorescence (Fig. 10), further confirming efficient cellular uptake.

**Fig. 10:**
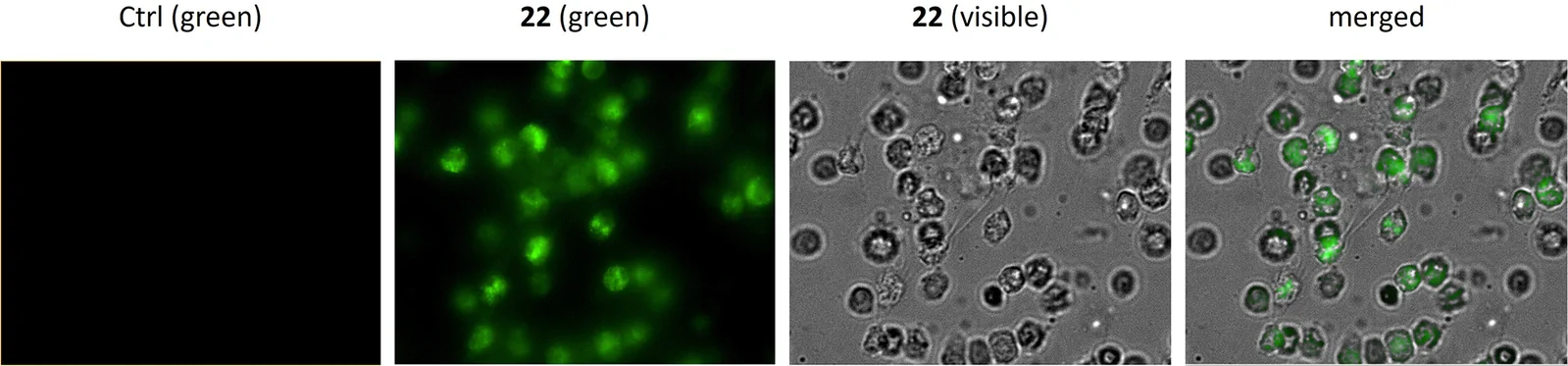
Incubation of 22 with human neutrophils. Images were taken after 2 h of 22 additions.

Overall, the BODIPY-tagged β-lactam probes presented remarkable selectivity and strong potential as qABPs for detection of HNE in complex proteomes, highlighting their value as tools to elucidate the role of HNE in the tumour microenvironment. To further investigate the structural features underlying this high selectivity, both covalent and non-covalent docking studies were performed using compound 22.

Molecular docking of probe 22 showed that the hydroxyethyl substituent at C-3 of the β-lactam warhead occupies the primary binding S1 pocket, with the hydroxyl group forming a hydrogen bond with Val216 (Fig. 11A). This observation suggests that the predominantly hydrophobic S1 pocket can also accommodate polar interactions.

**Fig. 11:**
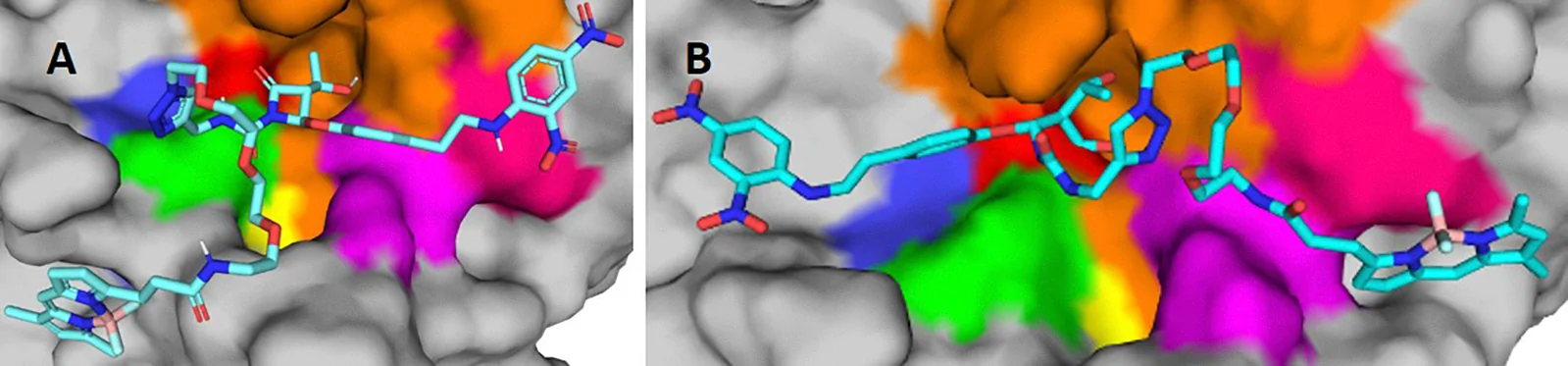
(A) Non-covalent docking pose of 22 in the active site of HNE, obtained from PDB 9SI4. Active site is highlighted with different colours, Ser-195 in red, His-57 in green and Asp-102 in yellow. (B) Covalent docking pose of 22 in the active site of HNE, obtained from PDB 9SI4. qABP 22 is presented as blue sticks and PDB 9SI4 is presented as a grey surface. Active site is highlighted with different colours, Ser-195 in red, His-57 in green and Asp-102 in yellow. Pictures were prepared using Pymol. (S1 pocket represented in orange, S2 pocket represented in pink, S1′ represented in blue, S3 represented in hotpink).

Regarding covalent docking (Fig. 11B), the results indicate that probe 22 establishes multiple interactions within the S1 pocket of HNE, primarily involving residues Phe41, Phe192, Gly193, Ser214, Phe215, Val216, and Arg217. Additional interactions were also observed at the enzyme surface, mainly with Leu35, Leu167, Ser214 (S1) and Val216 (S3) (Fig. S46). These findings support the capacity of the S1 pocket of HNE to accommodate polar substituents.

Overall, the synthesised probes presented remarkable selectivity and a strong potential to be used as qABPs to detect HNE in complex proteomes and could become an important tool to reveal the roles of HNE in the tumour microenvironment.

## 3. Conclusions

In this work, we describe a synthetic strategy to generate qABPs capable of selectively targeting HNE in biological media and complex proteomes without the need for a peptidyl linker for molecular recognition. The developed qABPs exhibited high quenching efficiency, with *Φ*_F_ values below 20%, which increased to up to 92% upon activation. Probe activation was confirmed through fluorescence assays using both purified HNE and human neutrophil lysates.

The probes displayed remarkable selectivity for HNE, even when compared to closely related serine hydrolases. Their effective detection and activation in biological matrices were further demonstrated across three different cell lines, with and without HNE spiking, including human neutrophil lysates.

Overall, this study provides a set of versatile chemical tools for investigating the role of HNE in the tumour microenvironment and supports its validation as a relevant target in cancer therapy.

## Author contributions

RM and RF conceived and designed the study. Synthesis was developed by RF and AM-A. Photophysical and biology data collection, and data analysis were carried out by RF, LC, AMM and LG. Flow cytometry and cell imaging studies were performed and analysed by MCR, RFG and OG. Computational studies were performed by IR and RG. All authors analysed the results and contributed to writing the manuscript.

## Conflicts of interest

There are no conflicts to declare.

## Supplementary Material

CB-OLF-D6CB00206D-s001

## Data Availability

The data supporting this article have been included as part of the supplementary information (SI). Supplementary information: includes detailed experimental procedures, synthetic methods, compound characterisation (NMR and HRMS spectra), and assay protocols. See DOI: https://doi.org/10.1039/d6cb00206d.
