# Supplementary material for: BODIPY-tagged β-lactams as selective quenched activity-based probes to target human neutrophil elastase
Source: RSC Chem Biol. 2026 Oct 29. Online ahead of print. doi: 10.1039/d6cb00206d (PMC13733405; doi:10.1039/d6cb00206d)
Supplement: Supplementary file 1 [file CB-OLF-D6CB00206D-s001.pdf]

Supplementary Information (SI) for RSC Chemical Biology.
This journal is © The Royal Society of Chemistry

Supporting Information

BODIPY-Tagged *β*-Lactams as Selective Quenched Activity-Based Probes to Target Human Neutrophil Elastase

Rita Felix[a,b]*, Luís Carvalho[c], Ismael Rufino[a,b], Rita Guedes[a,b], Ana Margarida Madureira[a,b], Ana Mallo-Abreu[d,e†], Lídia Gonçalves[a,b], Olga Genilloud[f], Rosario Fernandez-Godino[f], Maria C Ramos[f], Rui Moreira[a,b*]

[a] Research Institute for Medicines (iMed.ULisboa), Faculty of Pharmacy, Universidade de Lisboa. Lisboa, Portugal

[b] Faculty of Pharmacy, Universidade de Lisboa. Lisboa, Portugal

[c] The Francis Crick Institute, 1 Midland Road, London NW1 1AT, United Kingdom

[d] Centro Singular de Investigación en Química Biolóxica e Materiais Moleculares (CIQUS), Universidade de Santiago de Compostela, 15782 Santiago de Compostela, Spain

[e] Departamento de Química Orgánica, Facultade de Farmacia, Universidade de Santiago de Compostela, 15782 Santiago de Compostela, Spain

[†] Present address: Laboratory of Medicinal Chemistry, Faculty of Pharmacy and Food Sciences, University of Barcelona, Av. Joan XXIII, 27-31, E-08028 Barcelona, Spain, and Institute of Biomedicine of the University of Barcelona (IBUB), Av. Diagonal 643, E-08028 Barcelona, Spain

[f] Fundación MEDINA, Av. Conocimiento 34, Health Sciences Technology Park, 18016 Granada, Spain

Index

Materials and General Methods 3

Synthesis BODIPY-FL 4

Synthesis of qABPs 6

NMR and HRMS spectra of qABPs 21

Stability of qABPs in different pHs 29

Fluorescence of the qABPs before and after activation 35

Activation of qABPs with Pancreatic Porcine Elastase 42

Jump Dillution Assay 44

qABPs inhibitory ability against HNE 45

Docking results 46

UV-Stability 48

Gel-Based Assays 48

Fluorescence Studies 50

Fluorescence Scan 50

Fluorescence kinetics with HNE and neutrophils 50

Biological activity 51

Probe Internalisation by Flow Cytometry 52

Live Cell Imaging 52

Molecular docking calculations 52

Materials and General Methods

Generally, the chemicals and enzymes were purchased from Merck, Fluorochem, Alfa Aesar and TCI and used without further purification. Solvents were purchased from the same suppliers and were distilled or dried using conventional described methods.165 The purification of the mixtures to isolate the compounds when performed by flash chromatography was used SilicaFlash F60 silica gel (40–63 μm, 60 Å) for normal phase or LiChroprep® RP-18 (15-25 μm) for reverse-phase chromatography, some purifications were also performed in a CombiFlash RF-200 apparatus from Teledyne Isco with RediSep normal- or reverse-phase (C18) silica flash columns, or prepared columns with SilicaFlash F60 silica gel (40–63 μm, 60 Å) for normal phase or LiChroprep® RP-18 (15 25 μm) for reverse-phase. Chemical reactions were followed by thin layer chromatography (TLC) using Merck aluminium-backed sheets coated with 60 F254 silica gel or RP-18 F_{254}s and visualized in a UV lamp (λ_{max} = 254 nm or 330 nm). Characterisation of compounds (^{1}H, ^{13}C NMR) were performed at room temperature on a Bruker Biospin Fourier 300 MHz at the Faculty of Pharmacy, Universidade de Lisboa, Bruker 300 MHz Avance II, and Bruker 400 MHz Avance II instruments at Instituto Superior Técnico, Universidade de Lisboa. The purity of the compounds was evaluated through HPLC using HPLC Elite LaChrom (VWR Hitachi) system, composed by a L-2130 pump, a L-2300 column oven, a L-2400 UV detector, a LiChrospher® 100 RP-18 (5 μm) column and a Rheodyne® manual sample injector 20 μL loop. The chromatograms were analysed with EZChrom Elite software. Low resolution mass spectra analysis was performed using Electrospray Ionization technique, in a Micromass Quattro Micro API spectrometer (Micromass Quattromicro® API Spectrometer, with Watters® Alliance 2695 HPLC Module), to process the results was used MassLinkx software. HRMS were obtained through Direct injection on a Q Exactive™ Plus Hybrid Quadrupole-Orbitrap™ Mass Spectrometer using electrospray ionization (ESI) in positive mode. UV spectra were obtained in Thermo Scientific Evolution 201 UV-visible spectrophotometer and fluorescence spectra were measured with a Varian Cary Eclipse fluorimeter, in both cases the results were analysed in Origin Pro 9.0 and GraphPad Prism 8.

Synthesis BODIPY-FL

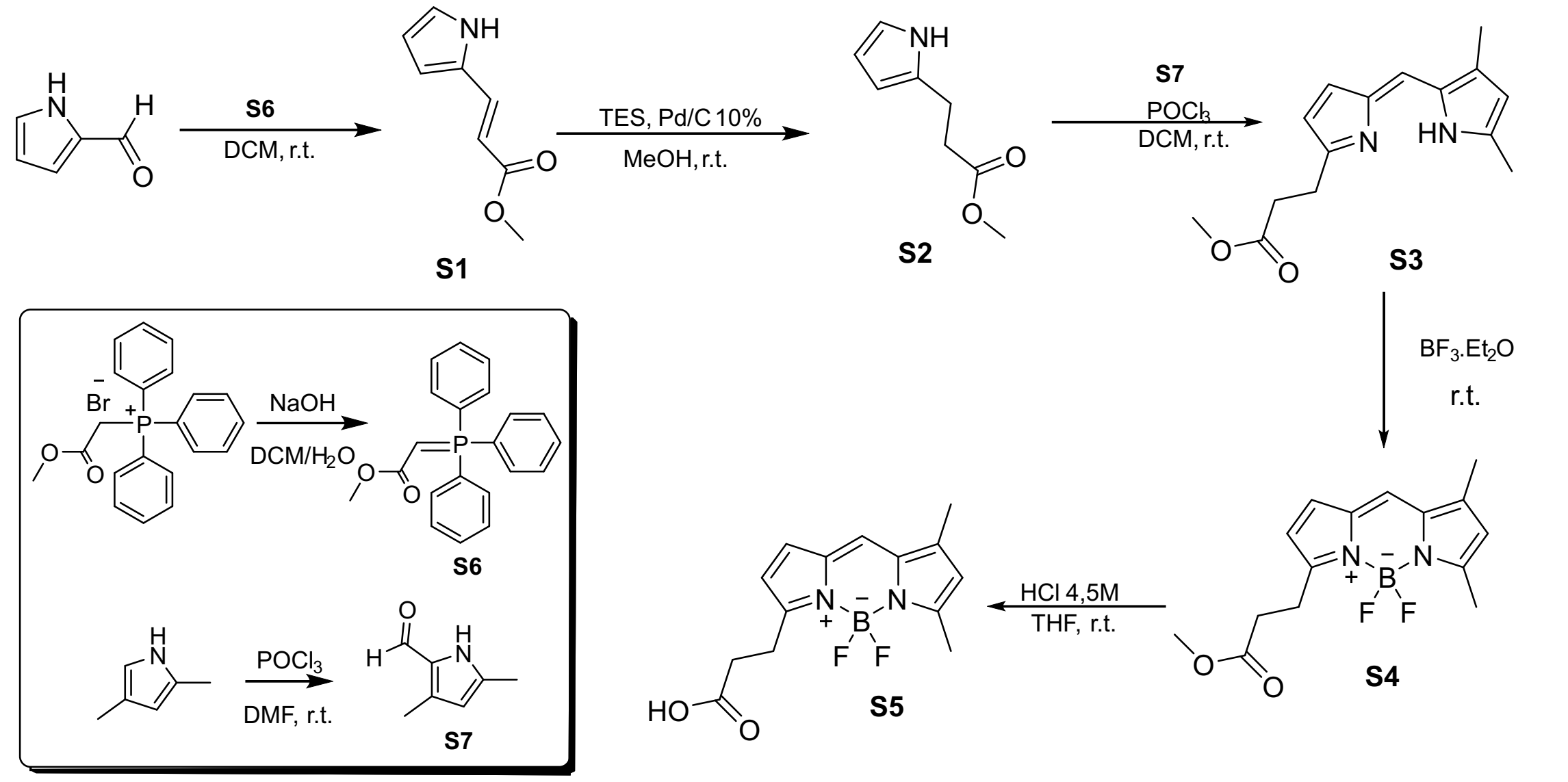

Figure S1. Synthetic strategy to obtain BODIPY-FL (**S5**)

Methyl (E)-3-(1H-pyrrol-2-yl)acrylate (S1) Methoxycarbonylmethyl)triphenylphosphoniumbromide (4.5g, 10.6 mmol, 2 eq.) was dissolved in water (50 ml) and DCM (75 ml) and shaken vigorously in a separatory funnel. The organic phase was recovered, and the aqueous phase was extracted with DCM (2 x 50 ml). The organic layers were combined, dried over Na2SO4, filtered, and evaporated to obtain compound **S6** as a white solid with a yield of 96%. Pyrrole-2-carboxaldehyde (495 mg, 10.1 mmol, 1 eq.), was added to a solution compound **S6** (3.43 g, 5 mmol, 2 eq.) in dry DCM (25 ml) and the reaction was stirred for 24h. The solvent was evaporated and a mixture of Et_2O/H_2O 1:1 (50 ml) was added. The organic phase was washed with water (60 ml) and brine (60 ml), dried over Na_2SO_4, filtered and evaporated. The residue was purified by flash chromatography using silica-gel and as eluent a gradient of hexane/ethyl acetate (from 100% to 0% of hexane) to yield a light pink solid (613 mg, 81%). ^{1}H NMR (300 MHz, DMSO-*d*$_6$) δ 11.51 (bs, 1H), 7.45 (d, *J* = 15.9 Hz, 1H), 7.02 (s, 1H), 6.56 (s, 1H), 6.20 (d, *J* = 16.0 Hz, 1H), 6.16 (s, 1H), 3.67 (s, 3H). ^{13}C NMR (75 MHz, DMSO-*d*$_6$) δ 167.4, 134.8, 128.0, 123.5, 114.9, 110.1, 109.7, 51.0.

Methyl 3-(1H-pyrrol-2-yl)propanoate (S2) To a stirred solution of compound **S1** (300 mg, 1.98 mmol, 1 eq.) and 10% Pd/C (30 mg) in methanol (2 ml) under inert atmosphere, tryethylsilane (3.17 ml, 19.6 mmol, 10 eq.) was slowly added and the reaction was stirred for 10 min. The mixture was filtered through celite and the solvent removed in vacuo. The residue was purified by flash chromatography using silica-gel to yield a colourless

oil (283 mg, 93%). ^{1}H NMR (300 MHz, $CDCl_3$) *δ* 8.66 (bs, 1H), 6.77 – 6.67 (m, 1H), 6.25 – 6.14 (m, 1H), 6.06 – 5.95 (m, 1H), 3.77 (s, 3H), 2.99 (t, *J* = 7.1 Hz, 2H), 2.72 (t, *J* = 7.1 Hz, 2H).

3,5-dimethylpyrrole-2-carbaldehyde (S7) To a solution of 3,5-dimethylpyrrole (200 mg, 2.10 mmol, 1 eq.) in DMF (5 ml), $POCl_3$ (255 µl, 2.73 mmol, 1.3 eq.) was slowly added and the temperature kept below 15°C, the reaction was stirred for 1.5h. Water (5 ml) and 20% KOH solution (10 ml) were added and the aqueous phase was extracted with EtOAc (3 x 25 ml). The organic phase was washed with water (50 ml) and brine (50 ml), dried over Na_2SO_4, filtered, and evaporated. The residue was purified by flash chromatography using silica-gel and as eluent a gradient of hexane/ethyl acetate (from 100% to 0% of hexane) to yield a light-yellow solid (215 mg, 83%). ^{1}H NMR (300 MHz, $CDCl_3$) *δ* 9.60 (bs, 1H), 9.45 (s, 1H), 5.86 (d, *J* = 2.4 Hz, 1H), 2.32 (s, 3H), 2.29 (s, 3H). ^{13}C NMR (75 MHz, $CDCl_3$) *δ* 176.5, 139.1, 135.3, 129.3, 112.6, 13.7, 11.1.

Methyl 3-(5,5-difluoro-7,9-dimethyl-5H-4l4,5l4-dipyrrolo[1,2-c:2',1'-f][1,3,2]diazaborinin-3-yl)propanoate (S4) To a solution of compound **S2** (200 mg, 1.31 mmol, 1 eq.) and compound **S7** (177 mg, 1.44 mmol, 1.1 eq.) in DCM (20 ml) at 0°C, was added a solution of $POCl_3$ (132 µl 1.42 mmol, 1.1 eq.) in DCM (1 ml). The reaction was stirred at 0°C for 30 min and 6 h at room temperature under inert atmosphere. After consuming the starting material, $BF_3.OEt_2$ (645 µl, 5.22 mmol, 4 eq.) and DIPEA (910 µl, 5.22 mmol, 4 eq.) were added at 0°C, and the reaction was stirred 12 h at room temperature. Water was added (40 ml) and was extracted with DCM (3 x 30 ml). The organic phases were combined and were washed with water (40 ml) and brine (40 ml), dried over Na_2SO_4, filtered, and evaporated. The residue was purified by flash chromatography using silica-gel and as eluent a gradient of hexane/ethyl acetate (from 100% to 0% of hexane) to yield dark red solid (303 mg, 76%). ^{1}H NMR (300 MHz, $CDCl_3$) *δ* 7.08 (s, 1H), 6.88 (d, *J* = 3.8 Hz, 1H), 6.26 (d, *J* = 3.8 Hz, 1H), 6.11 (s, 1H), 3.69 (s, 3H), 3.30 (t, *J* = 7.6 Hz, 2H), 2.77 (t, *J* = 7.6 Hz, 2H), 2.57 (s, 3H), 2.25 (s, 3H).

3-(5,5-difluoro-7,9-dimethyl-5H-4l4,5l4-dipyrrolo[1,2-c:2',1'-f][1,3,2]diazaborinin-3-yl)propanoic acid (Bodipy-FL) (S5) To a stirred solution of compound **S4** (250 mg, 0.82 mmol, 1 eq.) in THF (30 ml), HCl 4.5M in dioxane (20 ml) was slowly added and the reaction was stirred for 24h. The mixture was diluted with DCM (60 ml) and the aqueous

phase was extracted with DCM (3x30 ml). The organic phases were combined, dried over Na_2SO_4, filtered, and evaporated. The residue was purified by flash chromatography using silica-gel and as eluent a gradient of dichlromethane/methanol (from 100% to 90% of dichloromethane) to yield a red solid (217 mg, 91%). ^1H NMR (300 MHz, $CDCl_3$) δ 7.09 (s, 1H), 6.88 (d, J = 4.0 Hz, 1H), 6.29 (d, J = 4.0 Hz, 1H), 6.12 (s, 1H), 3.30 (t, J = 7.6 Hz, 2H), 2.83 (t, J = 7.6 Hz, 2H), 2.57 (s, 3H), 2.25 (s, 3H).

Synthesis of qABPs

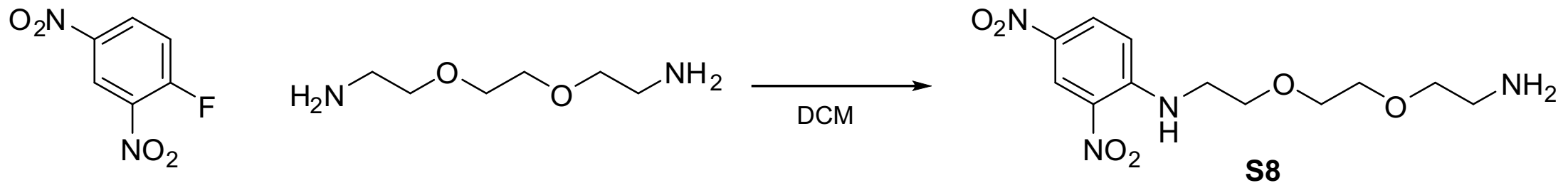

Figure S2. Synthetic strategy to obtain compound **S8**.

N-(2-(2-(2-aminoethoxy)ethoxy)ethyl)-2,4-dinitroaniline (S8) A solution of dinitrofluorobenzene (680 µl, 5.4 mmol, 1 eq.) in 20 ml of DCM was added dropwise to a stirring solution of 2,2'-(ethylenedioxy)bisethylenediamine (7.84 ml, 54 mmol) in 40 mL of DCM at 0 °C. After the addition was complete, the flask was brought to room temperature, and the mixture was stirred for 30 minutes. Then, water was added (200 ml) and the organic phase was recovered. The organic phase was mixed with 200 ml of 0.1 M HCl and the product was taken into the acidic aqueous phase and the DCM phase was discarded. Finally, the pH of the aqueous phase was adjusted to 12 with NaOH, and the product was extracted into the organic phase using 2x200 ml of DCM. Organic phases were combined, washed with brine (200 ml), dried over Na_2SO_4, filtered, and evaporated to yield a yellow oil. ^1H NMR (300 MHz, $CDCl_3$) δ 9.12 (d, J = 2.5 Hz, 1H), 8.79 (s, 1H), 8.25 (dd, J = 9.5, 2.5 Hz, 1H), 6.94 (d, J = 9.5 Hz, 1H), 3.83 (t, J = 5.3 Hz, 2H), 3.77 – 3.59 (m, 6H), 3.53 (t, J = 5.2 Hz, 2H), 2.88 (t, J = 5.2 Hz, 2H), 1.97 (s, 2H).

(3S,4R)-3-((R)-1-((tert-butyldimethylsilyl)oxy)ethyl)-4-(prop-2-yn-1-yl)azetidin-2-one (2). 3-Bromoprop-1-yne (9.2 M solution in toluene) (1.13ml, 10.44 mmol, 3 eq.), zinc powder (2.27g, 34.79 mmol, 10 eq.) and compound **1** (1g, 3.48 mmol, 1 eq.) in THF (35 ml) were added in a reaction flask under inert atmosphere and the reaction was stirred at reflux temperature for 15 min. The reaction was allowed to cold to room temperature and was filtered through celite. The solvent was evaporated, and ethyl acetate was added. Saturated ammonium chloride solution (80 ml) was added to the reaction mixture and extracted with EtOAc (3 × 80 ml). The organic layer was washed with brine, dried over anhydrous Na_2SO_4, filtered, and concentrated under reduced pressure. The obtained residue

was purified by flash chromatography using silica-gel and as eluent a gradient of hexane/ethyl acetate (from 100% to 0% of hexane) to yield a white solid (848 mg, 85%). Adapted from the literature. [33] ^{1}H NMR (300 MHz, $CDCl_3$) δ 6.03 (bs, 1H), 4.20 (qd, J = 6.3, 4.5 Hz, 1H), 3.85 (td, J = 6.2, 2.2 Hz, 1H), 2.90 (ddd, J = 4.4, 2.2, 0.9 Hz, 1H), 2.62 – 2.43 (m, 2H), 2.05 (t, J = 2.6 Hz, 1H), 1.23 (d, J = 6.2 Hz, 3H), 0.87 (s, 9H), 0.07 (s, 3H), 0.07 (s, 3H). ^{13}C NMR (75 MHz, $CDCl_3$) δ 168.1, 79.7, 70.9, 65.1, 63.9, 48.8, 25.7, 24.6, 17.9, -4.3, -5.0. $[\alpha]_D^{24}$= -54.5 (*c* 1.3, $CHCl_3$)

(3S,4R)-3-((R)-1-((tert-butyldimethylsilyl)oxy)ethyl)-1-(hydroxymethyl)-4-(prop-2-yn-1-yl)azetidin-2-one (3). Formaldehyde (35% aqueous solution) (600 μl, 7 mmol, 3.75 eq.), compound **2** (500 mg, 1.9 mmol, 1eq.) and potassium carbonate (129 mg, 0.95 mmol, 0.5eq.) were added to a reaction flask, ethanol (20 ml) was added and the reaction was stirred under reflux for 1 h, then stirred at room temperature overnight. The reaction was filtered, and the solvent was evaporated under reduced pressure. DCM (60 ml) was added to the resulting residue, washed with water and brine (2 × 60 ml), dried over anhydrous Na_2SO_4, filtered, and concentrated under reduced pressure. The residue was purified by flash chromatography using silica-gel and as eluent a gradient of hexane/ethyl acetate (from 100% to 0% of hexane) to yield a white solid (203 mg, 91%).^{1}H NMR (300 MHz, $CDCl_3$) δ 4.75 (d, J = 11.6 Hz, 1H), 4.64 (d, J = 11.6 Hz, 1H), 4.19 (qd, J = 6.2, 4.4 Hz, 1H), 3.93 (td, J = 5.5, 2.2 Hz, 1H), 2.94 (dd, J = 4.4, 2.2 Hz, 1H), 2.63 (dd, J = 5.6, 2.6 Hz, 2H), 2.08 (t, J = 2.6 Hz, 1H), 1.21 (d, J = 6.3 Hz, 3H), 0.85 (s, 9H), 0.05 (s, 3H), 0.05 (s, 3H). ^{13}C NMR (75 MHz, $CDCl_3$) δ 167.7, 79.9, 71.4, 65.1, 64.2, 63.1, 52.1, 25.8, 23.1, 22.8, 18.0, -4.2, -4.9. LRMS (ESI) m/z calcd. for $C_{15}H_{28}NO_3Si$ $[M+H]^+$: 298.5 found: 298. $[\alpha]_D^{24}$= -69.4 (*c* 1.0, $CHCl_3$)

4-nitrophenyl (2-(2-(2-((2,4-dinitrophenyl)amino)ethoxy)ethoxy)ethyl)carbamate (4). Compound **S8** (200 mg, 0.64 mmol, 1.0 eq.) and DMAP (156 mg, 1.28 mmol, 2.0 eq.) were dissolved in acetonitrile (1 ml), the solution was added dropwise to a solution of *p*-nitrophenyl chloroformate (142 mg, 0.7 mmol, 1.1 eq.) in acetonitrile (1 ml) and the resulting solution was stirred at 50 °C for 3 h. The reaction mixture was then diluted in DCM (10 ml) and washed with 0.5 N HCl (60 ml). The aqueous layer was washed with DCM (5 × 10 ml) and all the organic fractions were collected, dried over Na_2SO_4, filtered, and evaporated. The residue was purified by flash chromatography using silica-gel and as eluent a gradient of hexane/ethyl acetate (from 100% to 0% of hexane) to yield a yellow oil (227 mg, 74%). ^{1}H NMR (300 MHz, $CDCl_3$) δ 9.08 (d, J = 2.6

Hz, 1H), 8.88 (bs, 1H), 8.29 – 8.16 (m, 3H), 7.28 (d, *J* = 9.4 Hz, 2H), 6.92 (d, *J* = 9.4 Hz, 1H), 5.87 (t, *J* = 5.3 Hz, 1H), 3.85 (t, *J* = 5.3 Hz, 2H), 3.76-3.70 (m, 3H), 3.74 – 3.57 (m, 5H), 3.51 (t, *J* = 5.4 Hz, 2H). ^{13}C NMR (75 MHz, $CDCl_3$) δ 156.1, 153.4, 148.4, 144.7, 136.2, 130.5, 126.2, 125.2, 124.4, 122.1, 114.1, 70.8, 70.3, 70.0, 68.3, 43.2, 41.3.

Sodium 1-amino-4-((4-(carboxymethyl)phenyl)amino)-9,10-dioxo-9,10-dihydroanthracene-2-sulfonate (cAB40) (6). A solution of bromaminic acid sodium salt (500 mg, 1.23 mmol, 1.12 eq.), 2-(4-aminophenyl)acetic acid 8 (166 mg, 1.1 mmol, 1 eq.), copper (II) sulphate pentahydrate (45 mg, 0.179 mmol, 0.16 eq.) and sodium carbonate (197 mg, 1.58 mmol, 1.44 eq.) in 50 ml of water was stirred under reflux for 24 h. The reaction colour changed from red to purple/dark blue. The reaction mixture was washed with DCM (3 × 50 ml) and the aqueous layer was evaporated under reduced pressure. The resulting dark solid was dissolved in methanol, filtered and the methanol solution was evaporated in vacuo. The obtained residue was purified by reverse-phase column chromatography using as eluent a gradient of water/methanol (from 100% to 85% of water) to yield a dark blue solid (219 mg, 30%).[37] ^{1}H NMR (300 MHz, DMSO-d_6) δ 12.14 (s, 1H), 10.16 (bs, 1H), 8.34 – 8.20 (m, 2H), 7.99 (s, 1H), 7.91 – 7.78 (m, 2H), 7.48 (bs, 1H), 7.31 (d, *J* = 8.3 Hz, 2H), 7.14 (d, *J* = 8.3 Hz, 2H), 3.27 (s, 2H). ^{13}C NMR (75 MHz, DMSO-d_6) δ 182.0, 181.7, 144.2, 142.9, 141.7, 136.7, 135.9, 134.1, 133.6, 133.0, 132.7, 130.4, 126.0, 125.9, 122.9, 122.6, 110.7, 109.0, 52.6. LRMS (ESI) *m/z* calcd. for $C_{22}H_{15}N_2O_7S^-$ [M]: 451.4 found: 451.1.

((3S,4R)-3-((R)-1-((tert-butyldimethylsilyl)oxy)ethyl)-2-oxo-4-(prop-2-yn-1-yl)azetidin-1-yl)methyl (2-(2-(2-((2,4-dinitrophenyl)amino)ethoxy)ethoxy)ethyl)carbamate (7i). Compound **4** (200 mg, 0.42 mmol, 1.0 eq.) and DMAP (102 mg, 0.84 mmol, 2.0 eq.) were dissolved in DCM (1 ml), the solution was added dropwise to a solution of compound **3** (137 mg, 0.46 mmol, 1.1 eq.) in DCM (1 ml) and the resulting solution was stirred for 4 h at room temperature. DCM was added (10 ml) and washed with water and brine (2 x 10 ml), dried over Na2SO4, filtered, and evaporated. The residue was purified by flash chromatography using silica-gel and as eluent a gradient of hexane/ethyl acetate (from 100% to 0% of hexane) to yield a yellow oil (216 mg, 81%). ^{1}H NMR (300 MHz, $CDCl_3$) δ 9.14 (d, *J* = 2.7 Hz, 1H), 8.81 (s, 1H), 8.28 (dd, *J* = 9.5, 2.7 Hz, 1H), 6.94 (d, *J* = 9.5 Hz, 1H), 5.24 (s, 1H), 5.10 (d, *J* = 11.6 Hz, 1H), 5.03 (d, *J* = 11.6 Hz, 1H), 4.22 (qd, *J* = 6.3, 3.8 Hz, 1H), 4.05 – 3.91 (m, 1H), 3.83 (t, *J* =

5.2 Hz, 2H), 3.75 – 3.51 (m, 8H), 3.48 – 3.27 (m, 2H), 3.04 (dd, *J* = 3.8, 2.3 Hz, 1H), 2.81 – 2.58 (m, 2H), 2.01 (t, *J* = 2.6 Hz, 1H), 1.20 (d, *J* = 6.3 Hz, 3H), 0.84 (s, 9H), 0.05 (s, 3H), 0.05 (s, 3H). ^{13}C NMR (75 MHz, $CDCl_3$) δ 168.2, 156.1, 148.4, 136.1, 130.5, 130.4, 124.4, 114.2, 79.0, 71.5, 70.7, 70.3, 70.1, 68.5, 64.6, 63.1, 62.6, 52.1, 43.2, 40.9, 25.8, 25.8, 21.8, 17.9, -4.2, -5.1. $[\alpha]_D^{24}$= -46.3 (*c* 1.0, $CHCl_3$)

((3S,4R)-3-((R)-1-((tert-butyldimethylsilyl)oxy)ethyl)-2-oxo-4-(prop-2-yn-1-yl)azetidin-1-yl)methyl (3,5-dinitrophenyl)carbamate (7ii). A solution of 3,5-dinitrobenzoyl azide **S10** (300 mg, 1.27 mmol, 3eq.), in dry toluene (3 ml) was heated to reflux for 3 hours. The reaction was cooled to room temperature and a solution of compound **3** (126 mg, 0.43 mmol, 1 eq.) in dry ACN was added. The reaction was stirred at room temperature overnight. The solvent was evaporated, and the residue was purified by flash chromatography using silica-gel and as eluent a gradient of hexane/ethyl acetate (from 100% to 0% of hexane) to yield a colourless oil (136 mg, 63%). ^{1}H NMR (300 MHz, $CDCl_3$) δ 9.56 (s, 1H), 8.85 (d, *J* = 2.0 Hz, 2H), 8.71 (t, *J* = 2.0 Hz, 1H), 5.33 (d, *J* = 11.7 Hz, 1H), 5.16 (d, *J* = 11.7 Hz, 1H), 4.35 – 4.18 (m, 1H), 4.12 (dt, *J* = 4.3, 2.1 Hz, 1H), 3.24 (dd, *J* = 4.7, 2.3 Hz, 1H), 3.04 (ddd, *J* = 17.7, 5.1, 2.7 Hz, 1H), 2.75 (dt, *J* = 17.7, 2.7 Hz, 1H), 2.07 (t, *J* = 2.7 Hz, 1H), 1.28 (d, *J* = 6.2 Hz, 3H), 0.77 (s, 9H), 0.02 (s, 3H), -0.02 (s, 3H). ^{13}C NMR (75 MHz, $CDCl_3$) δ 169.7, 153.7, 149.0, 141.2, 118.3, 112.8, 78.1, 72.2, 65.2, 63.5, 62.1, 53.7, 25.7, 20.9, 17.9, -4.1, -5.1. $[\alpha]_D^{24}$= -46.6 (*c* 1.0, $CHCl_3$)

1-amino-4-((4-(2-(((3S,4R)-3-((R)-1-((tert-butyldimethylsilyl)oxy)ethyl)-2-oxo-4-(prop-2-yn-1-yl)azetidin-1-yl)methoxy)-2-oxoethyl)phenyl)amino)-9,10-dioxo-9,10-dihydroanthracene-2-sulfonate (7iii). To a solution of cAB40 **6** (120 mg, 0.27 mmol, 1 eq.), TBTU (193 mg, 0.8 mmol, 2.2 eq.), HOBt (44 mg, 0.33 mmol, 1.2 eq.) and DIPEA (188 μl, 1.1 mmol, 4 eq.) in dry DMF (1 ml) under inert atmosphere, compound **3** was added and the reaction was stirred for 1 h at 0 ºC and at room temperature overnight. The mixture was purified by flash reverse-phase column chromatography to yield a dark blue solid (103 mg, 65%). The compound was obtained with trace amounts of starting material. The mixture was used in the following reaction steps without further purification. LRMS (ESI) m/z calcd. for $C_{37}H_{40}N_3O_9SSiNa$ $[M+Na]^+$:730.2 found: 730.

((3S,4R)-3-((R)-1-hydroxyethyl)-2-oxo-4-(prop-2-yn-1-yl)azetidin-1-yl)methyl (2-(2-(2-((2,4-dinitrophenyl)amino)ethoxy)ethoxy)ethyl)carbamate (8i). To a solution of compound **7i** (150 mg, 0.24 mmol, 1 eq.) in THF (1 ml), acetic acid (270 μl, 0.23 mmol, 20 eq.) was added dropwise. Then, a solution of TBAF (1M in THF)

(9 ml, 9.41 mmol, 40 eq.) was also added dropwise and stirred under inert atmosphere for 24 h. The reaction was diluted in EtOAc (20 ml) and cold saturated sodium hydrogen carbonated solution (20 ml) was added. The mixture was extracted with EtOAc (3 × 10 ml) and the organic layer was washed with brine (2 × 10 ml), dried over Na_2SO_4, filtered and evaporated. The residue was purified by flash chromatography using silica-gel and as eluent a gradient of hexane/ethyl acetate (from 100% to 0% of hexane) to yield a yellow oil (87 mg, 71%). ^{1}H NMR (300 MHz, $CDCl_3$) δ 9.15 (d, J = 2.5 Hz, 1H), 8.82 (s, 1H), 8.28 (dd, J = 9.5, 2.5 Hz, 1H), 6.95 (d, J = 9.5 Hz, 1H), 5.37 (s, 1H), 5.14 (d, J = 11.6 Hz, 1H), 5.03 (d, J = 11.6 Hz, 1H), 4.20 (m, 1H), 3.95 (td, J = 5.0, 2.4 Hz, 1H), 3.83 (t, J = 5.2 Hz, 2H), 3.75 – 3.53 (m, 8H), 3.38 (m, 2H), 3.09 (dd, J = 5.1, 2.4 Hz, 1H), 2.71 (dd, J = 5.0, 2.6 Hz, 2H), 2.05 (t, J = 2.6 Hz, 1H), 1.29 (d, J = 6.4 Hz, 3H). ^{13}C NMR (75 MHz, $CDCl_3$) δ 168.4, 156.1, 148.4, 136.0, 130.4, 130.4, 124.3, 114.2, 78.8, 71.6, 70.7, 70.2, 70.0, 68.4, 64.3, 63.2, 62.3, 52.5, 43.2, 40.9, 21.8. $[\alpha]_D^{24}$= -34.7 (c 1.0, $CHCl_3$)

((3S,4R)-3-((R)-1-hydroxyethyl)-2-oxo-4-(prop-2-yn-1-yl)azetidin-1-yl)methyl (3,5-dinitrophenyl)carbamate (8ii). To a solution of compound **7ii** (100 mg, 0.20 mmol, 1 eq.) in THF (0.7 ml), acetic acid (170 μl, 2.96 mmol, 15 eq.) was added dropwise. Then, a solution of TBAF (1M in THF) (5.9 ml, 5.92 mmol, 30 eq.) was also added dropwise and stirred under inert atmosphere for 48 h. The reaction was diluted in EtOAc (15 ml) and cold saturated sodium hydrogen carbonated solution (15 ml) was added. The mixture was extracted with EtOAc (3 × 8 ml) and the organic layer was washed with brine (2 × 8 ml), dried over Na_2SO_4, filtered and evaporated. The residue was purified by flash chromatography using silica-gel and as eluent a gradient of hexane/ethyl acetate (from 100% to 0% of hexane) to yield a yellow oil (51 mg, 65%). ^{1}H NMR (300 MHz, $CDCl_3$) δ 9.14 (s, 1H), 8.67 (d, J = 1.9 Hz, 2H), 8.59 (t, J = 1.9 Hz, 1H), 5.28 (d, J = 11.7 Hz, 1H), 5.18 (d, J = 11.7 Hz, 1H), 4.22 (s, 1H), 4.13 – 3.97 (m, 1H), 3.17 (dd, J = 4.5, 2.4 Hz, 1H), 2.92 – 2.57 (m, 3H), 2.03 (t, J = 2.4 Hz, 2H), 1.25 (d, J = 6.4 Hz, 3H). ^{13}C NMR (75 MHz, $CDCl_3$) δ 170.3, 155.2, 146.1, 139.4, 115.2, 113.6, 79.7, 72.5, 71.5, 66.8, 55.2, 54.6, 20.5, 19.3. $[\alpha]_D^{24}$= -47.2 (c 1.0, $CHCl_3$)

1-amino-4-((4-(2-(((3S,4R)-3-((R)-1-hydroxyethyl)-2-oxo-4-(prop-2-yn-1-yl)azetidin-1-yl)methoxy)-2-oxoethyl)phenyl)amino)-9,10-dioxo-9,10-dihydroanthracene-2-sulfonate tetrabutylammonium salt (8iii). To a solution of compound **7iii** (22 mg, 0.03 mmol, 1 eq.) in THF (500 μl ml), acetic acid (13 μl,

0.22 mmol, 7 eq.) was added dropwise. Then, a solution of TBAF (1M in THF) (420 *µ*l, 14 mmol, 14 eq.) was also added dropwise and stirred under inert atmosphere for 72 h. The solvent was evaporated, and the residue purified by flash reverse-phase column chromatography using as eluent a gradient of water/methanol (from 100% to 10% of water) to yield a blue oil (16 mg, 72%). The compound was isolated as a tetrabutylammonium salt. ^{1}H NMR (300 MHz, Acetone-d_6) δ 12.25 (s, 1H), 8.40 – 8.28 (m, 3H), 7.86 – 7.74 (m, 2H), 7.39 (d, J = 8.5 Hz, 2H), 7.29 (d, J = 8.5 Hz, 2H), 5.25 (d, J = 11.4 Hz, 1H), 5.17 (d, J = 11.4 Hz, 1H), 4.04-3.96 (m, 1H), 3.86 (td, J = 4.7, 2.5 Hz, 1H), 3.71 (s, 2H), 3.47 – 3.37 (m, 8H), 3.31 (s, 2H), 3.00 (dd, J = 6.6, 2.4 Hz, 1H), 2.75 (dd, J = 5.0, 2.7 Hz, 1H), 2.69 (dd, J = 4.3, 2.7 Hz, 1H), 2.49 (t, J = 2.7 Hz, 1H), 1.87 – 1.72 (m, 8H), 1.47-1.34 (m, 8H), 1.23 (d, J = 6.3 Hz, 3H), 0.95 (t, J = 7.3 Hz, 12H).

N-(2-(2-(2-(2-azidoethoxy)ethoxy)ethoxy)ethyl)-3-(5,5-difluoro-7,9-dimethyl-5H-4l4,5l4-dipyrrolo[1,2-c:2',1'-f][1,3,2]diazaborinin-3-yl)propanamide (9). Compound **S5** (120 mg, 0.41 mmol, 1 eq.) and COMU (184 mg, 0.43 mmol, 1.05 eq.) were dissolved in dry DCM (3 ml). DIPEA (214 *µ*l, 1.23 mmol, 3 eq.) was added and the reaction was stirred at room temperature for 10 min. 11-Azido-3,6,9-trioxaundecan-1-amine (90 *µ*l, 0.45 mmol, 1.1 eq.) was added and the reaction was stirred for 30 min until all the starting material was consumed. The solvent was evaporated, and the residue was purified by flash chromatography using silica-gel and as eluent a gradient of hexane/ethyl acetate (from 100% to 0% of hexane) to yield an orange oil (129 mg, 64%). ^{1}H NMR (300 MHz, Acetone-d_6) δ 7.52 (d, J = 9.1 Hz, 2H), 7.06 (d, J = 4.0 Hz, 1H), 6.37 (d, J = 4.0 Hz, 1H), 6.25 (s, 1H), 3.72 – 3.56 (m, 16H), 3.23 (t, J = 7.7 Hz, 2H), 2.63 (t, J = 7.7 Hz, 2H), 2.51 (s, 3H), 2.28 (s, 3H). ^{13}C NMR (75 MHz, Acetone-d_6) δ 172.5, 159.9, 158.5, 144.6, 135.6, 133.9, 129.2, 125.7, 120.7, 117.1, 70.6, 70.4, 70.4, 70.3, 70.0, 66.6, 39.5, 34.8, 14.4, 10.8.

((2R,3S)-2-((1-(15-(5,5-difluoro-7,9-dimethyl-5H-4l4,5l4-dipyrrolo[1,2-c:2',1'-f][1,3,2]diazaborinin-3-yl)-13-oxo-3,6,9-trioxa-12-azapentadecyl)-1H-1,2,3-triazol-4-yl)methyl)-3-((R)-1-hydroxyethyl)-4-oxoazetidin-1-yl)methyl (2-(2-(2-((2,4-dinitrophenyl)amino)ethoxy)ethoxy)ethyl)carbamate (10). A solution of compound **8i** (25 mg, 0.05 mmol, 1 eq.), copper (II) sulphate (0.2 M aqueous solution) (115 *µ*l, 0.02mmol, 0.48 eq.) and sodium ascorbate (0.2 M aqueous solution) (115 *µ*l, 0.02 mmol, 0.48 eq.) in MeOH (1 ml) was stirred for 10 min. Then, compound **9** (26 mg, 0.05 mmol, 1.1 eq.) was added and the reaction was

stirred at room temperature for 2h. The solvent was evaporated, and the residue was purified by flash chromatography using silica-gel and as eluent a gradient of hexane/ethyl acetate (from 100% to 0% of hexane) to yield an orange oil (39 mg, 81%). ^{1}H NMR (300 MHz, Acetone-d_6) δ 8.96 (d, J = 2.7 Hz, 1H), 8.87 (s, 1H), 8.27 (ddd, J = 9.6, 2.7, 0.6 Hz, 1H), 7.91 (s, 1H), 7.49 (s, 1H), 7.28 (d, J = 9.6 Hz, 2H), 7.03 (d, J = 4.0 Hz, 1H), 6.47 (t, J = 5.7 Hz, 1H), 6.38 (d, J = 4.0 Hz, 1H), 6.24 (s, 1H), 5.14 (d, J = 11.5 Hz, 1H), 5.06 (d, J = 11.5 Hz, 1H), 4.56 – 4.50 (m, 2H), 4.09 – 3.90 (m, 2H), 3.87-3.82 (m, 4H), 3.73 (t, J = 5.1 Hz, 2H), 3.69 – 3.59 (m, 4H), 3.59 – 3.47 (m, 12H), 3.41 – 3.34 (m, 3H), 3.32-3.25 (m, 3H), 3.22 (t, J = 7.6 Hz, 2H), 3.04 – 2.95 (m, 2H), 2.60 (t, J = 7.6 Hz , 2H), 2.50 (s, 3H), 2.28 (s, 3H), 1.06 (d, J = 6.3 Hz, 3H). ^{13}C NMR (75 MHz, Acetone-d_6) δ 171.9, 168.0, 164.1, 160.2, 159.3, 156.8, 149.9, 149.5, 144.8, 143.3, 136.4, 135.7, 135.2, 134.3, 131.0, 130.7, 129.5, 125.5, 124.3, 120.9, 117.7, 116.1, 71.0, 71.0, 71.0, 70.9, 70.8, 70.4, 70.0, 69.3, 65.9, 64.1, 63.8, 55.9, 50.6, 43.9, 41.4, 39.8, 35.2, 14.8, 11.2. HRMS (ESI) m/z calcd. for $C_{44}H_{60}BF_2N_{11}O_{14}$ $[M+H]^+$: 1016.4462 found: 1016.4507. $[\alpha]_D^{24}$= -64.8 (c 0.7, EtOH)

((2R,3S)-2-((1-(15-(5,5-difluoro-7,9-dimethyl-5H-4l4,5l4-dipyrrolo[1,2-c:2',1'-f][1,3,2]diazaborinin-3-yl)-13-oxo-3,6,9-trioxa-12-azapentadecyl)-1H-1,2,3-triazol-4-yl)methyl)-3-((R)-1-hydroxyethyl)-4-oxoazetidin-1-yl)methyl (3,5-dinitrophenyl)carbamate (11). A solution of compound **8ii** (20 mg, 0.05 mmol, 1 eq.), copper (II) sulphate (0.2 M aqueous solution) (122 μl, 0.02mmol, 0.48 eq.) and sodium ascorbate (0.2 M aqueous solution) (122 μl, 0.02 mmol, 0.48 eq.) in MeOH (1 ml) was stirred for 10 min. Then, compound **9** (27 mg, 0.06 mmol, 1.1 eq.) was added and the reaction was stirred at room temperature for 2h. The solvent was evaporated, and the residue was purified by flash chromatography using silica-gel and as eluent a gradient of hexane/ethyl acetate (from 100% to 0% of hexane) to yield an orange oil (34 mg, 75%). ^{1}H NMR (300 MHz, Acetone-d_6) δ 9.95 (s, 1H), 8.83 (d, J = 2.0 Hz, 2H), 8.51 (t, J = 2.0 Hz, 1H), 7.97 (s, 1H), 7.44 (s, 1H), 7.42 (t, J = 5.5 Hz, 1H), 6.99 (d, J = 4.0 Hz, 1H), 6.35 (d, J = 4.0 Hz, 1H), 6.22 (s, 1H), 5.37 (d, J = 11.5 Hz, 1H), 5.31 (d, J = 11.5 Hz, 1H), 4.59 – 4.47 (m, 2H), 4.18 (ddd, J = 7.2, 4.4, 2.4 Hz, 1H), 4.09-3.97 (m, 1H), 3.86 (t, J = 5.1 Hz, 2H), 3.59-3.49 (m, 11H), 3.46 – 3.34 (m, 2H), 3.20 (t, J = 7.6 Hz, 2H), 3.10 (dd, J = 15.1, 7.8 Hz, 1H), 3.05 – 3.00 (m, 1H), 2.66 – 2.57 (m, 3H), 2.46 (s, 3H), 2.26 (s, 3H), 1.07 (d, J = 6.3 Hz, 3H). ^{13}C NMR (75 MHz, Acetone-d_6) δ 172.4, 168.3, 160.2, 159.1, 153.9, 149.5, 144.8, 143.3, 142.5, 135.6, 134.7, 129.5, 125.4, 124.4, 120.9, 118.5, 117.6, 112.5, 71.0, 71.0, 70.9, 70.8, 70.3, 70.0, 65.6, 65.2, 64.3,

55.9, 55.4, 50.7, 39.9, 35.1, 14.8, 11.1. HRMS (ESI) m/z calcd. for $C_{38}H_{49}BF_2N_{10}O_{12}$ $[M-H]^-$: 885.3515 found: 885.3542. $[\alpha]_D^{24}$= -87.6 (*c* 0.5, EtOH)

1-amino-4-((4-(2-(((2R,3S)-2-((1-(15-(5,5-difluoro-7,9-dimethyl-5H-4l4,5l4-dipyrrolo[1,2-c:2',1'-f][1,3,2]diazaborinin-3-yl)-13-oxo-3,6,9-trioxa-12-azapentadecyl)-1H-1,2,3-triazol-4-yl)methyl)-3-((R)-1-hydroxyethyl)-4-oxoazetidin-1-yl)methoxy)-2-oxoethyl)phenyl)amino)-9,10-dioxo-9,10-dihydroanthracene-2-sulfonate tetrabutylammonium salt (12). A solution of compound **8iii** (14 mg, 16 *µ*mol, 1 eq.), copper (II) sulphate (0.2 M aqueous solution) (39 *µ*l, 8 *µ*mol, 0.48 eq.) and sodium ascorbate (0.2 M aqueous solution) (39 *µ*l, 8 *µ*mol, 0.48 eq.) in MeOH (300 *µ*l) was stirred for 10 min. Then, compound **9** (9 mg, 18 *µ*mol, 1.1 eq.) was added and the reaction was stirred at room temperature for 2h. The solvent was evaporated, and the residue purified by flash reverse-phase column chromatography using as eluent a gradient of water/methanol (from 100% to 0% of water) to yield a blue oil (15 mg, 83%). ^1H NMR (300 MHz, Acetone-d_6) δ 12.08 (s, 1H), 8.25 – 8.14 (m, 3H), 7.83 (s, 1H), 7.71 – 7.62 (m, 2H), 7.34 (s, 1H), 7.23 (d, *J* = 8.5 Hz, 3H), 7.14 (d, *J* = 8.4 Hz, 2H), 6.87 (d, *J* = 4.1 Hz, 1H), 6.23 (d, *J* = 3.9 Hz, 1H), 6.07 (s, 1H), 5.13 (d, *J* = 11.3 Hz, 1H), 5.07 (d, *J* = 11.3 Hz, 1H), 4.52 (s, 1H), 4.43 – 4.33 (m, 2H), 3.90 – 3.78 (m, 2H), 3.72 – 3.65 (m, 2H), 3.55 (s, 2H), 3.46 – 3.33 (m, 11H), 3.33 – 3.16 (m, 10H), 3.13 – 3.00 (m, 3H), 2.89 – 2.77 (m, 2H), 2.50 – 2.42 (m, 3H), 2.35 (s, 3H), 2.13 (s, 3H), 1.75 – 1.59 (m, 8H), 1.36-1.20 (m, 8H), 0.94 (d, *J* = 6.3 Hz, 3H), 0.82 (t, *J* = 7.3 Hz, 12H). $C_{53}H_{57}BF_2N_9O_{13}S^-$ $[M]^-$: 1108.3861 found:1108.3879.

1-(2-(2-(2-((2,4-dinitrophenyl)amino)ethoxy)ethoxy)ethyl)-3-(4-hydroxyphenethyl)urea (13). Compound **4** (350 mg, 0.73 mmol, 1.0 eq.) and DMAP (179 mg, 1.46 mmol, 2.0 eq.) were dissolved in DCM (2 ml), the solution was added dropwise to a solution of tyramine (110 mg, 0.80 mmol, 1.1 eq.) in DCM (2 ml) and the resulting solution was stirred for 3 h at room temperature. DCM was added (20 ml) and washed with water and brine (2 x 20 ml), dried over Na_2SO_4, filtered, and evaporated. The residue was purified by flash chromatography using silica-gel and as eluent a gradient of hexane/ethyl acetate (from 100% to 0% of hexane) to yield a yellow oil (220 mg, 63%). ^1H NMR (400 MHz, $CDCl_3$) δ 9.10 (d, *J* = 2.7 Hz, 1H), 8.97 (s, 1H), 8.30 (dd, *J* = 9.5, 2.7 Hz, 1H), 7.04 (d, *J* = 8.1 Hz, 2H), 6.92 (d, *J* = 9.5 Hz, 1H), 6.74 (d, *J* = 8.1 Hz, 2H), 6.21 (s, 1H), 5.24 – 5.15 (m, 1H), 5.08 (s, 1H), 3.83 (t, *J* = 5.2 Hz, 3H), 3.73 (q, *J* = 3.5 Hz, 2H), 3.67 (q, *J* = 3.4 Hz, 2H), 3.64 – 3.53 (m, 4H), 3.53 – 3.40 (m, 5H), 2.75 (t, *J* = 7.0

Hz, 2H). ^{13}C NMR (100 MHz, $CDCl_3$) δ 158.6, 154.6, 148.4, 130.7, 129.9, 124.5, 115.3, 114.1, 71.2, 70.9, 70.2, 67.8, 43.0, 41.8, 40.3, 35.5.

1-(3,5-dinitrophenyl)-3-(4-hydroxyphenethyl)urea (14). A solution of 3,5-dinitrobenzoyl azide **S10** (850 mg, 3.59 mmol, 2.5 eq.), in dry toluene (8 ml) was heated to reflux for 3 hours. The reaction was cooled to room temperature and a solution of tyramine (200 mg, 1.45 mmol, 1 eq.) in dry ACN (2 ml) was added. The reaction was stirred at room temperature overnight. The solvent was evaporated, and the residue was purified by flash chromatography using silica-gel and as eluent a gradient of hexane/ethyl acetate (from 100% to 0% of hexane) to yield a colourless oil (233 mg, 46%). ^{1}H NMR (300 MHz, DMSO-d_6) δ 9.55 (s, 1H), 9.16 (s, 1H), 8.69 (d, J = 2.1 Hz, 2H), 8.33 (t, J = 2.1 Hz, 1H), 7.08 – 6.99 (m, 2H), 6.76 – 6.63 (m, 2H), 6.52 (t, J = 5.7 Hz, 1H), 3.33-3.27 (m, 2H), 2.66 (t, J = 7.3 Hz, 2H). ^{13}C NMR (75 MHz, DMSO-d_6) δ 157.2, 154.9, 146.7, 138.7, 132.3, 126.5, 117.1, 116.2, 111.3, 40.9, 34.7.

4-(2-((2,4-dinitrophenyl)amino)ethyl)phenol (15). A solution of dinitrofluorobenzene (1,36 ml, 10.8 mmol, 1 eq.) in 40 ml of DCM was added dropwise to a stirring solution of tyramine (1.77 g, 12.9 mmol, 1.2 eq.) in 80 ml of DCM at 0 °C. After the addition was complete, the flask was brought to room temperature, and the mixture was stirred for 2 hours. Then, the solvent was evaporated, and the compound was crystalised with hot ethanol to give an orange solid (2.77 g, 85% yield). ^{1}H NMR (300 MHz, DMSO-d_6) δ 9.24 (s, 1H), 8.84 (d, J = 2.8 Hz, 1H), 8.80 (t, J = 5.9 Hz, 1H), 8.23 (dd, J = 9.7, 2.8 Hz, 1H), 7.25 (d, J = 9.7 Hz, 1H), 7.17 – 6.99 (m, 2H), 6.75 – 6.53 (m, 2H), 3.74 – 3.56 (m, 2H), 2.83 (t, J = 7.3 Hz, 2H).^{13}C NMR (75 MHz, DMSO-d_6) δ 156.4, 148.5, 135.2, 130.4, 130.2, 130.0, 128.7, 124.1, 115.9, 115.7, 45.0, 33.6.

1-(4-(((2R,3R)-3-((R)-1-((tert-butyldimethylsilyl)oxy)ethyl)-4-oxoazetidin-2-yl)oxy)phenethyl)-3-(2-(2-(2-((2,4-dinitrophenyl)amino)ethoxy)ethoxy)ethyl)urea (16i). To a solution of compound **13** (350 mg, 0.73 mmol, 1 eq.) in dry acetone (1.5 ml) a solution of 1M NaOH (880 μl, 0.88 mmol, 1.2 eq.) was added and the reaction was stirred for 15 minutes. A solution of compound **1** (274 mg, 0.95 mmol, 1.3 eq.) in dry acetone (1 ml) was added to the reaction and the reaction was stirred at room temperature for 20 minutes. The solvent was evaporated and water (60 ml) was added. The mixture was extracted with EtOAc (3 × 60 ml) and the organic layer was washed with brine (2 × 60 ml), dried over Na_2SO_4, filtered and evaporated. The residue

was purified by flash chromatography using silica-gel and as eluent a gradient of hexane/ethyl acetate (from 100% to 0% of hexane) to yield an orange oil (449 mg, 87%). ^{1}H NMR (300 MHz, $CDCl_3$) δ 9.08 (d, *J* = 2.7 Hz, 1H), 8.94 (s, 1H), 8.29 (dd, *J* = 9.6, 2.7 Hz, 1H), 7.21 – 7.10 (m, 2H), 6.92 (d, *J* = 9.6 Hz, 1H), 6.85 – 6.77 (m, 2H), 6.52 (s, 1H), 5.63 (d, *J* = 1.2 Hz, 1H), 5.21 – 4.92 (m, 2H), 4.24 (qd, *J* = 6.3, 3.5 Hz, 1H), 3.82 (t, *J* = 5.2 Hz, 2H), 3.75 – 3.68 (m, 2H), 3.68 – 3.53 (m, 6H), 3.51 – 3.29 (m, 4H), 3.23 (dd, *J* = 3.5, 1.2 Hz, 1H), 2.79 (t, *J* = 7.1 Hz, 2H), 1.25 (d, *J* = 6.3 Hz, 3H), 0.87 (s, 9H), 0.08 (s, 3H), 0.06 (s, 3H). ^{13}C NMR (75 MHz, $CDCl_3$) δ 167.7, 162.3, 158.5, 154.7, 148.4, 133.8, 130.7, 130.3, 130.2, 124.4, 116.2, 116.1, 114.2, 71.2, 70.8, 70.1, 67.8, 65.9, 65.4, 64.1, 63.7, 43.0, 41.6, 40.2, 35.6, 25.8, 25.7, 22.5, 21.5, -3.6, -4.3, -5.1. $[\alpha]_D^{24}$= -20.8 (*c* 0.9, $CHCl_3$)

1-(4-(((2R,3R)-3-((R)-1-((tert-butyldimethylsilyl)oxy)ethyl)-4-oxoazetidin-2-yl)oxy)phenethyl)-3-(3,5-dinitrophenyl)urea (16ii). To a solution of compound **14** (200 mg, 0.58 mmol, 1 eq.) in dry acetone (1 ml) a solution of 1M NaOH (696 *µ*l, 0.70 mmol, 1.2 eq.) was added and the reaction was stirred for 15 minutes. A solution of compound **1** (216 mg, 0.75 mmol, 1.3 eq.) in dry acetone (0.5 ml) was added to the reaction and the reaction was stirred at room temperature for 20 minutes. The solvent was evaporated and water (30 ml) was added. The mixture was extracted with EtOAc (3 × 30 ml) and the organic layer was washed with brine (2 × 30 ml), dried over Na_2SO_4, filtered and evaporated. The residue was purified by flash chromatography using silica-gel and as eluent a gradient of hexane/ethyl acetate (from 100% to 0% of hexane) to yield a yellow oil (258 mg, 78%). ^{1}H NMR (300 MHz, $CDCl_3$) δ 8.53 – 8.45 (m, 3H), 8.43 (t, *J* = 2.0 Hz, 1H), 7.34 (s, 1H), 7.09 (d, *J* = 8.6 Hz, 2H), 6.77 (d, *J* = 8.6 Hz, 2H), 5.78 – 5.69 (m, 1H), 5.55 (d, *J* = 1.2 Hz, 1H), 4.21 – 4.10 (m, 1H), 3.57 – 3.31 (m, 2H), 3.12 (dd, *J* = 3.6, 1.2 Hz, 1H), 2.80 – 2.69 (m, 2H), 1.27 – 1.14 (m, 3H), 0.78 (s, 9H), -0.00 (s, 3H), -0.04 (s, 3H). ^{13}C NMR (75 MHz, $CDCl_3$) δ 163.3, 157.9, 155.2, 148.7, 136.1, 131.5, 130.9, 116.6, 115.5, 113.1, 78.2, 67.1, 59.1, 41.6, 33.6, 26.9, 22.7, 14.2, 4.3. $[\alpha]_D^{24}$= -41.5 (*c* 1.0, $CHCl_3$)

(3R,4R)-3-((R)-1-((tert-butyldimethylsilyl)oxy)ethyl)-4-(4-(2-((2,4-dinitrophenyl)amino)ethyl)phenoxy)azetidin-2-one (16iii). To a solution of compound **15** (400 mg, 1.32 mmol, 1 eq.) in dry acetone (2 ml) a solution of 1M NaOH (1.58 ml, 1.58 mmol, 1.2 eq.) was added and the reaction was stirred for 15 minutes. A solution of compound **1** (493 mg, 1.71 mmol, 1.3 eq.) in dry acetone (1.5 ml) was added to the reaction and the reaction was stirred at room

temperature for 20 minutes. The solvent was evaporated and water (60 ml) was added. The mixture was extracted with EtOAc (3 × 60 ml) and the organic layer was washed with brine (2 × 60 ml), dried over Na_2SO_4, filtered and evaporated. The residue was purified by flash chromatography using silica-gel and as eluent a gradient of hexane/ethyl acetate (from 100% to 0% of hexane) to yield an orange solid (644 mg, 92%). ^{1}H NMR (300 MHz, $CDCl_3$) δ 8.53 – 8.45 (m, 3H), 8.43 (t, J = 2.0 Hz, 1H), 7.34 (s, 1H), 7.09 (d, J = 8.6 Hz, 2H), 6.77 (d, J = 8.6 Hz, 2H), 5.78 – 5.69 (m, 1H), 5.55 (d, J = 1.2 Hz, 1H), 4.21 – 4.10 (m, 1H), 3.57 – 3.31 (m, 2H), 3.12 (dd, J = 3.6, 1.2 Hz, 1H), 2.80 – 2.69 (m, 2H), 1.27 – 1.14 (m, 3H), 0.78 (s, 9H), -0.00 (s, 3H), -0.04 (s, 3H). 13C NMR (75 MHz, $CDCl_3$) δ 166.3, 155.6, 148.3, 131.6, 130.5, 130.3, 129.7, 124.5, 117.0, 114.0, 78.9, 66.2, 64.3, 45.1, 33.5, 25.3, 21.7, 18.1, -4.8. $[\alpha]_D^{24}$= -19.1 (c 1.1, $CHCl_3$)

4-nitrophenyl prop-2-yn-1-ylcarbamate (17). To a solution of *p*-nitrophenyl chloroformate (1.5 g, 7.5 mmol, 1 eq.) in dry THF (40 ml) at -55°C was slowly added propargylamine (500 mg. 9 mmol, 1.2 eq.), the reaction was stirred at -55°C for 45 min. The reaction was filtered through celite and washed with THF (3x20 ml). The solvent was evaporated and the crude dissolved in Hex/EtOAc 2:1 kept at room temperature for 1 h, and after stored at -10°C to crystalize compound **17**. The compound was filtered to yield a white solid (1.23 g, 73%).^{1}H NMR (300 MHz, $CDCl_3$) δ 8.25 (d, J = 9.1 Hz, 2H), 7.33 (d, J = 9.2 Hz, 2H), 5.38 (s, 1H), 4.09 (dd, J = 5.6, 2.5 Hz, 2H), 2.33 (t, J = 2.5 Hz, 1H). ^{13}C NMR (75 MHz, $CHCl_3$) δ 155.7, 152.9, 145.0, 125.3, 122.1, 78.2, 72.6, 31.2.

(2R,3R)-3-((R)-1-((tert-butyldimethylsilyl)oxy)ethyl)-2-(4-(13-((2,4-dinitrophenyl)amino)-4-oxo-8,11-dioxa-3,5-diazatridecyl)phenoxy)-4-oxo-N-(prop-2-yn-1-yl)azetidine-1-carboxamide (18i). To a solution of compound **17** (360 mg, 1.63 mmol, 1 eq.) in dry DCM (3 ml), was added DMAP (400 mg, 3.27 mmol, 2 eq.) and the mixture was stirred for 30 minutes. A solution of compound **16i** (1.15 g, 1.63 mmol, 1 eq.) and triethylamine (228 μl, 1.63 mmol, 1 eq.) was slowly added and the reaction was stirred at room temperature overnight. The solvent was evaporated and the crude was purified by flash chromatography using silica-gel and as eluent a gradient of hexane/ethyl acetate (from 100% to 0% of hexane) to yield a yellow oil (424 mg, 33%). ^{1}H NMR (300 MHz, $CDCl_3$) δ 9.03 (d, J = 2.7 Hz, 1H), 8.87 (s, 1H), 8.22 (ddd, J = 9.5, 2.7, 0.7 Hz, 1H), 7.13 – 7.04 (m, 4H), 6.87 (d, J = 9.5 Hz, 1H), 6.71 (t, J = 5.6 Hz, 1H), 5.91 (d, J = 1.7 Hz, 1H), 5.09 (s, 1H), 5.00 (s, 1H), 4.22 (qd, J = 6.2, 1.7 Hz, 1H), 4.10 – 3.87 (m, 2H), 3.76 (t, J =

5.2 Hz, 2H), 3.70 – 3.47 (m, 8H), 3.47 – 3.31 (m, 4H), 3.26 (dd, *J* = 3.0, 1.5 Hz, 1H), 2.73 (t, *J* = 7.0 Hz, 2H), 2.18 (t, *J* = 2.5 Hz, 1H), 1.18 (d, *J* = 6.2 Hz, 3H), 0.76 (s, 9H), -0.00 (s, 3H), -0.04 (s, 3H). ^{13}C NMR (75 MHz, $CDCl_3$) δ 162.3, 158.3, 133.9, 130.3, 130.3, 124.5, 122.1, 116.3, 114.2, 78.7, 71.3, 70.9, 70.2, 67.7, 65.9, 64.4, 64.1, 43.0, 41.6, 40.2, 35.7, 29.7, 25.8, 22.5, 19.8, 1.7, -4.3, -12.6. $[\alpha]_D^{24}$= -42.3 (c 1.2, CHCl3)

(2R,3R)-3-((R)-1-((tert-butyldimethylsilyl)oxy)ethyl)-2-(4-(2-(3-(3,5-dinitrophenyl)ureido)ethyl)phenoxy)-4-oxo-N-(prop-2-yn-1-yl)azetidine-1-carboxamide (18ii). To a solution of compound **17** (51 mg, 0.23 mmol, 1 eq.) in dry DCM (0.5 ml) DMAP was added (65 mg, 0.50 mmol, 2 eq.) and the mixture was stirred for 30 minutes. A solution of compound **16ii** (160 mg, 0,23 mmol, 1 eq.) and triethylamine (32 *μ*l, 0,23 mmol, 1 eq.) was slowly added and the reaction was stirred at room temperature overnight. The solvent was evaporated and the crude was purified by flash chromatography using silica-gel and as eluent a gradient of hexane/ethyl acetate (from 100% to 0% of hexane) to yield a yellow solid (53 mg, 29%). ^{1}H NMR (300 MHz, $CDCl_3$) δ 8.50 (t, *J* = 2.0 Hz, 2H), 7.15 – 7.04 (m, 3H), 6.96 (s, 1H), 6.92 – 6.82 (m, 1H), 6.82 – 6.70 (m, 2H), 5.56 (d, *J* = 1.2 Hz, 1H), 4.15 (dd, *J* = 6.3, 3.4 Hz, 1H), 3.62 – 3.34 (m, 2H), 3.13 (dd, *J* = 3.4, 1.2 Hz, 1H), 2.93 – 2.59 (m, 3H), 2.02 – 1.94 (m, 1H), 1.73 (s, 2H), 1.20 (t, *J* = 7.2 Hz, 3H), 0.78 (s, 9H), 0.00 (s, 3H), -0.04 (s, 3H). ^{13}C NMR (75 MHz, $CDCl_3$) δ 168.3, 152.9, 155.3, 152.7, 146.7, 135.8, 131.6, 129.9, 120.7, 115.5, 113.1, 79.9, 78.7, 71.3, 69.4, 56.0, 41.4, 34.6, 30.5, 0.7, -5.3, -7.3. $[\alpha]_D^{24}$= -31.6 (c 0.9, $CHCl_3$)

(2R,3R)-3-((R)-1-((tert-butyldimethylsilyl)oxy)ethyl)-2-(4-(2-((2,4-dinitrophenyl)amino)ethyl)phenoxy)-4-oxo-N-(prop-2-yn-1-yl)azetidine-1-carboxamide (18iii). To a solution of compound **17** (500 mg, 0.27 mmol, 1 eq.) in dry DCM (5 ml) DMAP was added (554 mg, 4.54 mmol, 2 eq.) and the mixture was stirred for 30 minutes. A solution of compound **16iii** (1.21 g, 2.27 mmol, 1 eq.) and triethylamine (317 *μ*l, 2.27 mmol, 1 eq.) was slowly added and the reaction was stirred at room temperature 48h. The solvent was evaporated and the crude was purified by flash chromatography using silica-gel and as eluent a gradient of hexane/ethyl acetate (from 100% to 0% of hexane) to yield a yellow solid (635 mg, 45%). ^{1}H NMR (300 MHz, $CDCl_3$) δ 9.10 (d, *J* = 2.7 Hz, 1H), 8.56 (s, 1H), 8.23 (dd, *J* = 9.5, 2.7 Hz, 1H), 7.30 – 7.08 (m, 4H), 6.86 (d, *J* = 9.5 Hz, 1H), 6.74 (t, *J* = 5.6 Hz, 1H), 6.01 (d, *J* = 1.5 Hz, 1H), 4.27 (qd, *J* = 6.3, 3.0 Hz, 1H), 4.07 – 3.93 (m, 2H), 3.68 – 3.54 (m, 2H), 3.34 (dd, *J* = 3.0, 1.5 Hz, 1H), 3.00 (t, *J* = 7.0 Hz,

2H), 2.21 (t, *J* = 2.5 Hz, 1H), 1.23 (d, *J* = 6.3 Hz, 3H), 0.79 (s, 9H), 0.04 (s, 3H), 0.00 (s, 3H). ^{13}C NMR (75 MHz, Acetone-*d*$_6$) δ 167.7, 156.9, 150.2, 149.5, 136.6, 134.5, 131.1, 130.9, 130.1, 129.5, 124.6, 119.9, 116.0, 83.2, 81.1, 72.7, 66.4, 65.2, 62.9, 45.7, 40.0, 38.3, 34.9, 26.3, 22.7, -2.3, -6.7. $[\alpha]_D^{24}$= -36.1 (c 1.1, $CHCl_3$)

(2R,3R)-2-(4-(13-((2,4-dinitrophenyl)amino)-4-oxo-8,11-dioxa-3,5-diazatridecyl)phenoxy)-3-((R)-1-hydroxyethyl)-4-oxo-N-(prop-2-yn-1-yl)azetidine-1-carboxamide (19i). To a solution of **18i** (60 mg, 0.08 mmol, 1 eq.) in ACN (3 ml), was added a solution of HCl 1M (230 μl, 0.228 mmol, 3 eq.), and the reaction was stirred at room temperature for 6h. The solvent was evaporated and the crude was purified by flash chromatography using silica-gel and as eluent a gradient of hexane/ethyl acetate (from 100% to 0% of hexane) to yield a yellow oil (39 mg, 73%). ^{1}H NMR (300 MHz, $CDCl_3$) δ 9.07 (d, *J* = 2.7 Hz, 1H), 8.89 (s, 1H), 8.26 (dd, *J* = 9.5, 2.7 Hz, 1H), 7.19 – 7.05 (m, 4H), 6.91 (d, *J* = 9.6 Hz, 1H), 6.74 (t, *J* = 5.6 Hz, 1H), 5.96 (d, *J* = 1.6 Hz, 1H), 5.14 (s, 1H), 5.03 (s, 1H), 4.28 – 4.17 (m, 1H), 4.05 (dd, *J* = 5.6, 2.5 Hz, 2H), 3.80 (t, *J* = 5.2 Hz, 2H), 3.74 – 3.46 (m, 8H), 3.46 – 3.26 (m, 5H), 2.74 (t, *J* = 6.9 Hz, 2H), 2.25 (t, *J* = 2.5 Hz, 1H), 1.31 (d, *J* = 6.4 Hz, 3H). ^{13}C NMR (75 MHz, $CDCl_3$) δ 167.0, 158.6, 155.3, 149.4, 148.5, 136.4, 134.8, 130.7, 130.2, 130.1, 124.5, 118.4, 114.3, 82.7, 79.1, 72.1, 71.2, 70.9, 70.3, 68.0, 65.5, 63.9, 54.0, 43.2, 41.7, 40.4, 29.5. $[\alpha]_D^{24}$= -40.8 (c 1.3, $CHCl_3$)

(2R,3R)-2-(4-(2-(3-(3,5-dinitrophenyl)ureido)ethyl)phenoxy)-3-((R)-1-hydroxyethyl)-4-oxo-N-(prop-2-yn-1-yl)azetidine-1-carboxamide (19ii). To a solution of **18ii** (36 mg, 0.06 mmol, 1 eq.) in ACN (3 ml), was added a solution of HCl 1M (83 μl, 0.08 mmol, 1.5 eq.), and the reaction was stirred at room temperature for 3h. The solvent was evaporated and the crude was purified by flash chromatography using silica-gel and as eluent a gradient of hexane/ethyl acetate (from 100% to 0% of hexane) to yield an orange oil (26 mg, 81%). ^{1}H NMR (300 MHz, $CDCl_3$) δ 9.16 (s, 1H), 9.08 – 8.97 (m, 2H), 7.72 (d, *J* = 1.2 Hz, 1H), 7.58 – 7.40 (m, 4H), 6.52 (d, *J* = 1.5 Hz, 1H), 6.09 (s, 1H), 5.73 (t, *J* = 5.6 Hz, 1H), 4.77 (s, 1H), 4.54 – 4.43 (m, 3H), 4.32 – 4.20 (m, 2H), 4.01 (dd, *J* = 13.1, 6.3 Hz, 1H), 3.92 – 3.78 (m, 2H), 2.76 (t, *J* = 1.8 Hz, 1H), 1.81 (d, *J* = 6.2 Hz, 3H). ^{13}C NMR (75 MHz, $CDCl_3$) δ 169.7, 154.9, 155.9, 152.3, 147.7, 138.8, 132.1, 129.7, 117.7, 115.5, 111.6, 75.3, 73.2, 72.3, 64.3, 55.8, 41.9, 34.3, 30.1, 20.9. $[\alpha]_D^{24}$= -46.3 (c 1.0, $CHCl_3$)

(2R,3R)-2-(4-(2-((2,4-dinitrophenyl)amino)ethyl)phenoxy)-3-((R)-1-hydroxyethyl)-4-oxo-N-(prop-2-yn-1-yl)azetidine-1-carboxamide (19iii). To a solution of **18ii** (20 mg, 0.03 mmol, 1 eq.) in ACN (1.5 ml), was added a solution of HCl 1M (48 *µ*l, 0.05 mmol, 1.5 eq.), and the reaction was stirred at room temperature for 3h. The solvent was evaporated and the crude was purified by flash chromatography using silica-gel and as eluent a gradient of hexane/ethyl acetate (from 100% to 0% of hexane) to yield an orange oil (14 mg, 85%). ^{1}H NMR (300 MHz, $CDCl_3$) *δ* 9.11 (d, *J* = 2.7 Hz, 1H), 8.56 (s, 1H), 8.25 (ddd, *J* = 9.6, 2.7, 0.7 Hz, 1H), 7.32 – 7.13 (m, 4H), 6.87 (d, *J* = 9.6 Hz, 1H), 6.73 (s, 1H), 6.03 (d, *J* = 1.6 Hz, 1H), 4.35 – 4.23 (m, 1H), 4.13 – 3.98 (m, 2H), 3.64 (td, *J* = 7.0, 5.2 Hz, 2H), 3.39 (dd, *J* = 5.1, 1.6 Hz, 1H), 3.03 (t, *J* = 6.9 Hz, 2H), 2.26 (t, *J* = 2.6 Hz, 1H), 1.91 (d, *J* = 4.7 Hz, 1H), 1.34 (d, *J* = 6.4 Hz, 3H). ^{13}C NMR (75 MHz, $CDCl_3$) *δ* 166.8, 162.4, 156.8, 156.0, 149.4, 148.3, 133.6, 132.4, 130.5, 130.0, 124.4, 118.8, 114.0, 82.2, 72.2, 65.4, 63.8, 45.0, 34.4, 33.4, 29.6, 22.0. HRMS (ESI) m/z calcd. for $C_{45}H_{54}BF_2N_{11}O_{12}$ $[M+H]^+$: 990.409 found: 990.412. $[\alpha]_D^{24}$= -35.6 (c 1.1, $CHCl_3$)

(2R,3R)-N-((1-(15-(5,5-difluoro-7,9-dimethyl-5H-4λ4,5λ4-dipyrrolo[1,2-c:2',1'-f][1,3,2]diazaborinin-3-yl)-13-oxo-3,6,9-trioxa-12-azapentadecyl)-1H-1,2,3-triazol-4-yl)methyl)-2-(4-(13-((2,4-dinitrophenyl)amino)-4-oxo-8,11-dioxa-3,5-diazatridecyl)phenoxy)-3-((R)-1-hydroxyethyl)-4-oxoazetidine-1-carboxamide (20). A solution of compound **19i** (29 mg, 0.04 mmol, 1 eq.), copper (II) sulphate (0.2 M aqueous solution) (96 *µ*l, 0.02mmol, 0.48 eq.) and sodium ascorbate (0.2 M aqueous solution) (96 *µ*l, 0.02 mmol, 0.48 eq.) in MeOH (1 ml) was stirred for 10 min. Then, was added compound **9** (30 mg, 0.06 mmol, 1.5 eq.) and the reaction was stirred at room temperature for 2h. The solvent was evaporated, and the residue was purified by flash chromatography using silica-gel and as eluent a gradient of dichloromethane/methanol (from 100% to 90% of dichloromethane) to yield an orange oil (31 mg, 64%). ^{1}H NMR (300 MHz, $CDCl_3$) *δ* 9.02 (d, *J* = 2.7 Hz, 1H), 8.83 (t, *J* = 4.9 Hz, 1H), 8.19 (dd, *J* = 9.6, 2.7 Hz, 1H), 7.71 (s, 1H), 7.15 – 6.99 (m, 4H), 6.89 (d, *J* = 9.6 Hz, 1H), 6.83 (d, *J* = 4.0 Hz, 1H), 6.58 (t, *J* = 5.4 Hz, 1H), 6.23 (d, *J* = 4.0 Hz, 1H), 6.08 (s, 1H), 5.99 (d, *J* = 1.5 Hz, 1H), 5.37 (t, *J* = 5.6 Hz, 1H), 5.20 (t, *J* = 5.8 Hz, 1H), 4.59 – 4.35 (m, 4H), 4.18 (p, *J* = 6.2 Hz, 1H), 3.86 – 3.72 (m, 4H), 3.72 – 3.25 (m, 27H), 3.19 (t, *J* = 7.6 Hz, 2H), 2.71 (t, *J* = 6.7 Hz, 2H), 2.61 (s, 1H), 2.51 (d, *J* = 8.1 Hz, 5H), 2.21 (s, 3H), 1.25 (d, *J* = 7.1 Hz, 3H). ^{13}C NMR (101 MHz, Acetone-*d$_6$*) *δ* 172.4, 167.5, 160.3, 159.2, 156.3, 150.5, 149.6, 145.1, 144.9, 135.5, 130.7, 130.6, 129.6, 125.6, 124.4, 124.1,

121.0, 119.0, 117.7, 116.1, 83.3, 71.5, 71.1, 71.0, 71.0, 71.0, 70.9, 70.5, 70.1, 69.3, 66.2, 64.1, 55.5, 50.7, 43.9, 43.6, 42.3, 40.7, 40.0, 36.5, 35.9, 35.2, 25.1, 22.3, 14.9, 11.2. HRMS (ESI) m/z calcd. for $C_{52}H_{68}BF_2N_{13}O_{15}$ $[M+H]^+$: 1164.5100 found: 1164.5160. $[\alpha]_D^{24}$= -27.5 (c 1.1, $CHCl_3$)

(2R,3R)-N-((1-(15-(5,5-difluoro-7,9-dimethyl-5H-4λ4,5λ4-dipyrrolo[1,2-c:2',1'-f][1,3,2]diazaborinin-3-yl)-13-oxo-3,6,9-trioxa-12-azapentadecyl)-1H-1,2,3-triazol-4-yl)methyl)-2-(4-(2-(3-(3,5-dinitrophenyl)ureido)ethyl)phenoxy)-3-((R)-1-hydroxyethyl)-4-oxoazetidine-1-carboxamide (21). A solution of compound **19ii** (24 mg, 0.04 mmol, 1 eq.), copper (II) sulphate (0.2 M aqueous solution) (105 *μ*l, 0.02mmol, 0.48 eq.) and sodium ascorbate (0.2 M aqueous solution) (105 *μ*l, 0.02 mmol, 0.48 eq.) in MeOH (1 ml) was stirred for 10 min. Then, was added compound **9** (30 mg, 0.06 mmol, 1.5 eq.) and the reaction was stirred at room temperature for 2h. The solvent was evaporated, and the residue was purified by flash chromatography using silica-gel and as eluent a gradient of dichloromethane/methanol (from 100% to 90% of dichloromethane) to yield an orange oil (41 mg, 91%). ^{1}H NMR (300 MHz, Acetone-d_6) δ 9.17 (s, 1H), 8.63 (d, J = 2.1 Hz, 2H), 8.25 (t, J = 2.1 Hz, 1H), 7.79 (s, 1H), 7.30 (s, 1H), 7.26 (d, J = 7.1 Hz, 1H), 7.18 – 7.05 (m, 5H), 6.86 (d, J = 4.1 Hz, 1H), 6.25 – 6.18 (m, 1H), 6.16 (s, 1H), 6.08 (s, 1H), 5.95 (d, J = 1.5 Hz, 1H), 4.49 – 4.31 (m, 4H), 4.15 – 4.00 (m, 1H), 3.73 (dd, J = 5.6, 4.6 Hz, 2H), 3.52 – 3.29 (m, 14H), 3.29 – 3.16 (m, 3H), 3.12 – 2.97 (m, 2H), 2.50 – 2.40 (m, 3H), 2.34 (s, 3H), 2.13 (s, 3H), 1.21 – 1.11 (m, 3H). ^{13}C NMR (101 MHz, Acetone-d_6) δ 171.8, 166.6, 155.7, 154.7, 149.7, 148.7, 144.3, 143.5, 134.1, 129.8, 129.0, 128.7, 124.7, 123.3, 120.1, 118.1, 116.9, 116.9, 109.7, 82.4, 77.9, 70.2, 70.0, 69.5, 69.2, 65.5, 64.1, 63.3, 49.8, 41.0, 39.2, 35.0, 34.3, 24.3, 21.4, 16.9, 13.9, 12.9, 10.3. HRMS (ESI) m/z calcd. for $C_{46}H_{55}BF_2N_{12}O_{13}$ $[M+H]^+$: 1033.4159 found: 1033.4189. $[\alpha]_D^{24}$= -64.1 (c 1.2, $CHCl_3$)

(2R,3R)-N-((1-(15-(5,5-difluoro-7,9-dimethyl-5H-4λ4,5λ4-dipyrrolo[1,2-c:2',1'-f][1,3,2]diazaborinin-3-yl)-13-oxo-3,6,9-trioxa-12-azapentadecyl)-1H-1,2,3-triazol-4-yl)methyl)-2-(4-(2-((2,4-dinitrophenyl)amino)ethyl)phenoxy)-3-((R)-1-hydroxyethyl)-4-oxoazetidine-1-carboxamide (22). A solution of compound **19iii** (18 mg, 0.04 mmol, 1 eq.), copper (II) sulphate (0.2 M aqueous solution) (90 *μ*l, 0.02mmol, 0.48 eq.) and sodium ascorbate (0.2 M aqueous solution) (90 *μ*l, 0.02 mmol, 0.48 eq.) in MeOH (1 ml) was stirred for 10 min. Then, was added compound 9 (15 mg, 0.03 mmol, 0.9 eq.) and the reaction was stirred at room temperature for 2h. The solvent was evaporated, and the residue was purified by

flash chromatography using silica-gel and as eluent a gradient of dichloromethane/methanol (from 100% to 90% of dichloromethane) to yield an orange oil (30 mg, 83%). ^{1}H NMR (300 MHz, $CHCl_3$) δ 9.08 (d, J = 2.6 Hz, 1H), 8.56 (d, J = 5.5 Hz, 1H), 8.21 (dd, J = 9.5, 2.7 Hz, 1H), 7.70 (s, 1H), 7.30 – 7.15 (m, 6H), 7.04 (d, J = 6.8 Hz, 2H), 6.91 – 6.81 (m, 2H), 6.36 (t, J = 5.4 Hz, 1H), 6.25 (d, J = 4.0 Hz, 1H), 6.15 – 6.00 (m, 2H), 4.61 (dd, J = 15.3, 6.3 Hz, 1H), 4.52 – 4.41 (m, 3H), 4.31 – 4.18 (m, 1H), 3.81 (t, J = 5.0 Hz, 2H), 3.68 – 3.43 (m, 10H), 3.43 – 3.31 (m, 4H), 3.23 (t, J = 7.6 Hz, 2H), 3.00 (t, J = 7.0 Hz, 2H), 2.61 – 2.48 (m, 5H), 2.23 (s, 3H), 1.30 (d, J = 6.4 Hz, 3H). ^{13}C NMR (75 MHz, Acetone-d_6) δ 176.6, 169.2, 158.9, 151.6, 144.6, 137.8, 133.5, 130.3, 130.3, 124.7, 123.8, 120.2, 118.8, 116.7, 115.4, 115.1, 114.7, 109.2, 109.0, 108.6, 81.3, 70.6, 70.5, 70.4, 69.9, 69.5, 65.7, 63.9, 63.5, 63.2, 50.2, 45.1, 39.4, 35.4, 34.7, 34.2, 24.4, 21.7, 17.8, 15.0, 12.1, 10.5. HRMS (ESI) m/z calcd. for $C_{45}H_{54}BF_2N_{11}O_{12}$ $[M+H]^+$: 990.4095 found: 990.4122. $[\alpha]_D^{24}$= -62.1 (c 1.1, $CHCl_3$)

NMR and HRMS spectra of qABPs

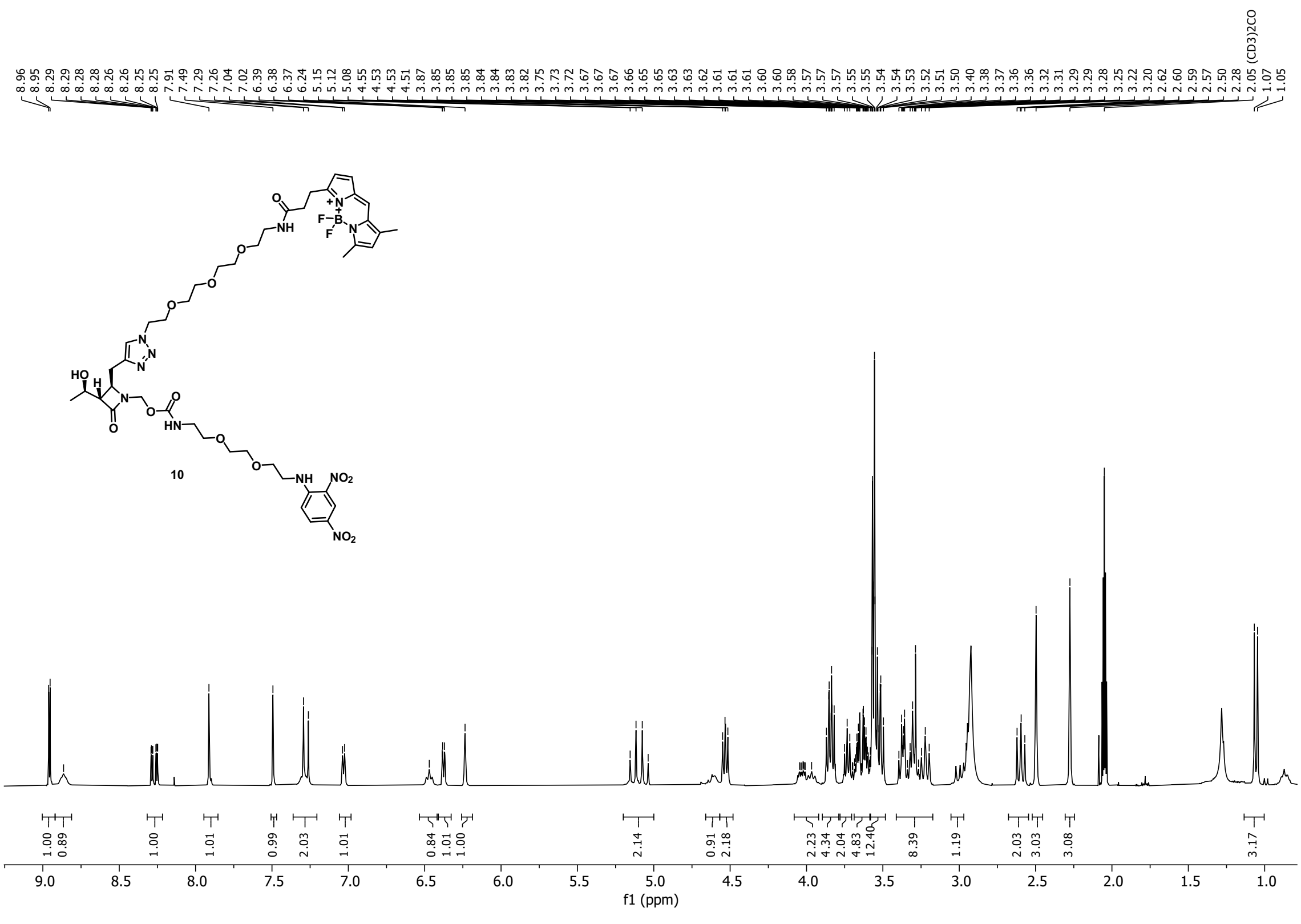

Figure S3. ^{1}H spectrum of qABP **10**.

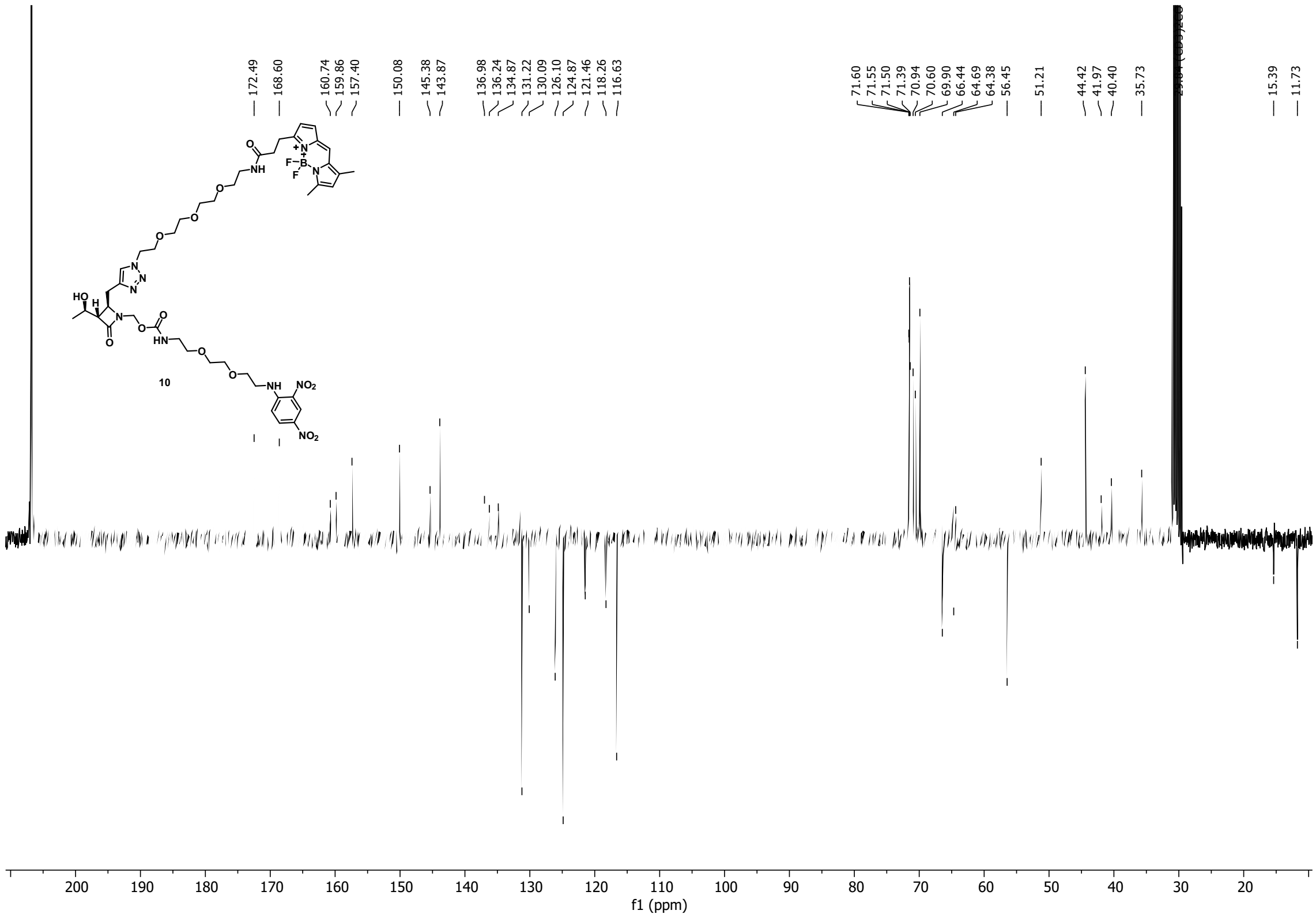

Figure S4. ^{13}C spectrum of qABP **10**.

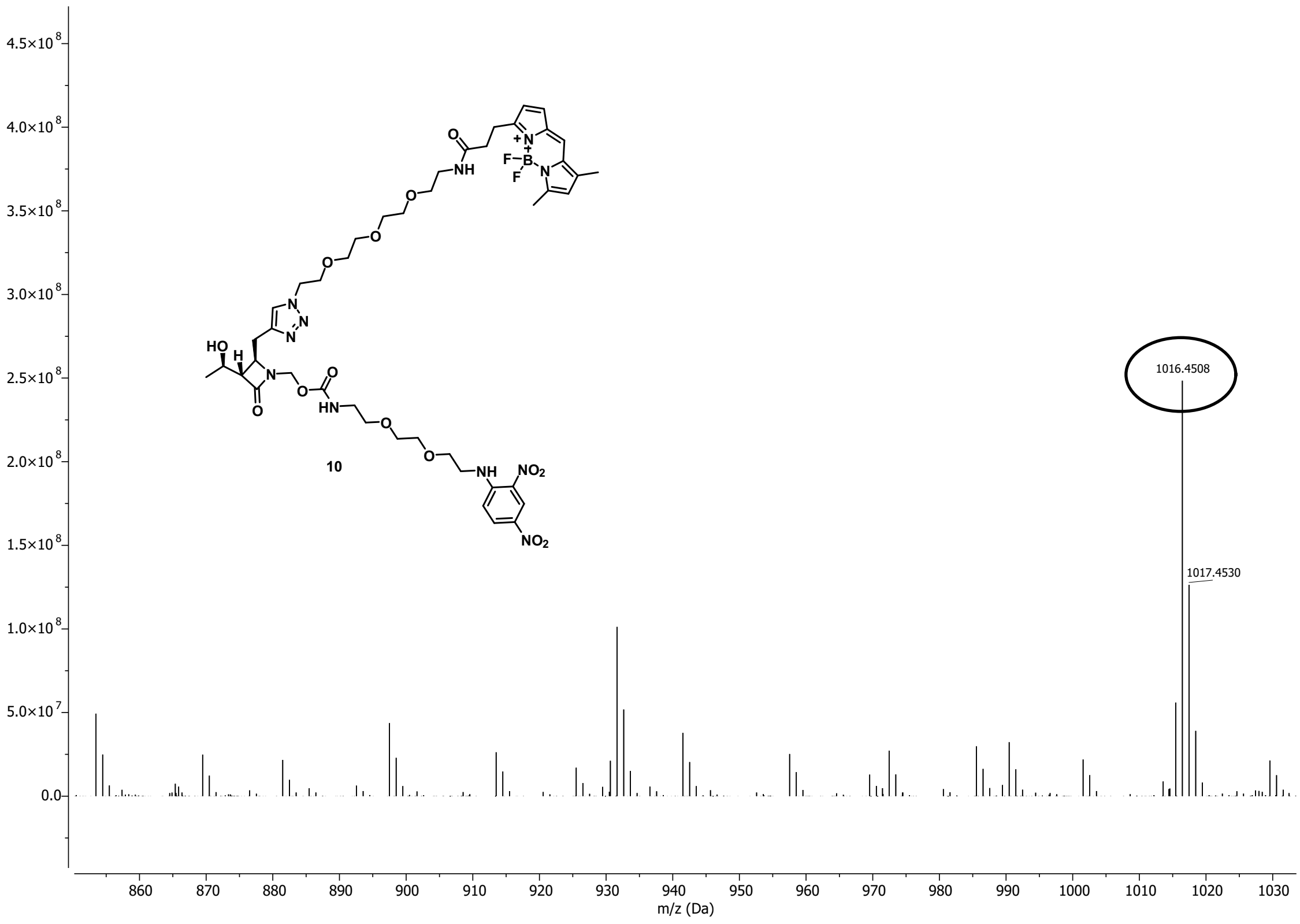

Figure S5. HRMS spectrum of qABP **10**.

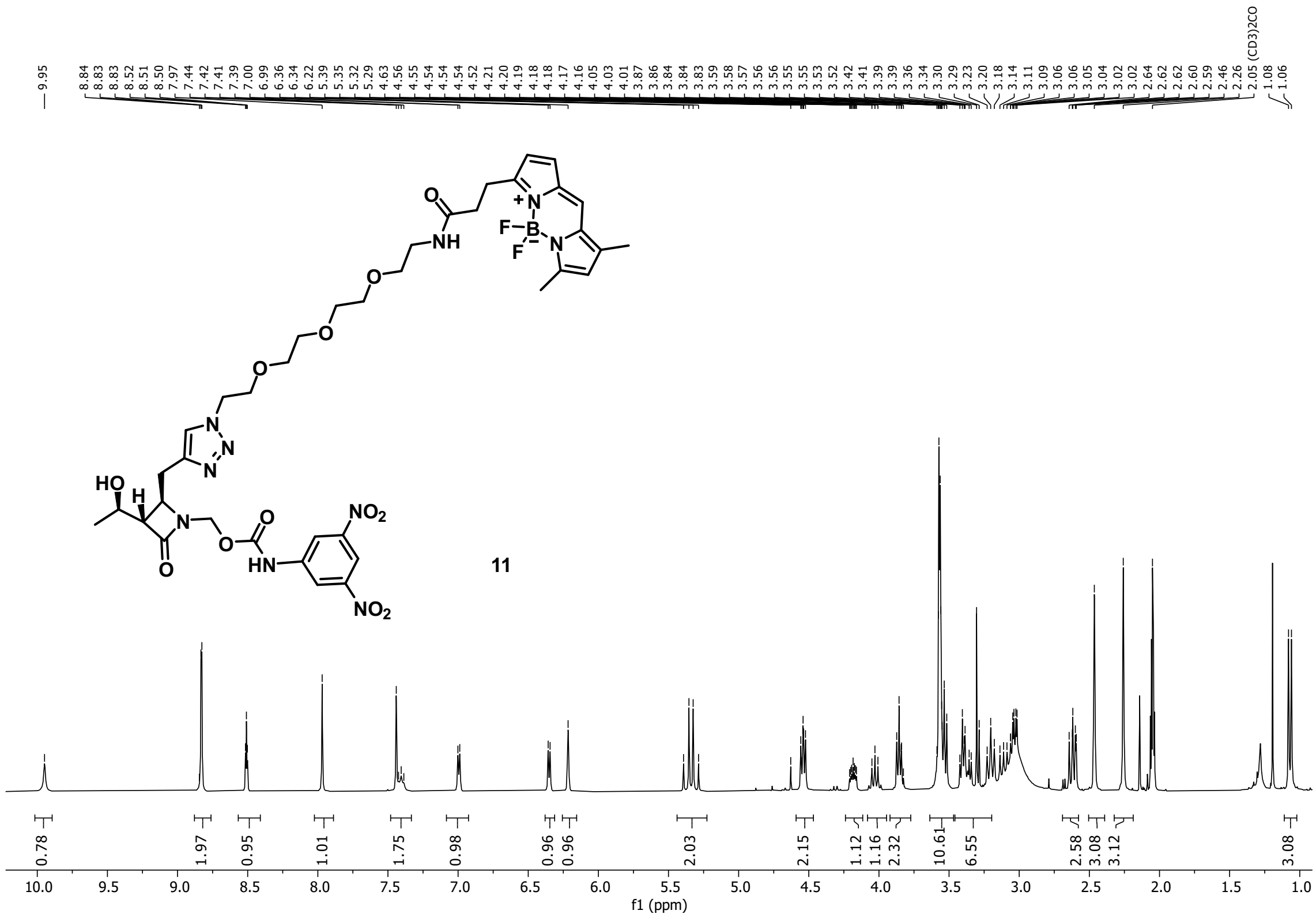

Figure S6. ^{1}H spectrum of qABP **11**.

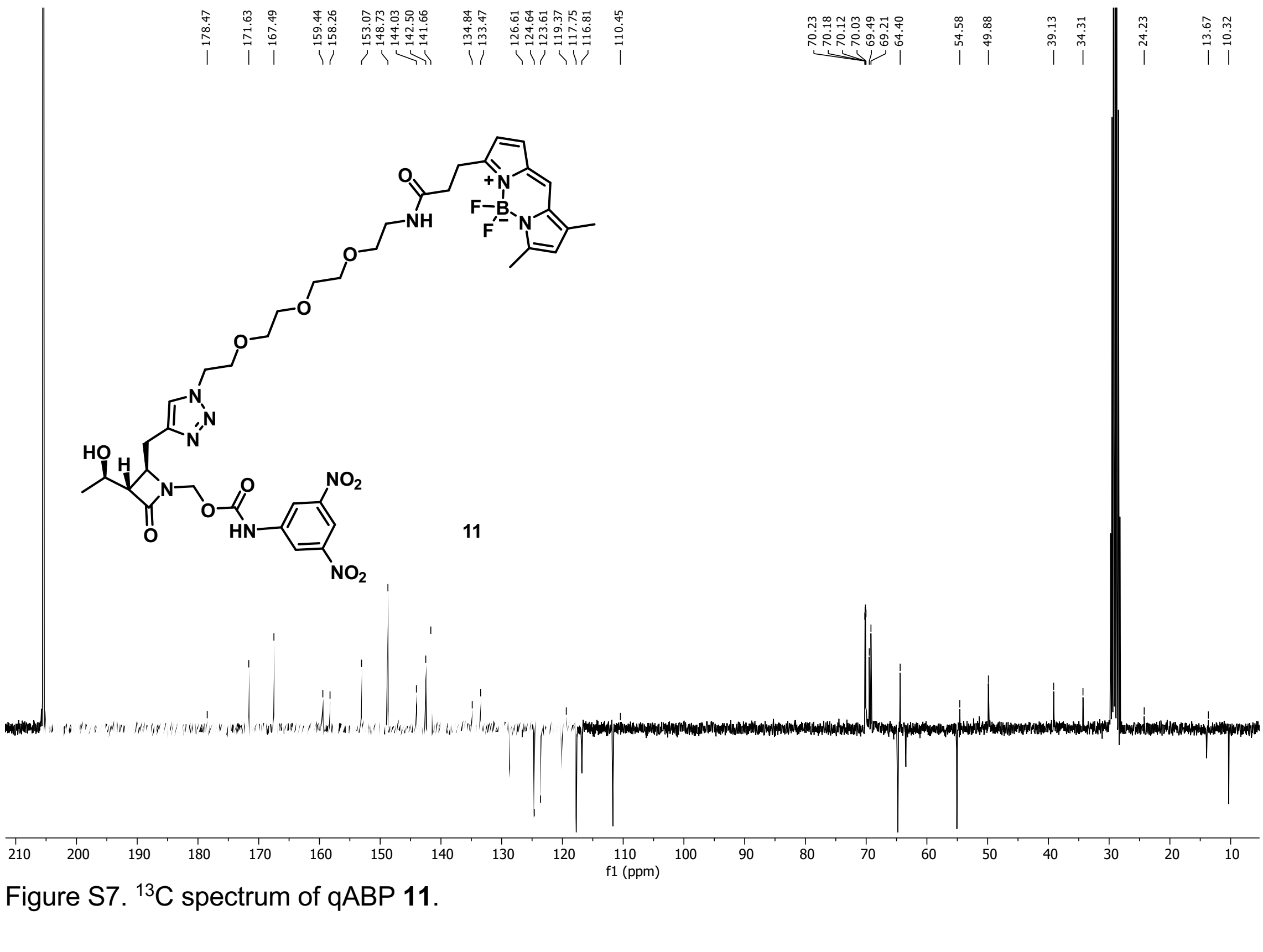

Figure S7. ^{13}C spectrum of qABP **11**.

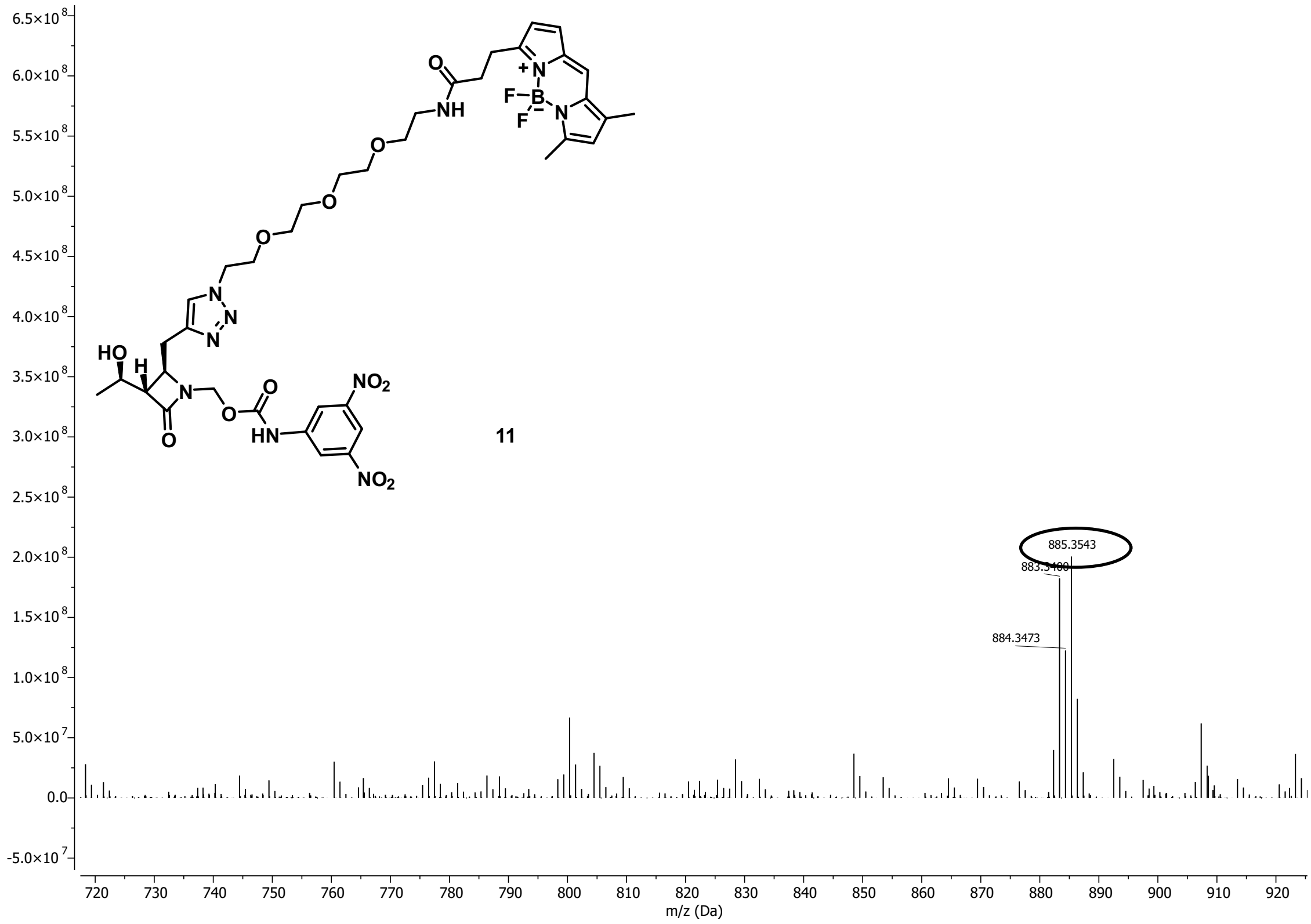

Figure S8. HRMS spectrum of qABP **11**.

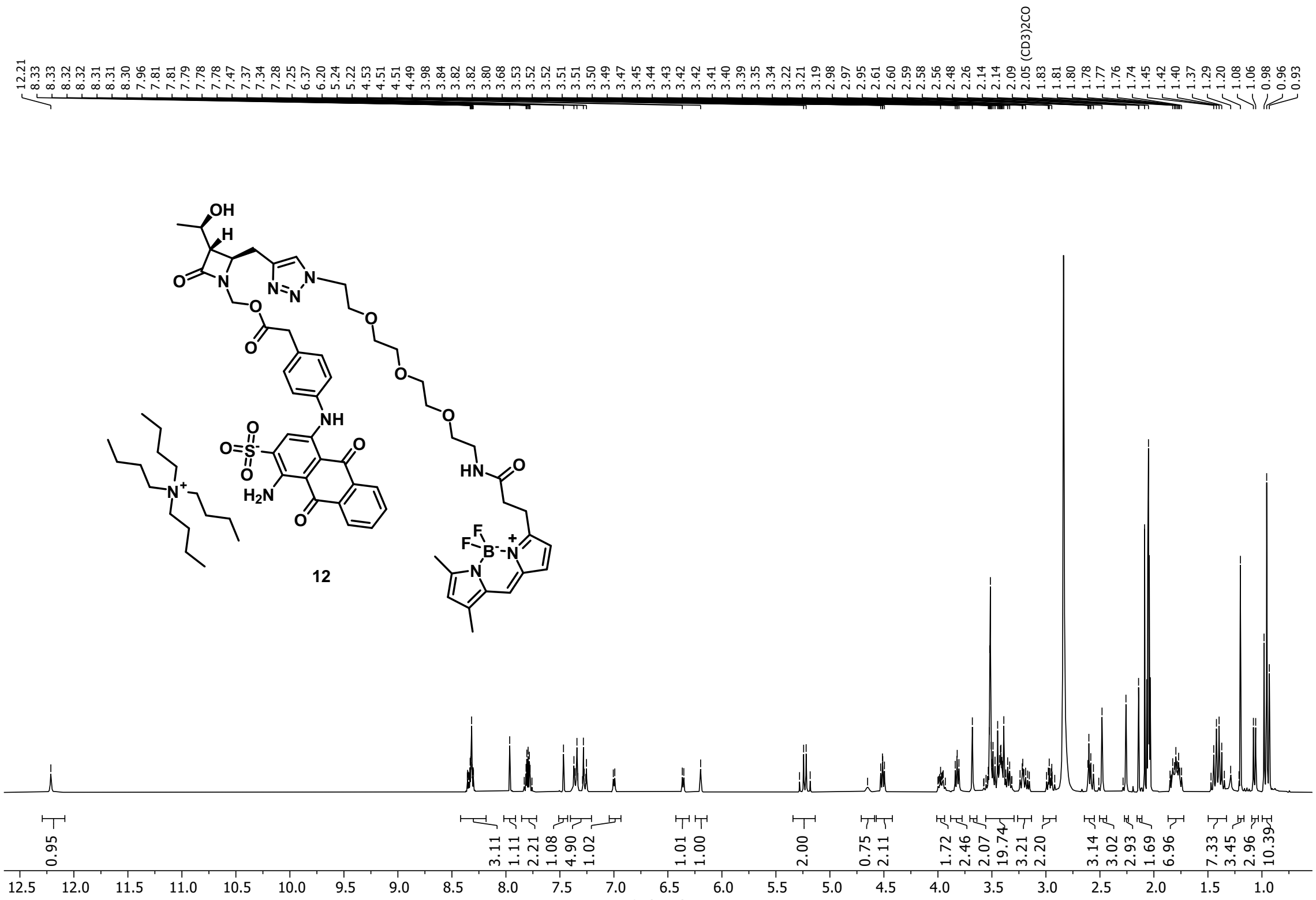

Figure S9. ^{1}H spectrum of qABP **12**.

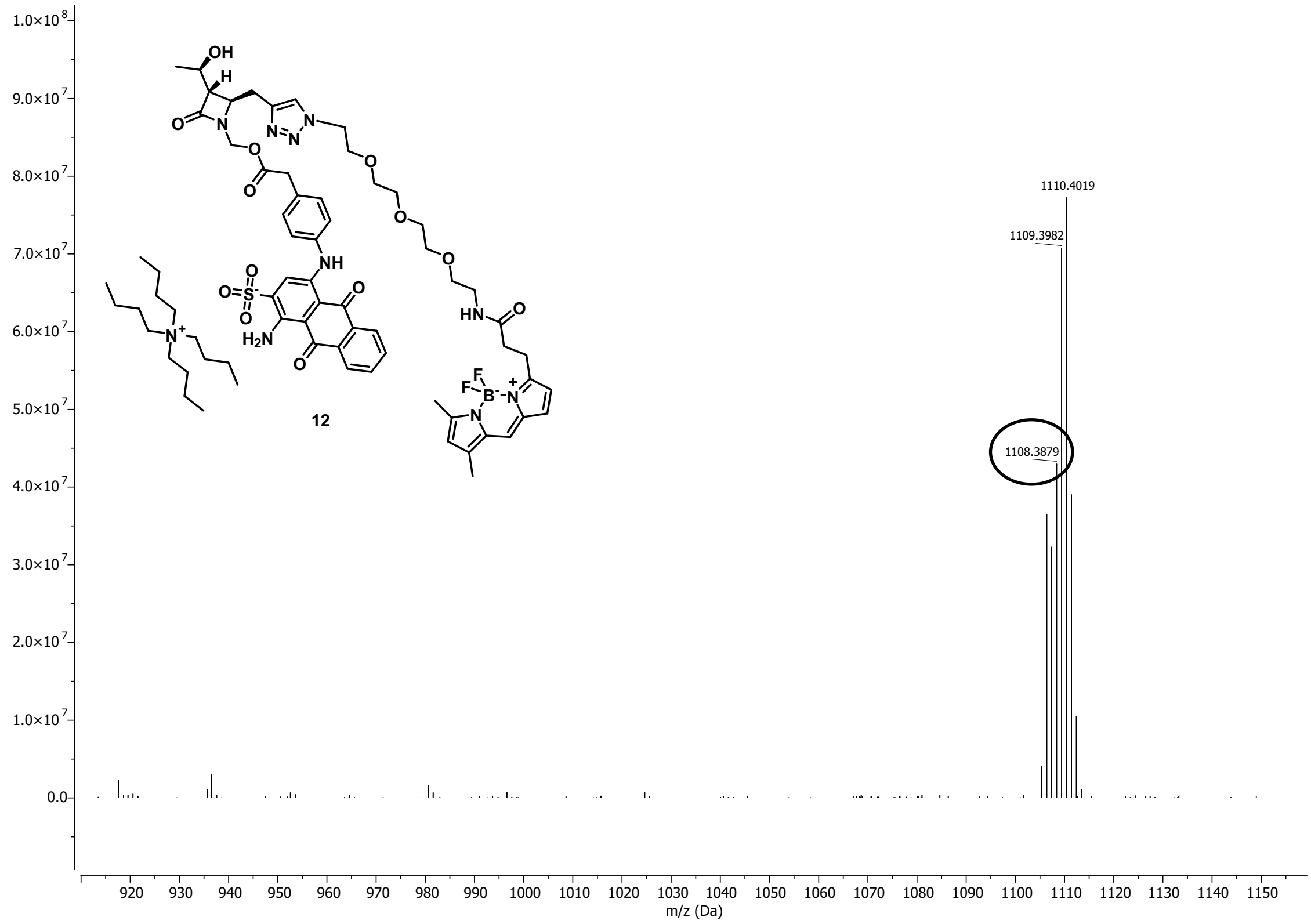

Figure S10. HRMS spectrum of qABP **12**.

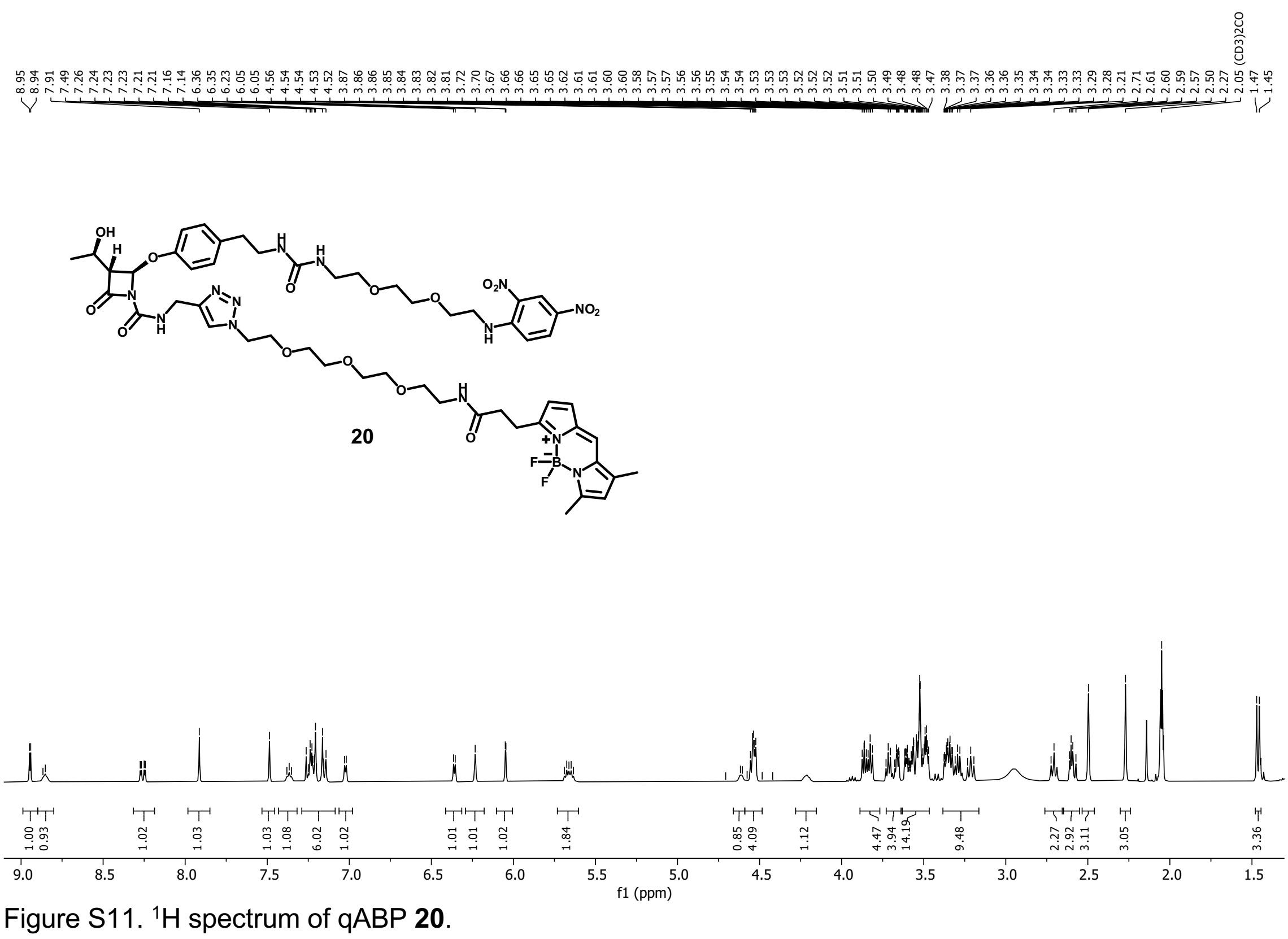

Figure S11. ^{1}H spectrum of qABP **20**.

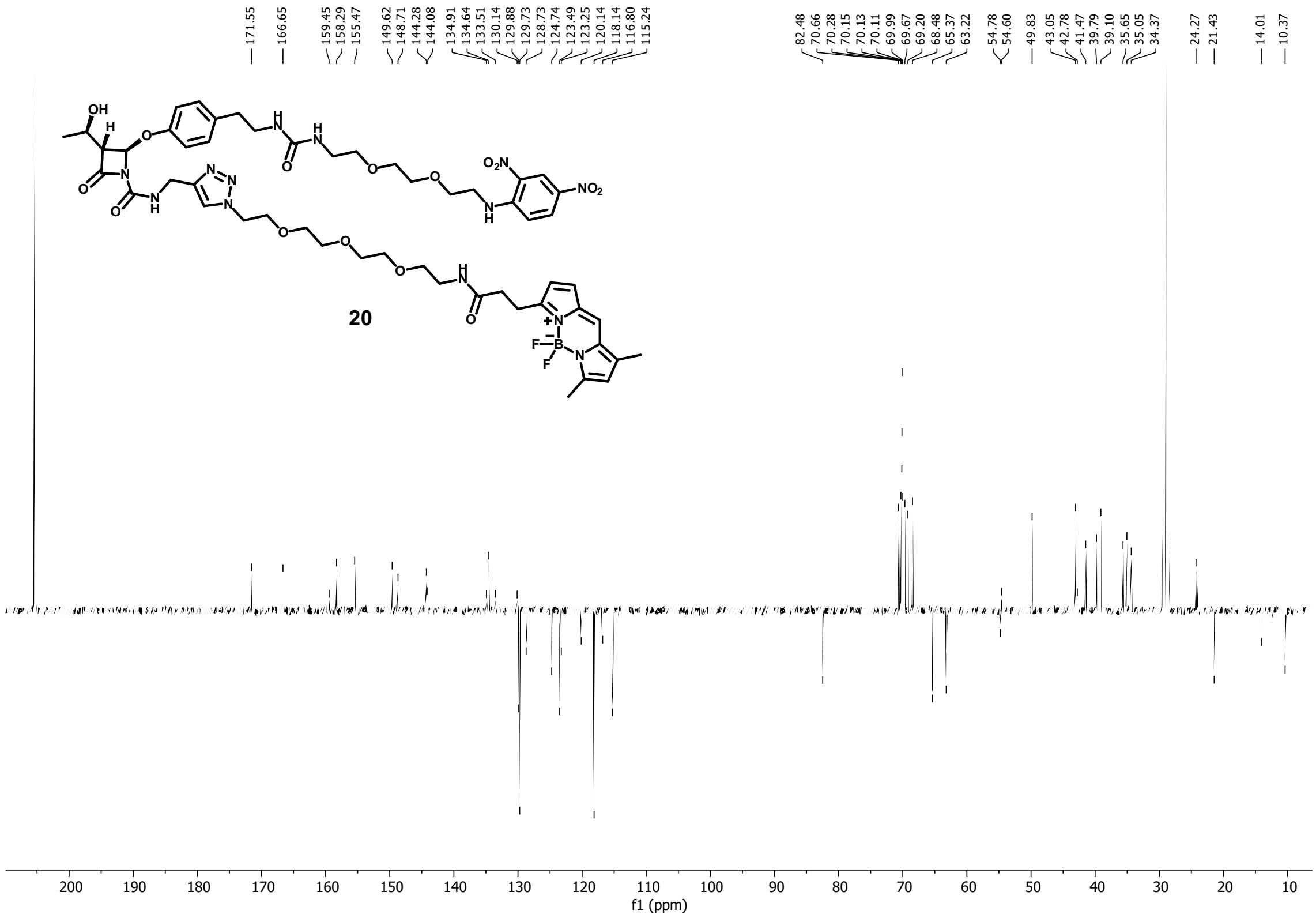

Figure S12. ^{13}C spectrum of qABP **20**.

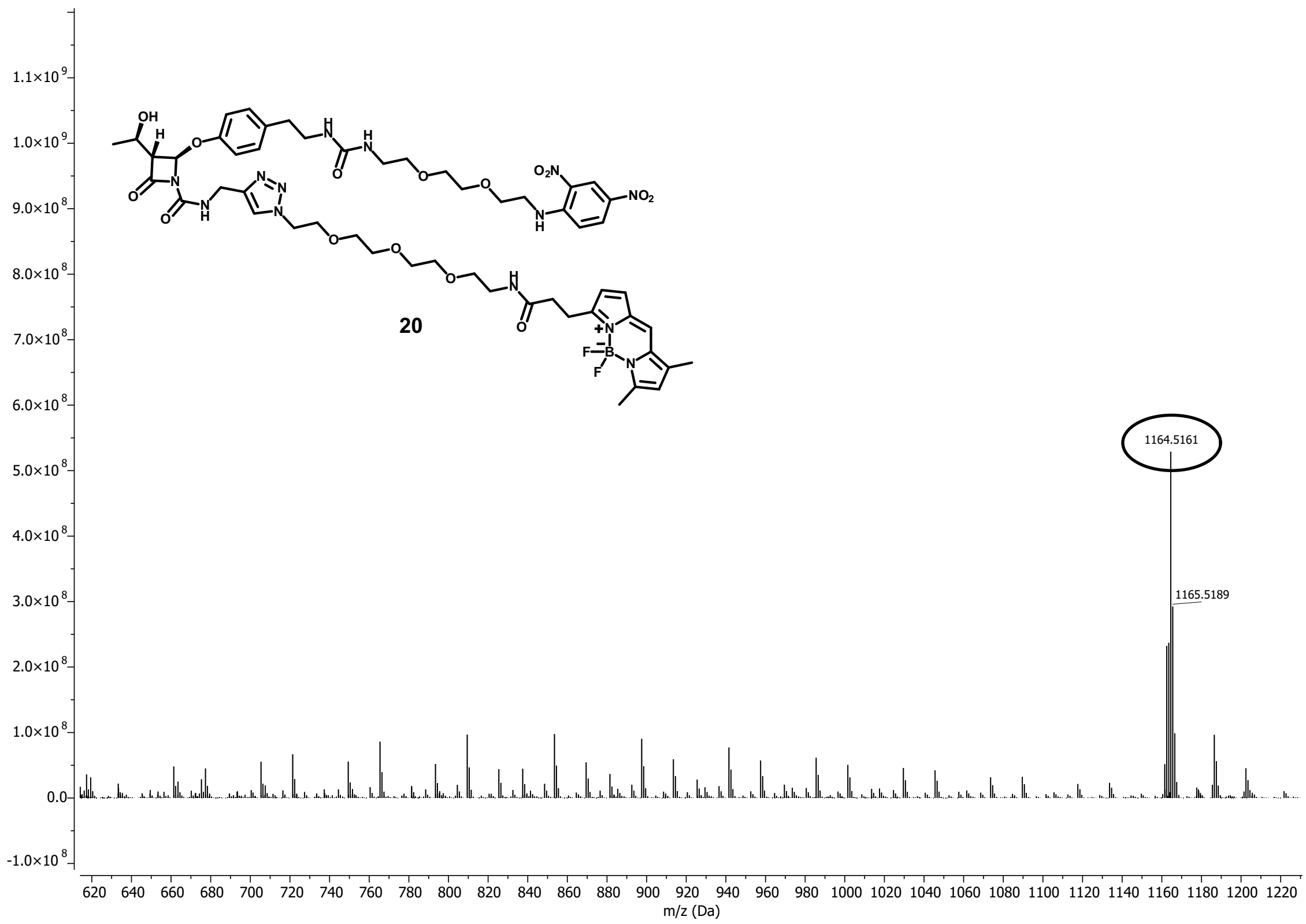

Figure S13. HRMS spectrum of qABP **20**.

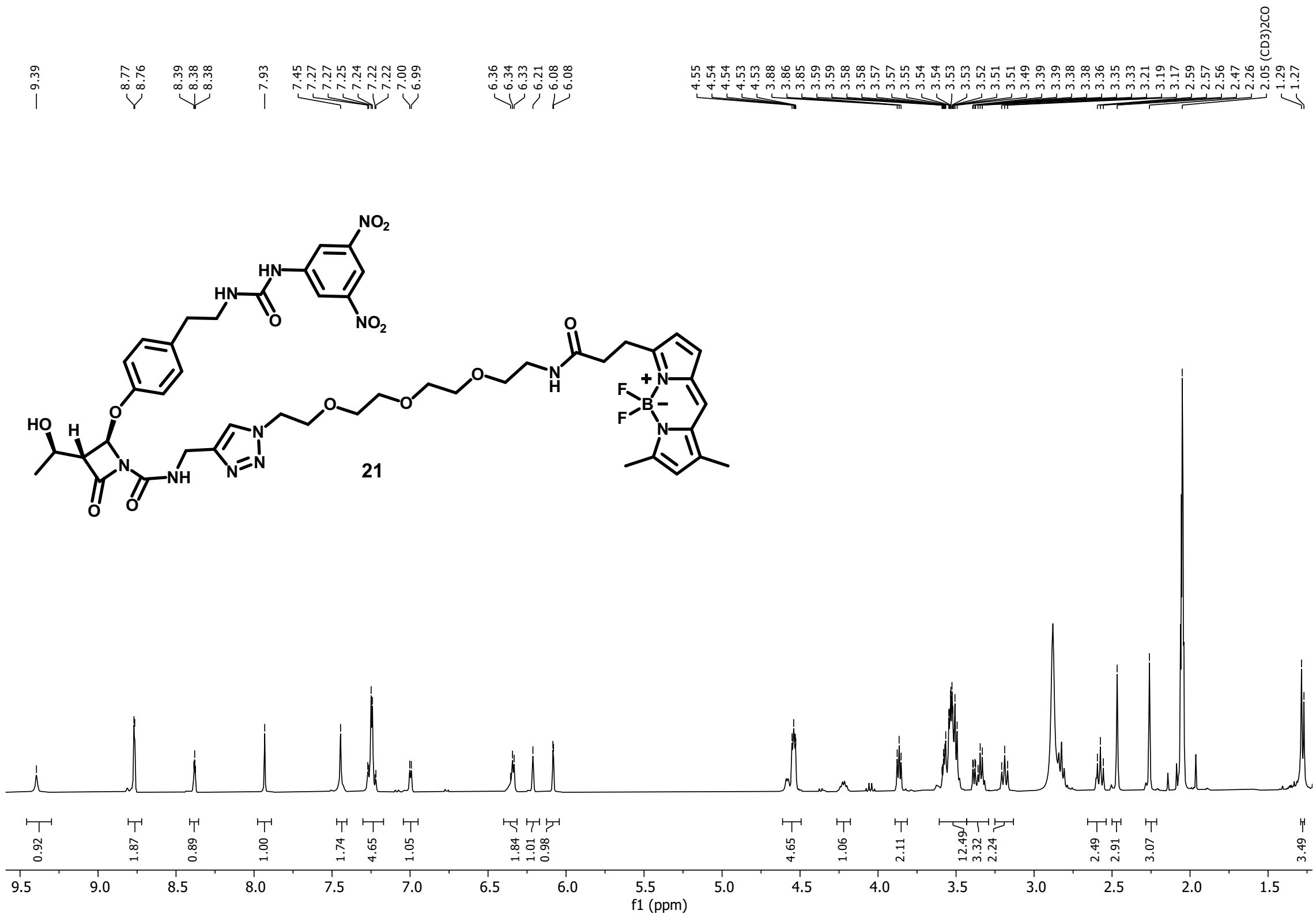

Figure S14. ^{1}H spectrum of qABP **21**.

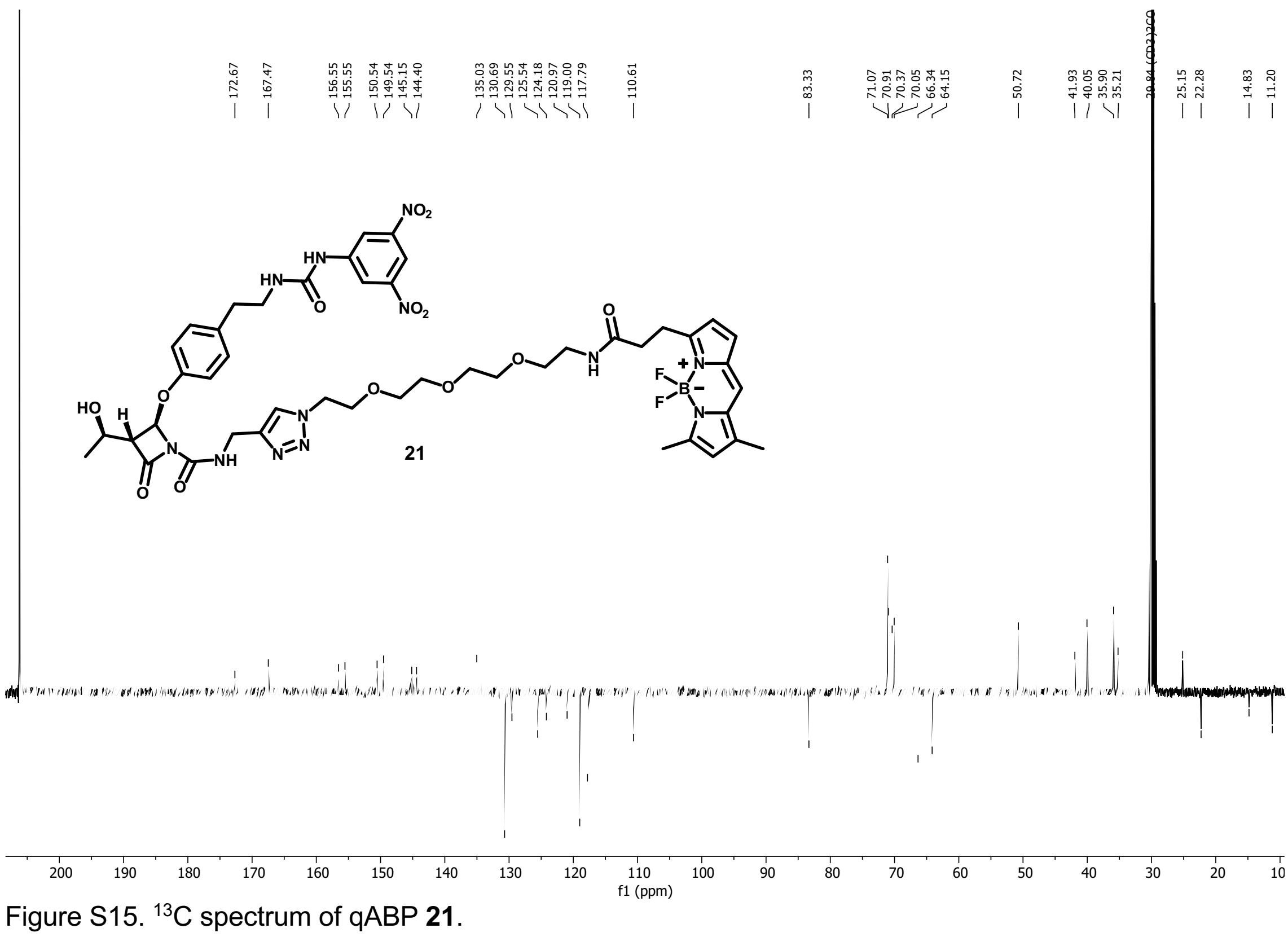

Figure S15. ^{13}C spectrum of qABP **21**.

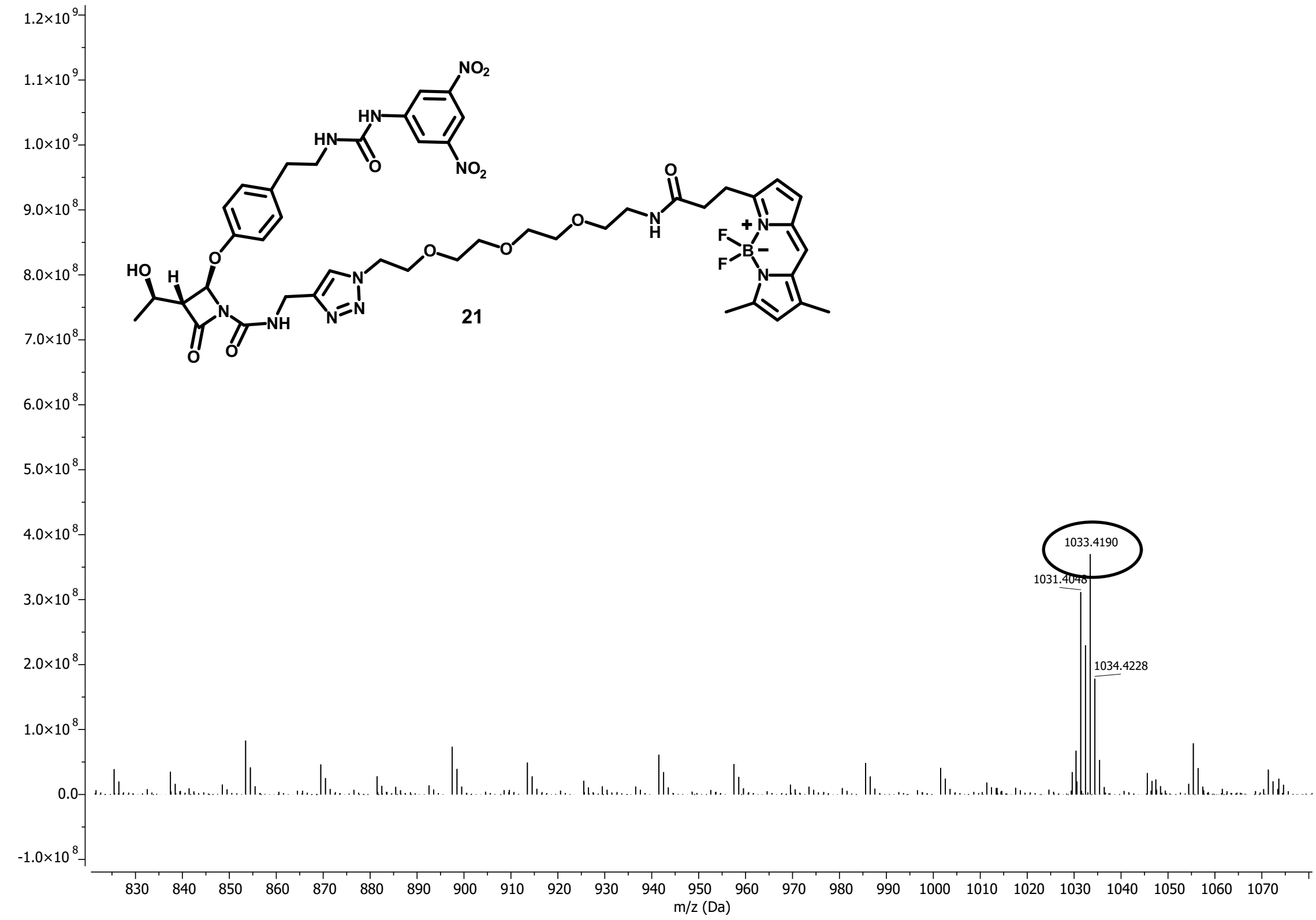

Figure S16. HRMS spectrum of qABP **21**.

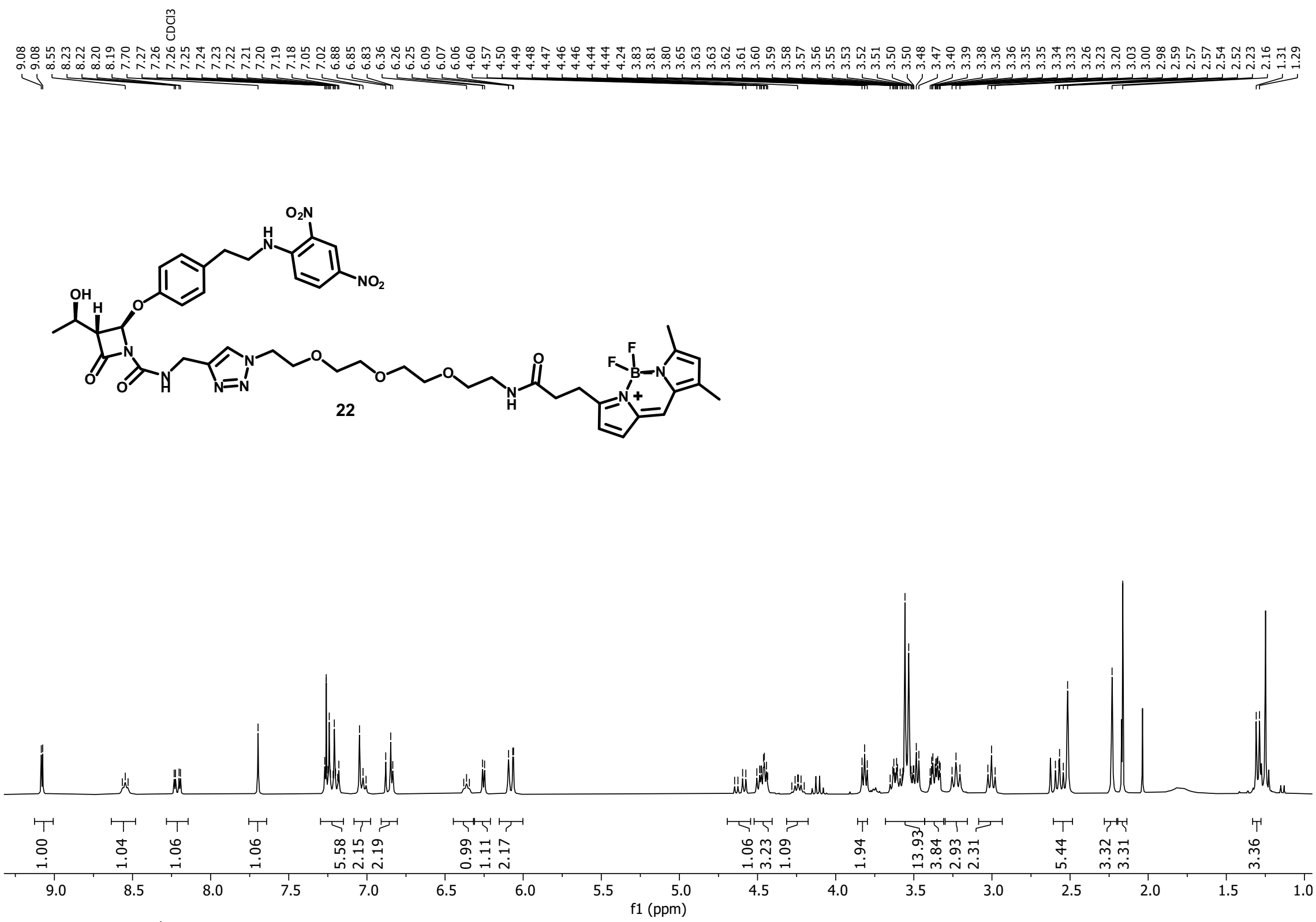

Figure S17. ^{1}H spectrum of qABP **22**.

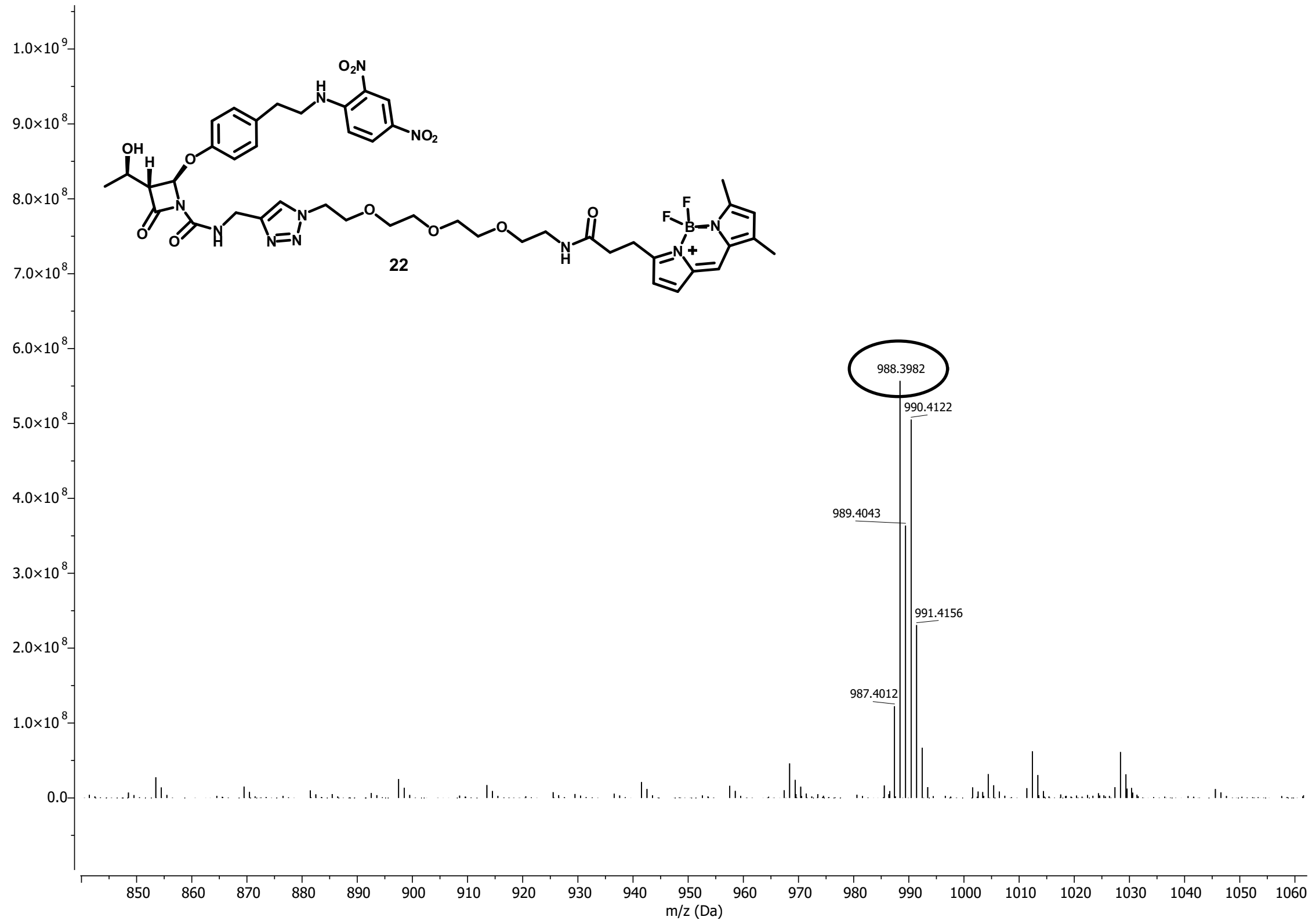

Figure S18. HRMS spectrum of qABP **22**.

Stability of qABPs in different pHs

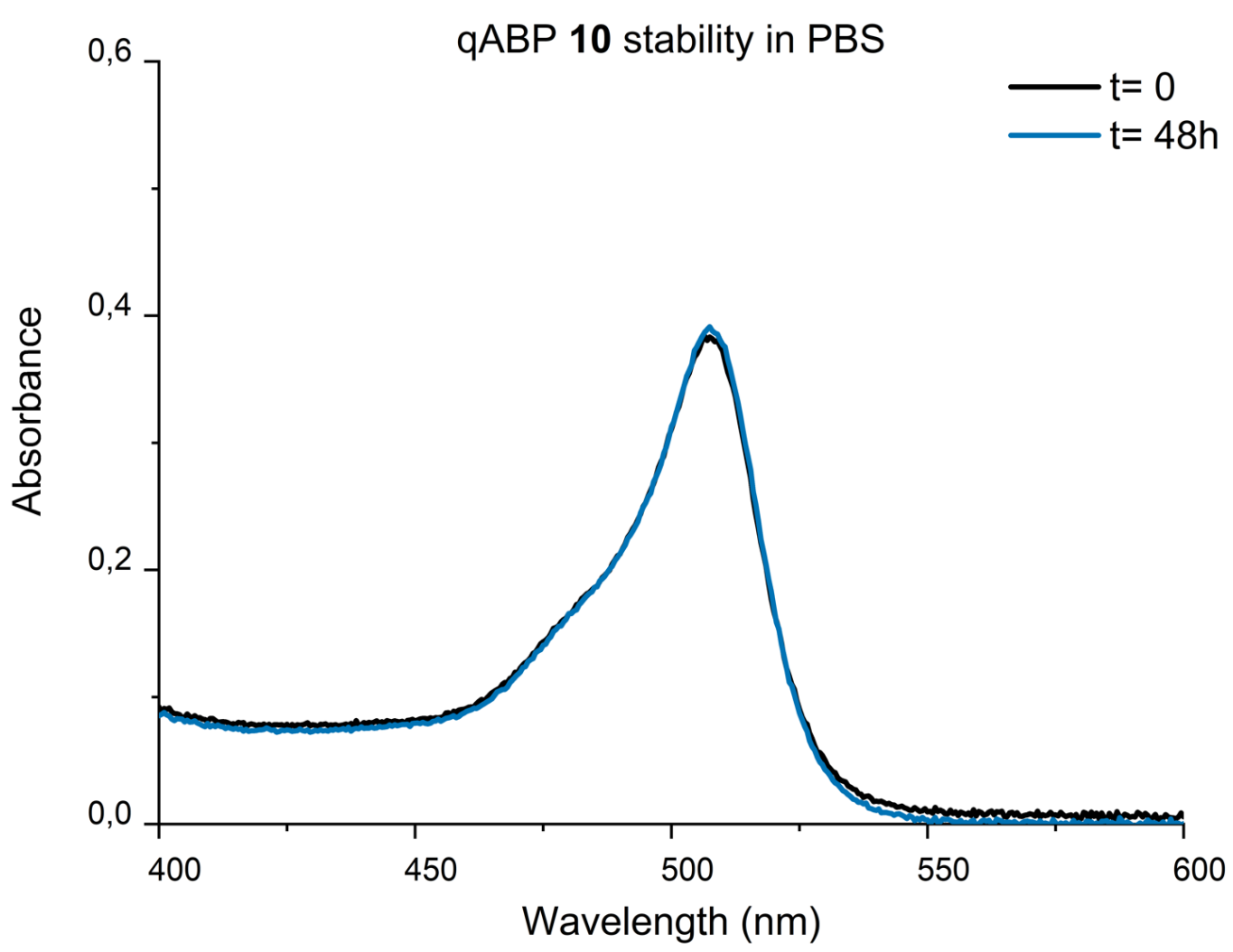

Figure S19. UV-spectra of qABP **10** (1µM in PBS with 0.07% of DMSO) measure at different time points, until 48 hours.

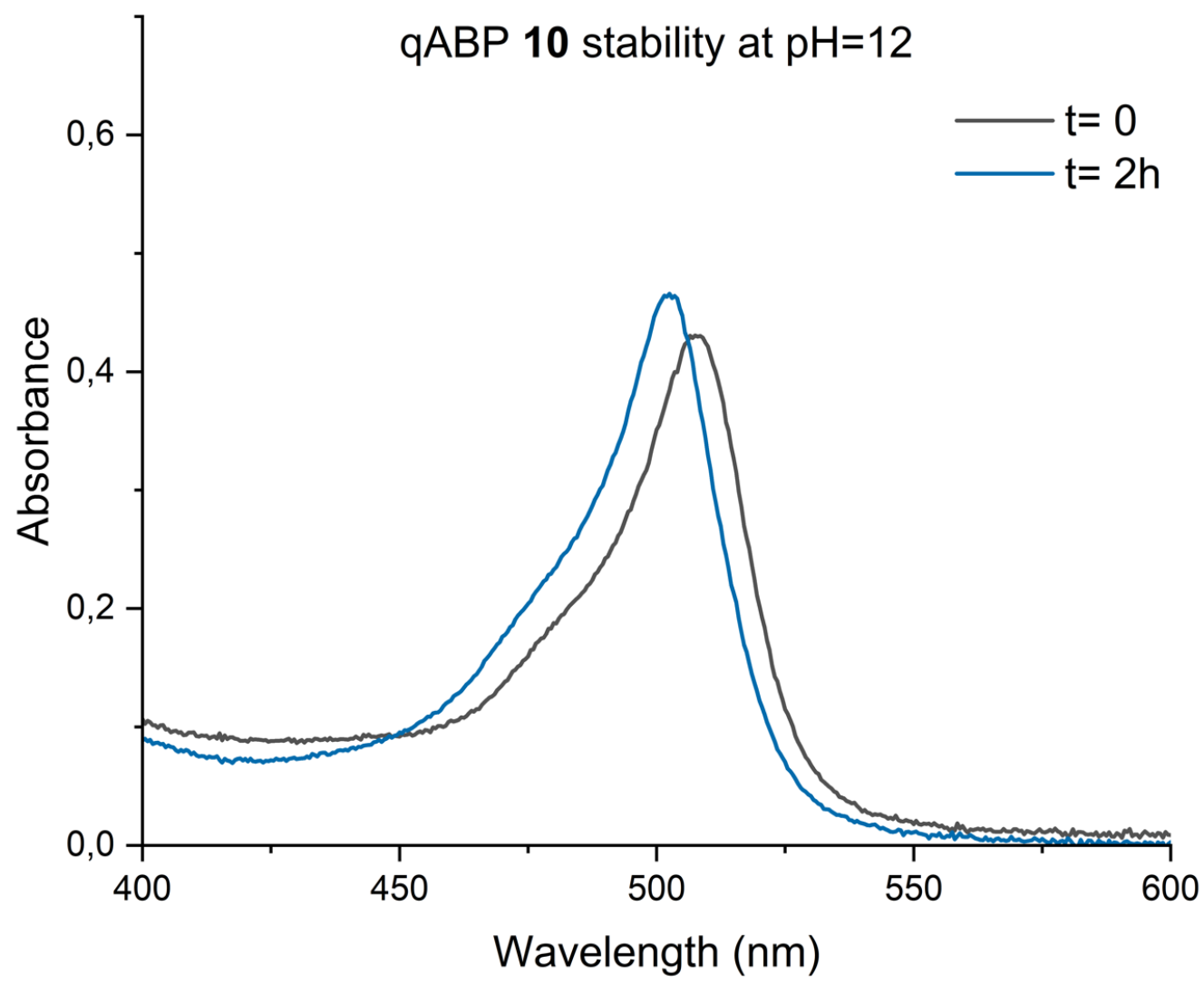

Figure S20. UV-spectra of qABP **10** (1μM at pH=12 with 0.07% of DMSO) measure at different time points, until 2 hours.

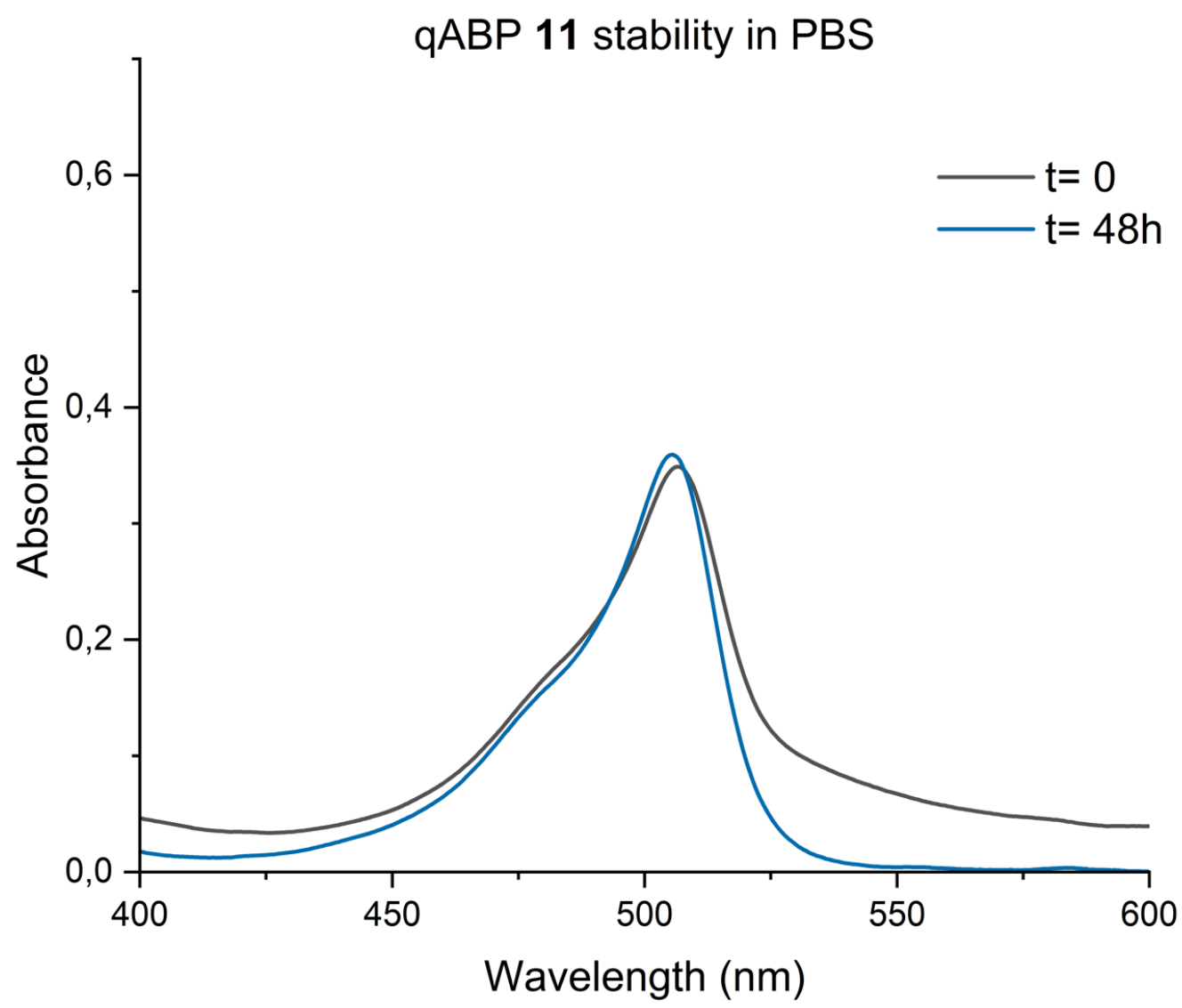

Figure S 21. UV-spectra of qABP **11** (1μM in PBS with 0.07% of DMSO) measure at different time points, until 48 hours.

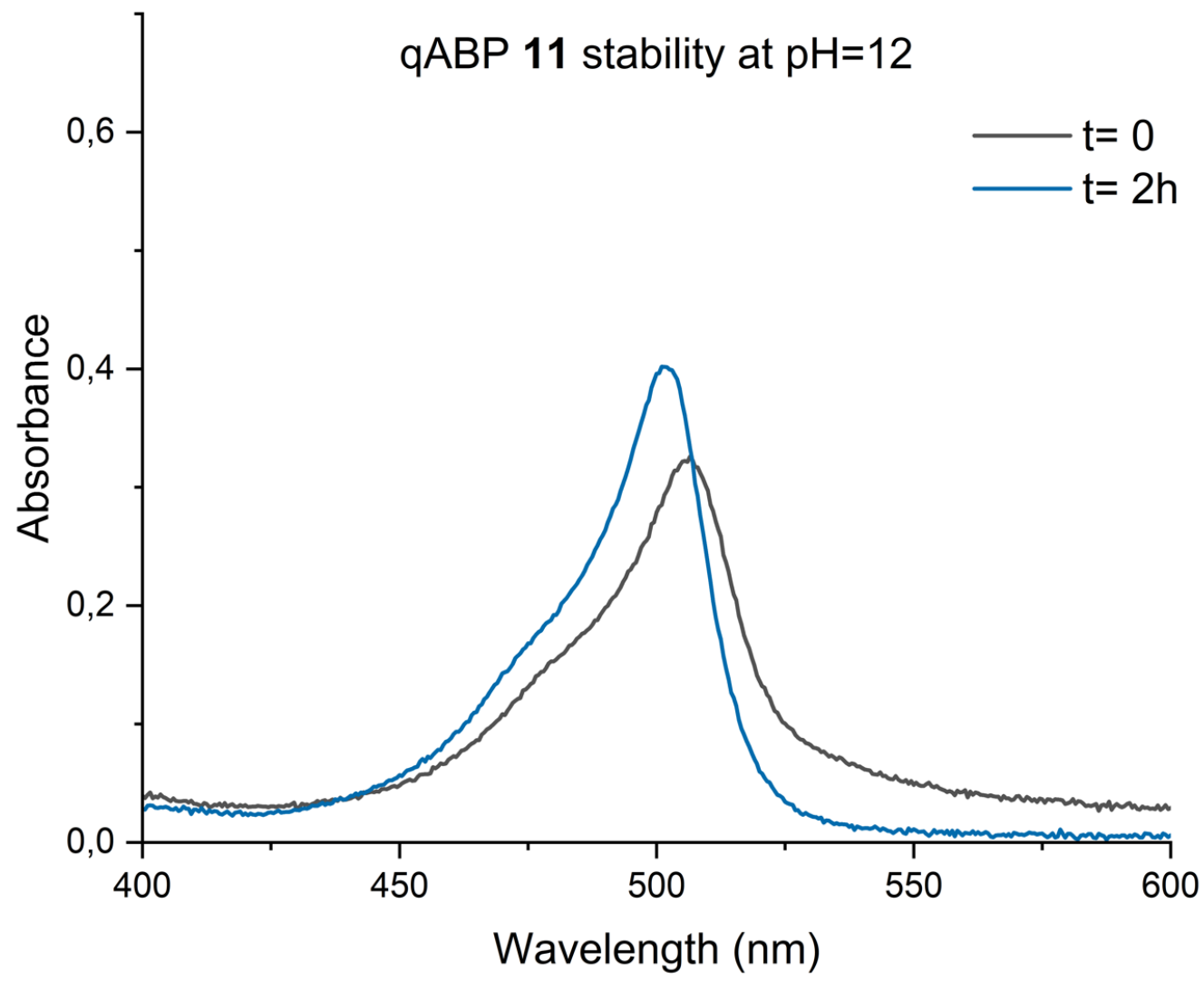

Figure S22.UV-spectra of qABP **11** (1µM at pH=12 with 0.07% of DMSO) measure at different time points, until 2 hours.

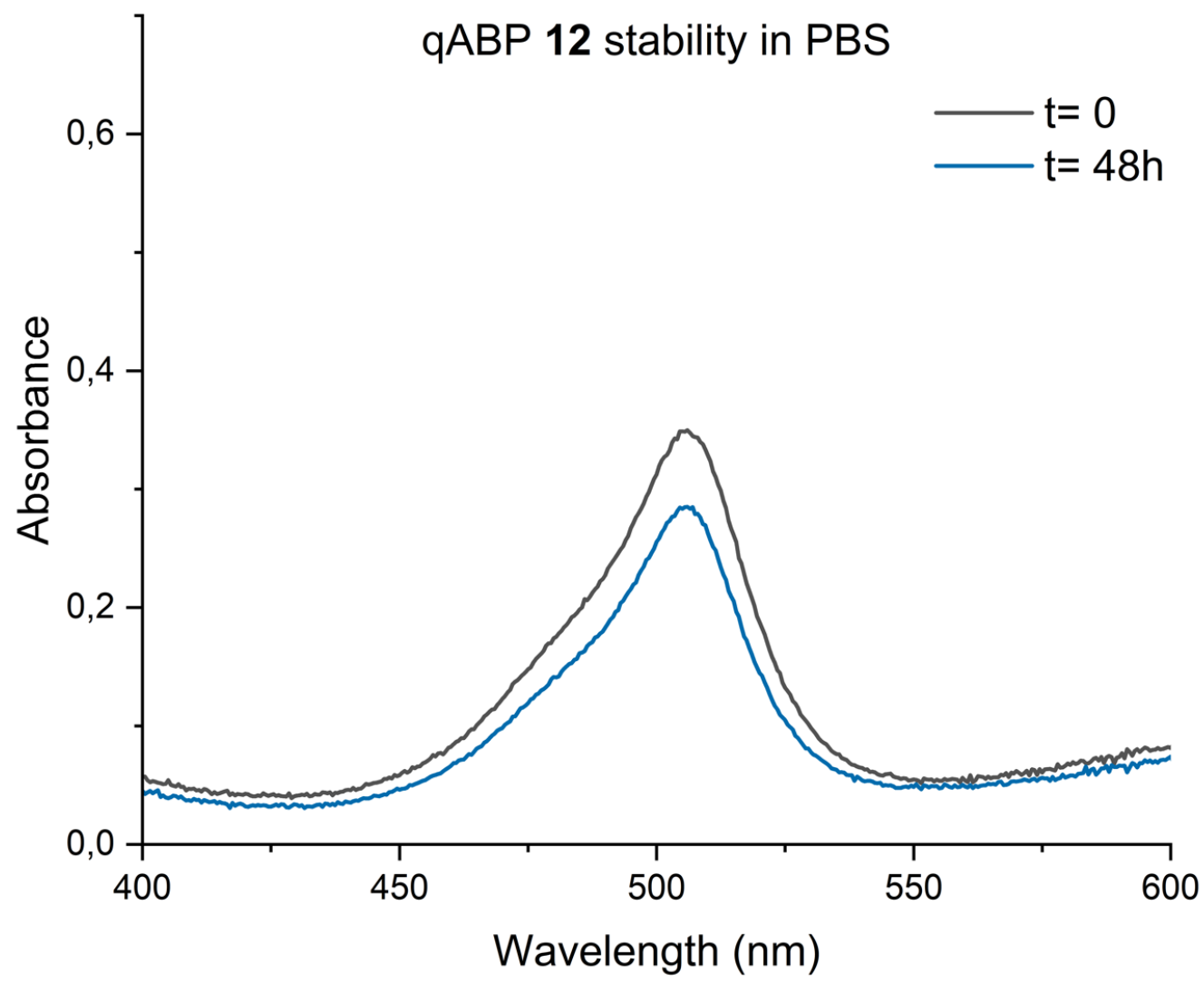

Figure S23. UV-spectra of qABP **12** (1µM in PBS with 0.07% of DMSO) measure at different time points, until 48 hours.

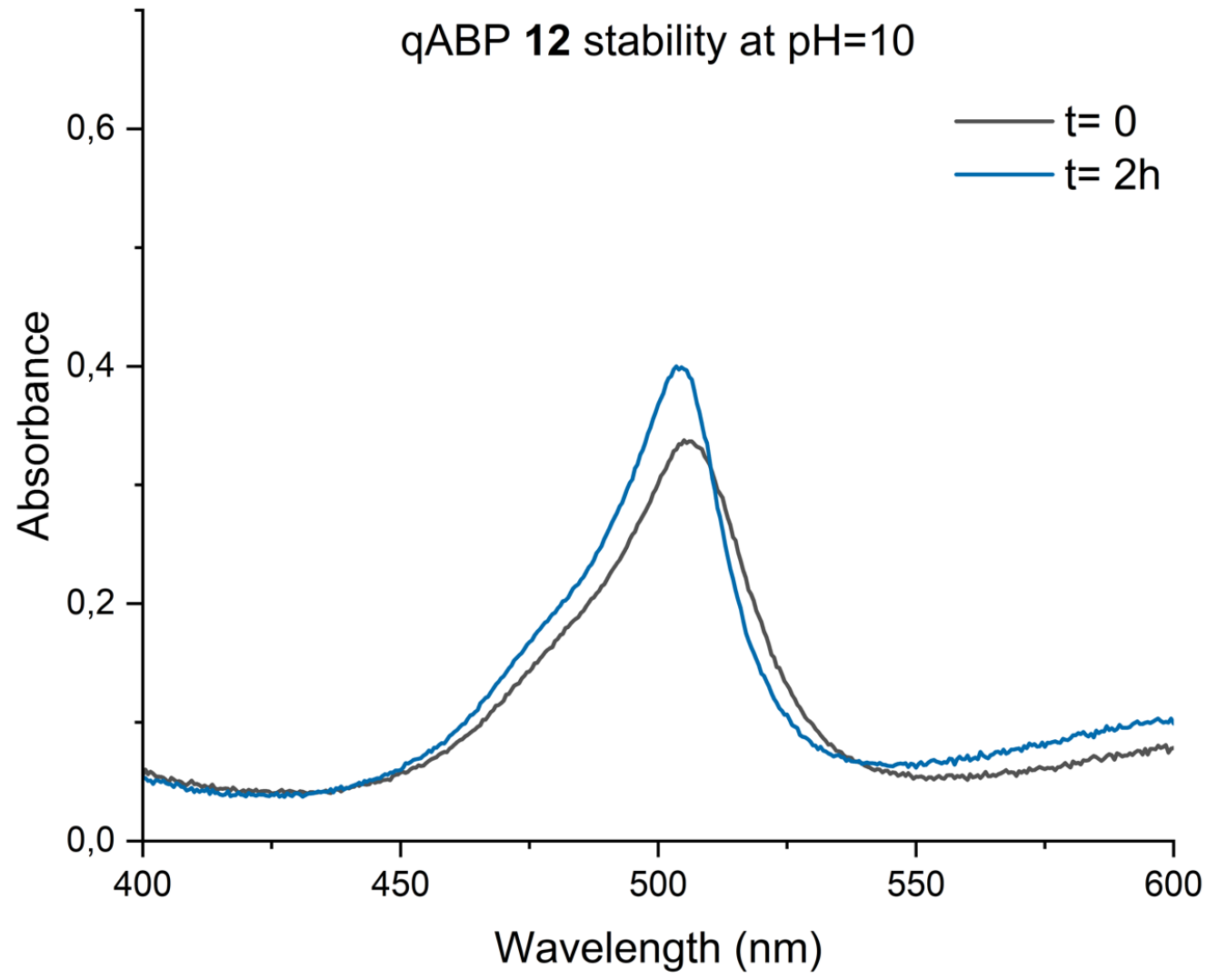

Figure S24.UV-spectra of qABP **12** (1µM at pH=10 with 0.07% of DMSO) measure at different time points, until 2 hours.

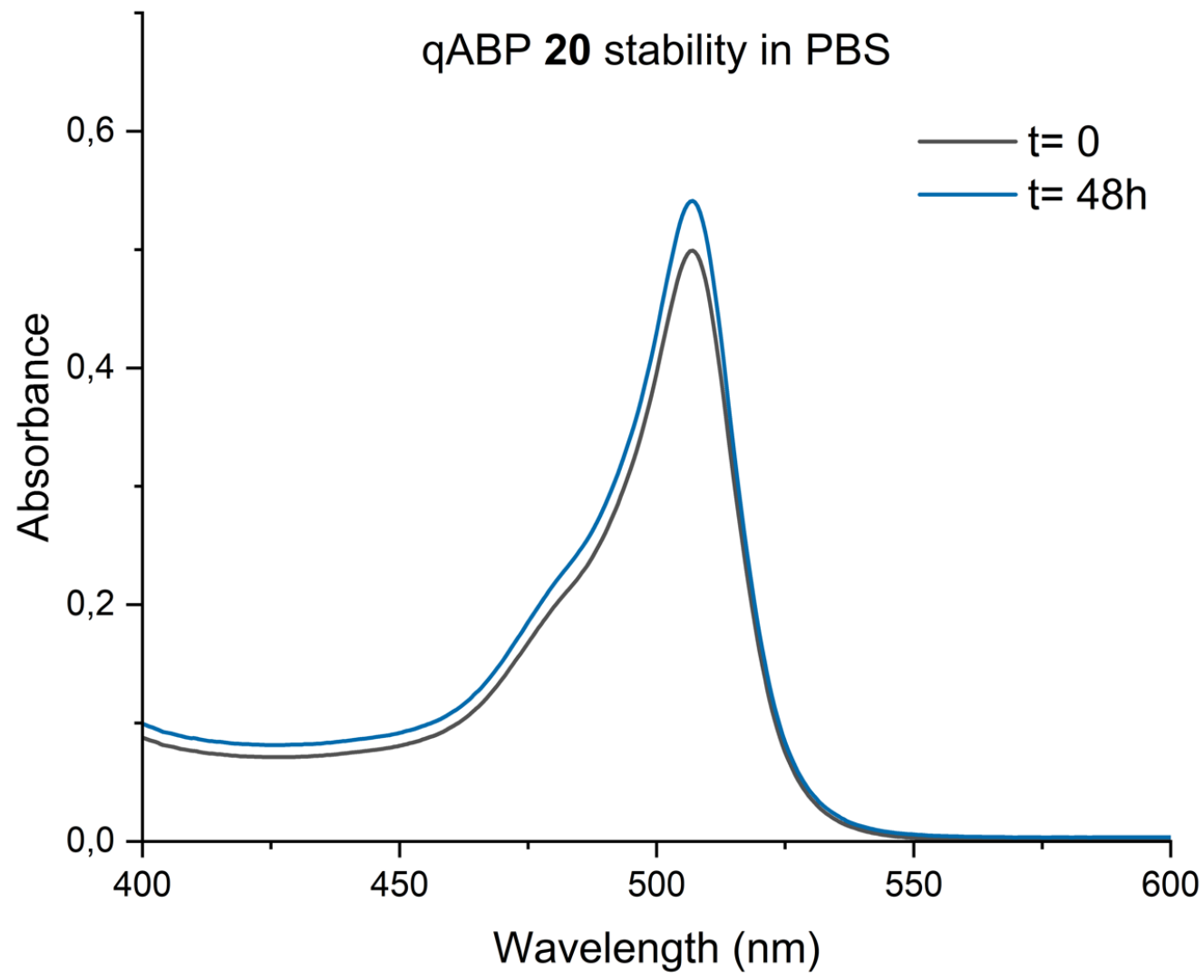

Figure S25.UV-spectra of qABP **20** (1µM in PBS with 20% of DMSO) measured at different time points, until 48 hours.

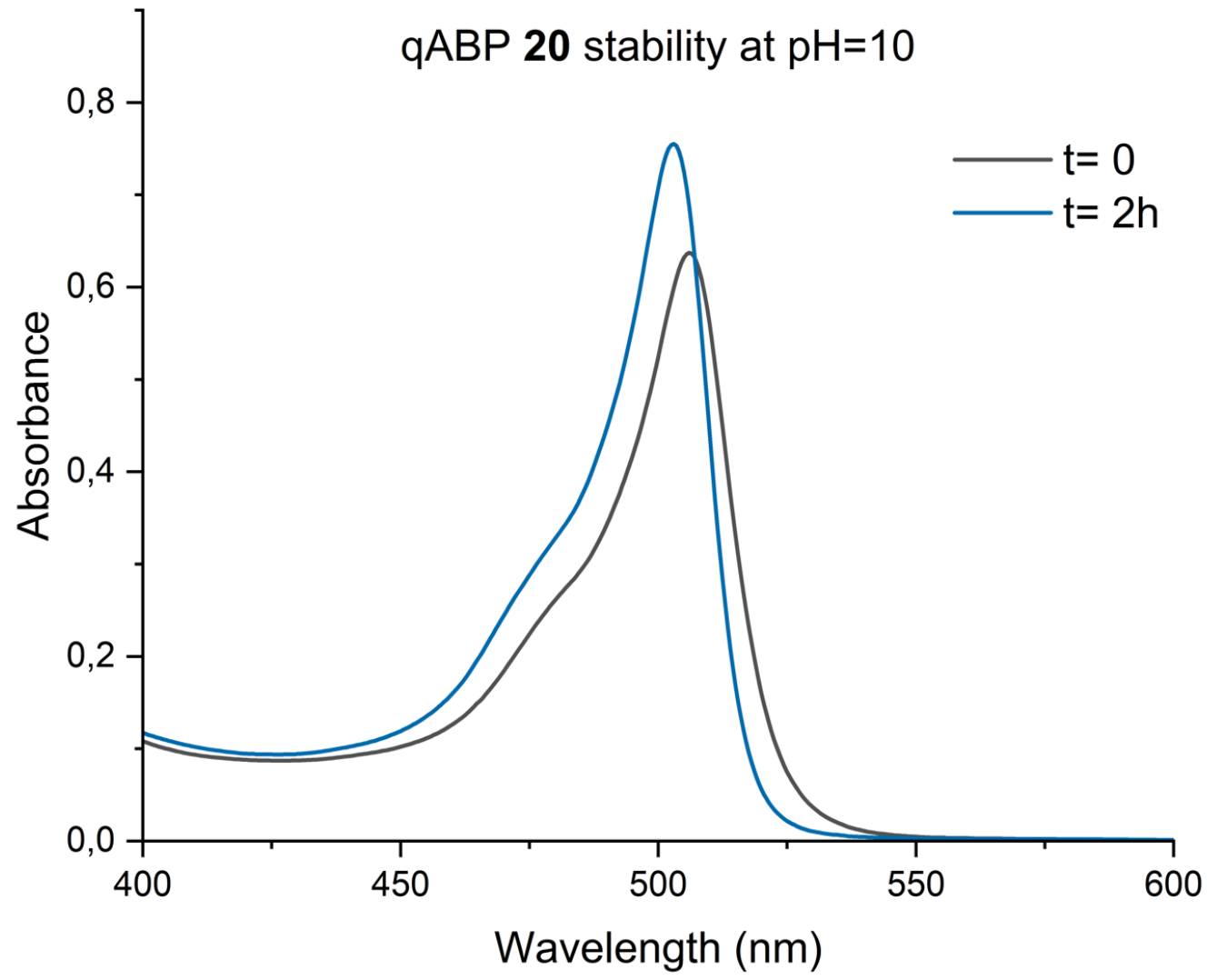

Figure S26.UV-spectra of qABP **20** (1μM at pH=10 with 20% of DMSO) measure at different time points, until 2 hours.

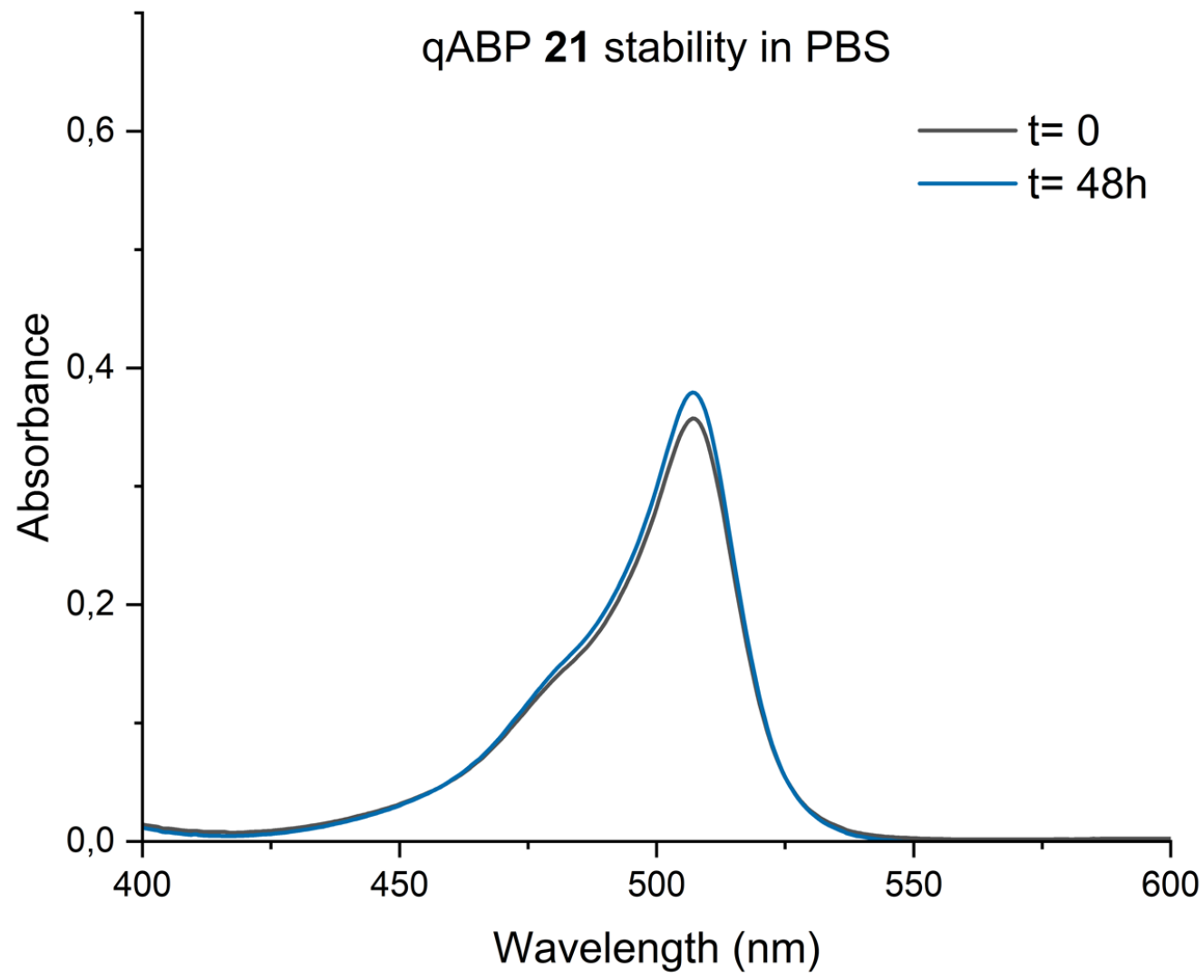

Figure S27.UV-spectra of qABP **21** (1μM in PBS with 20% of DMSO) measured at different time points, until 48 hours.

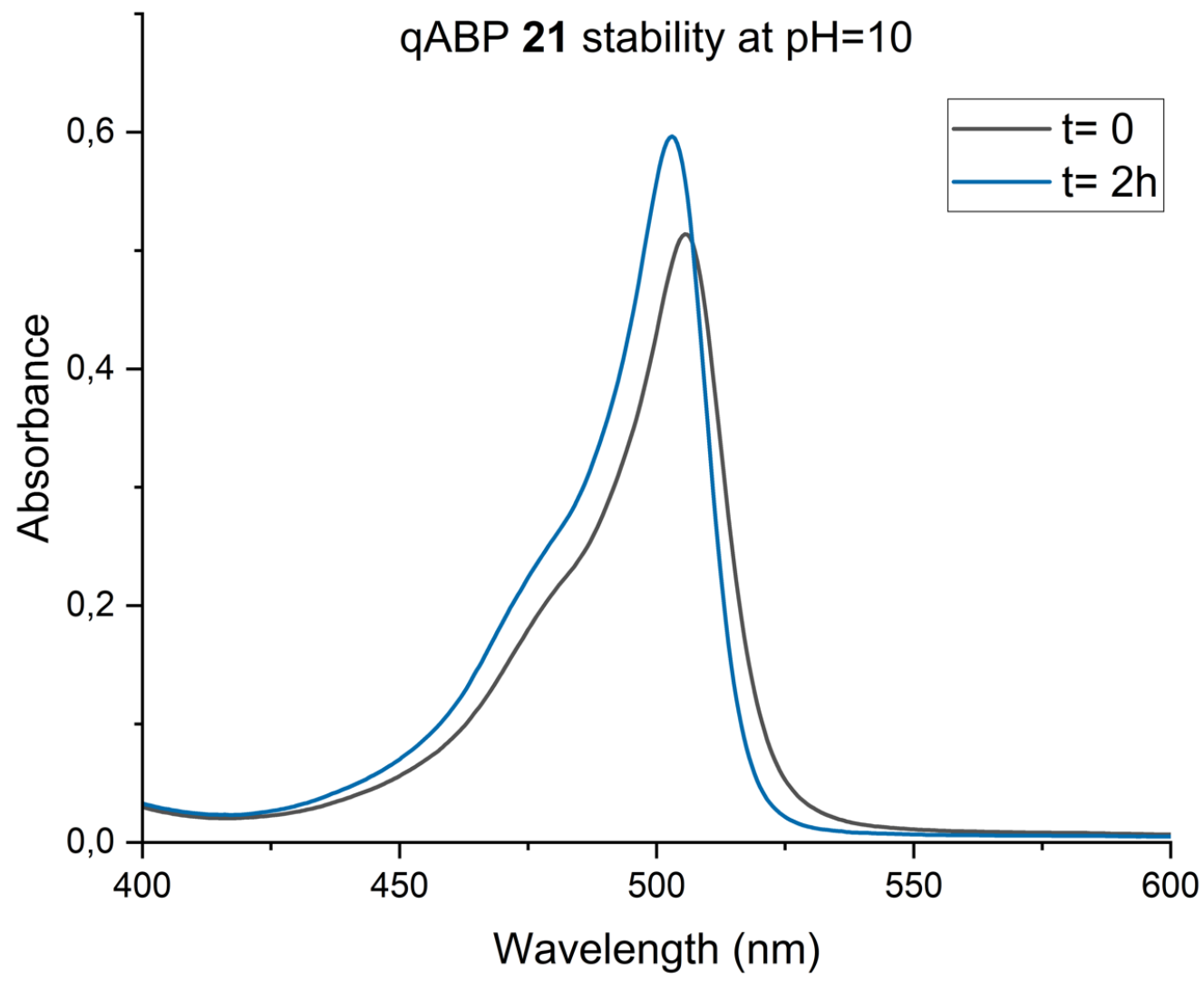

Figure S28.UV-spectra of qABP **21** (1µM at pH=10 with 20% of DMSO) measure at different time points, until 2 hours.

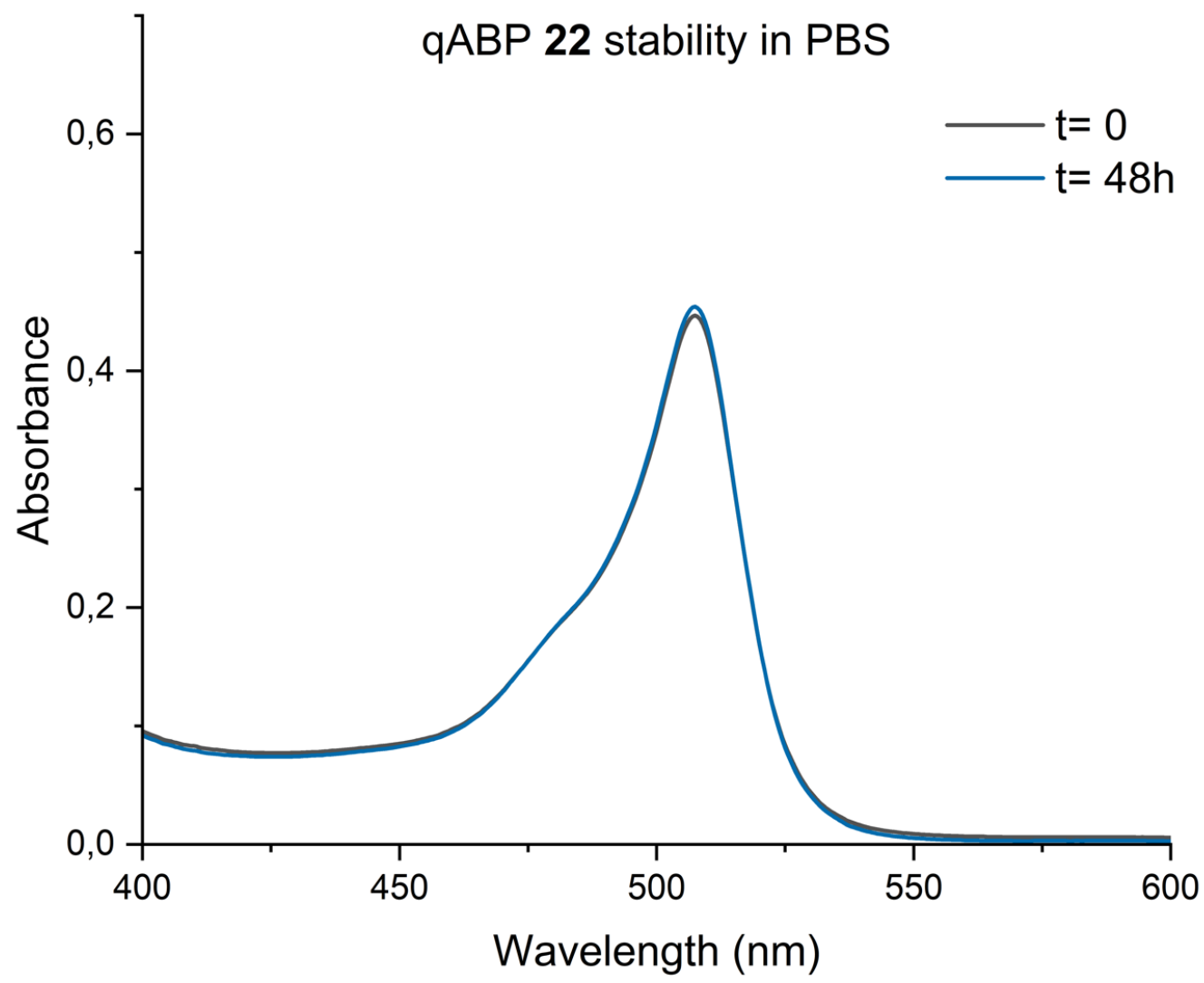

Figure S29.UV-spectra of qABP **22** (1µM in PBS with 20% of DMSO) measured at different time points, until 48 hours.

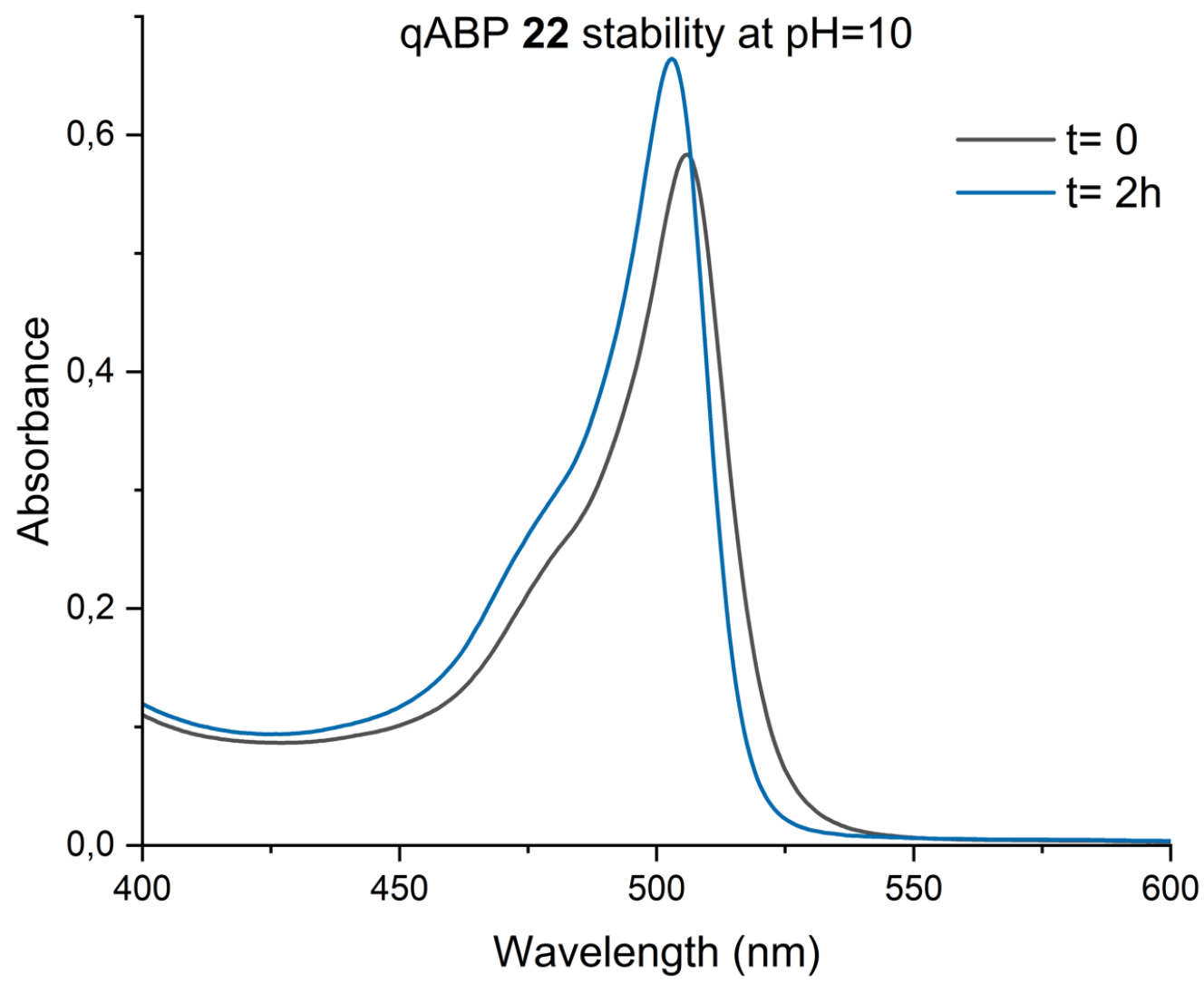

Figure S30. UV-spectra of qABP **22** (1µM at pH=10 with 20% of DMSO) measure at different time points, until 2 hours.

Fluorescence of the qABPs before and after activation

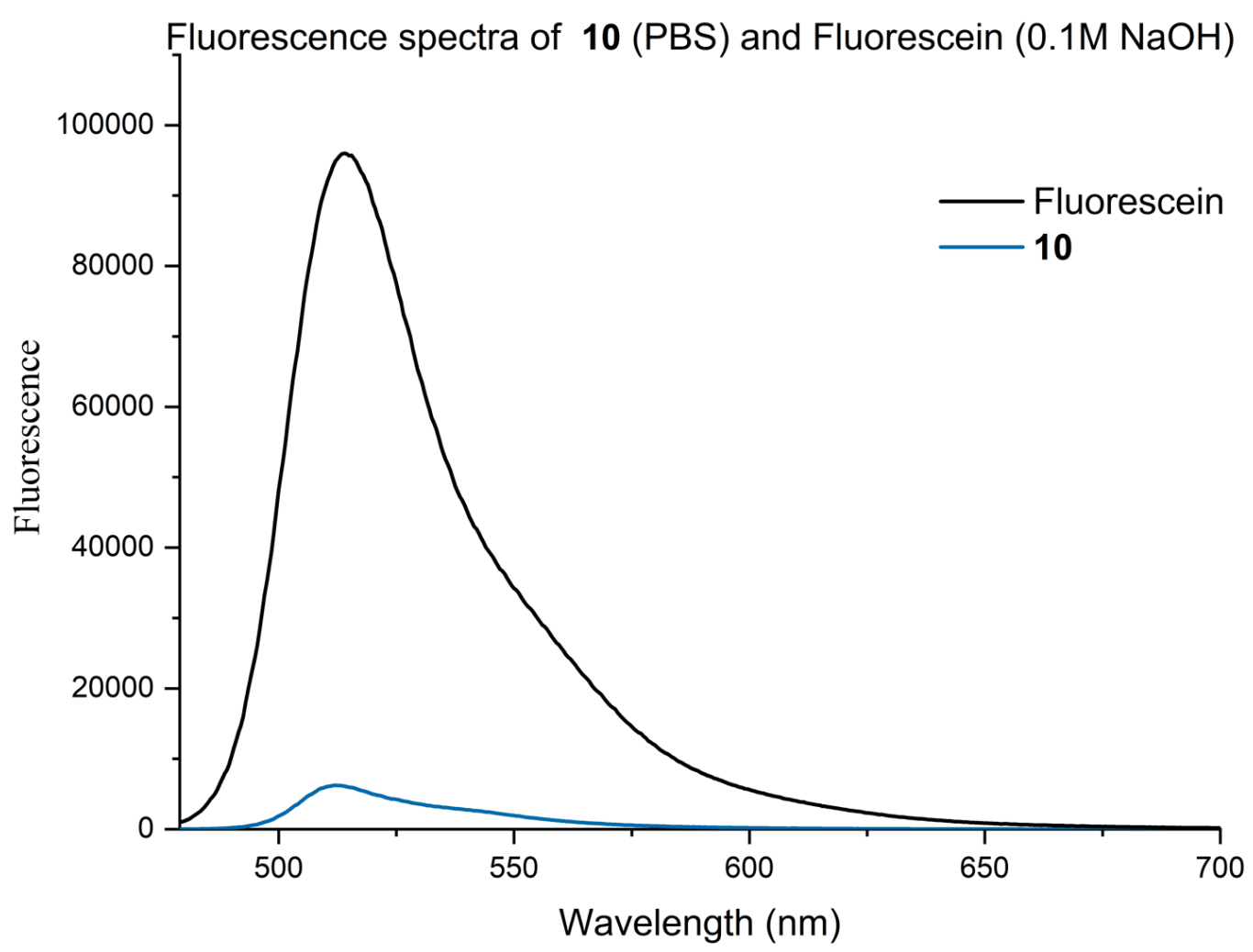

Figure S31. Fluorescence spectra of fluorescein in a solution of 0.1M NaOH, and **10** (1µM) in PBS. Excitation wavelength: 484 nm. Emission wavelength: 487 nm.

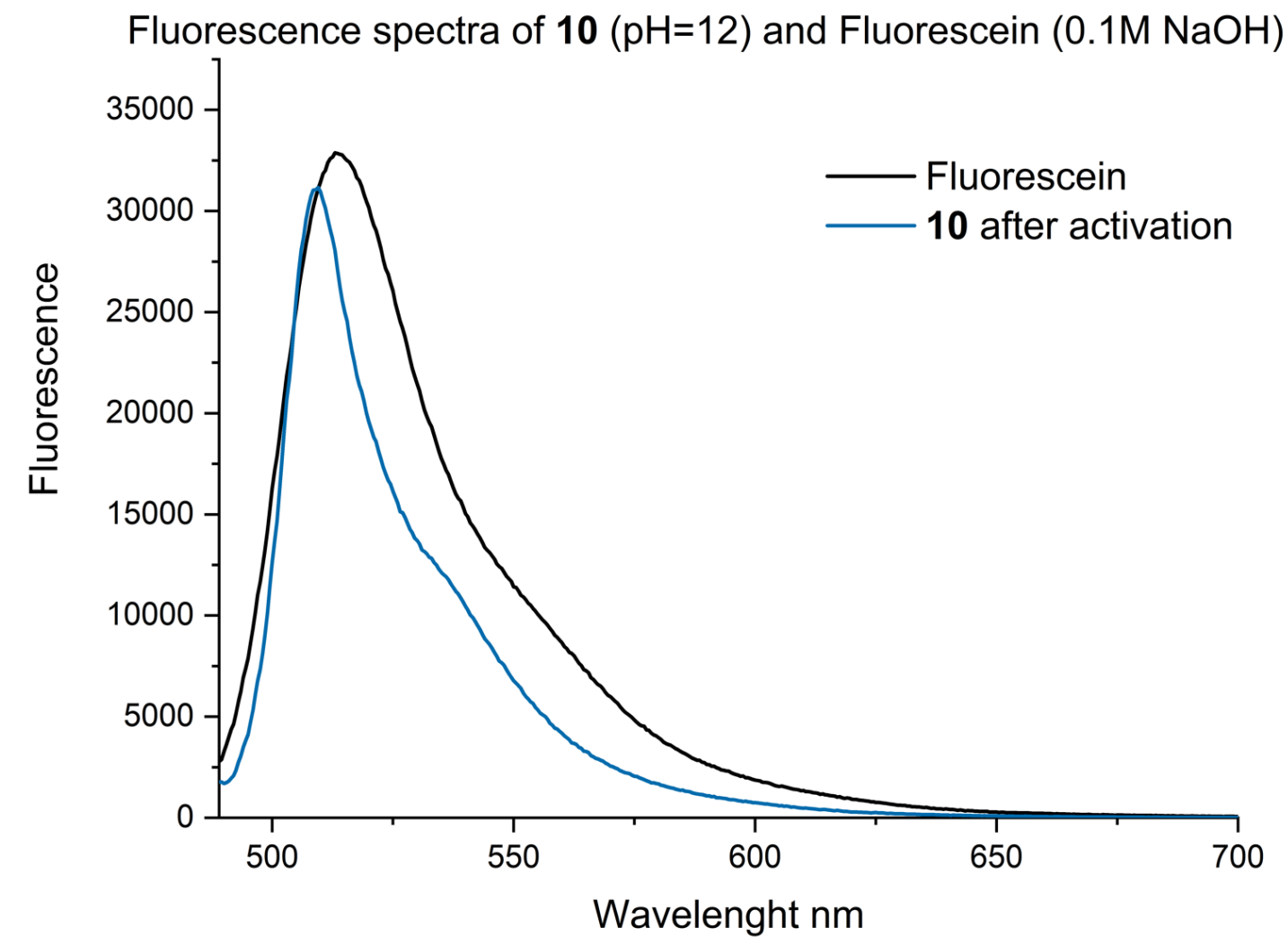

Figure S32. Fluorescence spectra of fluorescein in a solution of 0.1M NaOH, and **10** (1μM) at pH=12 after 12 hours of activation. Excitation wavelength: 484 nm. Emission wavelength: 487 nm.

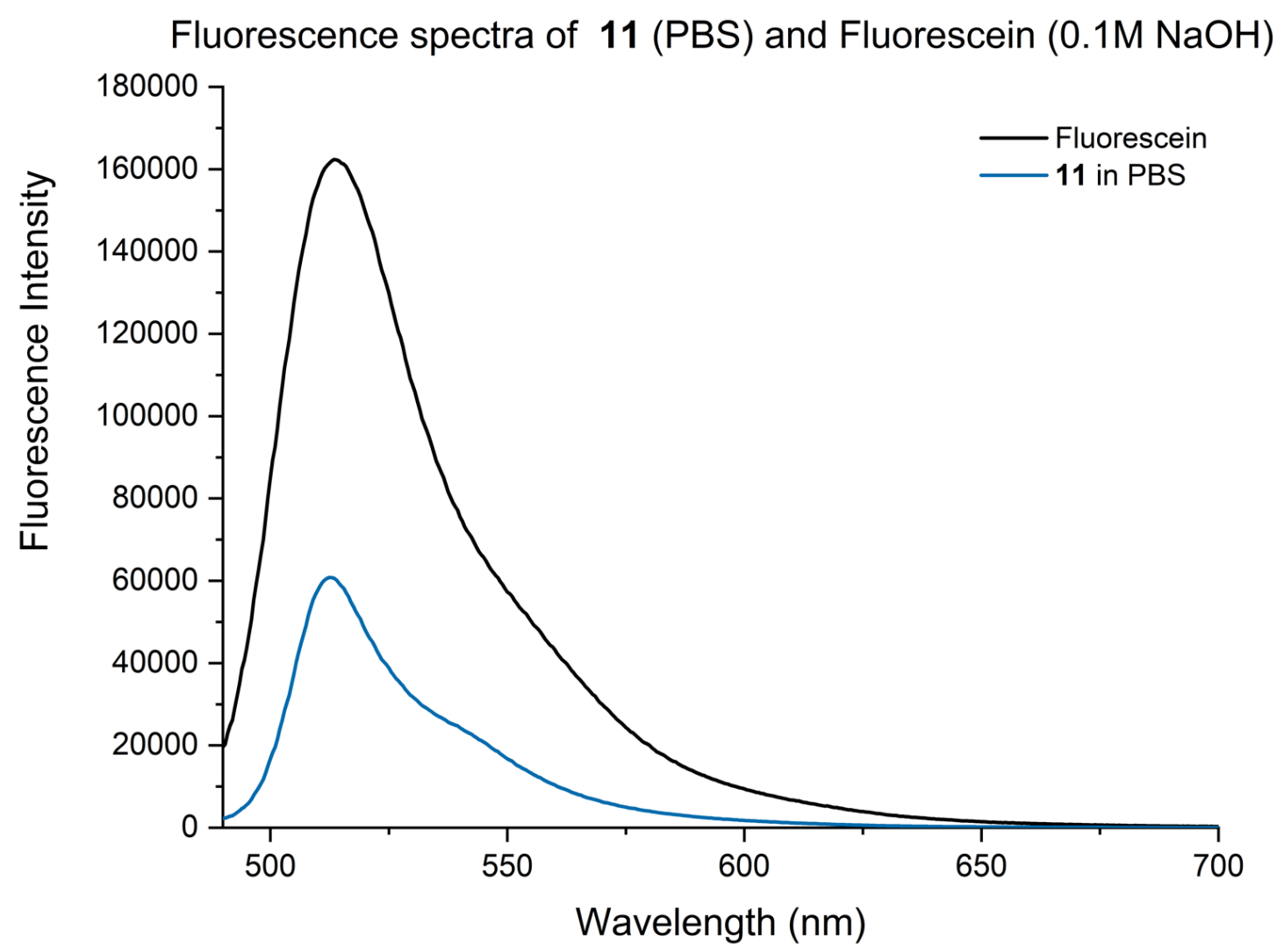

Figure S33. Fluorescence spectra of fluorescein in a solution of 0.1M NaOH, and **11** (1μM) in PBS. Excitation wavelength: 484 nm. Emission wavelength: 487 nm.

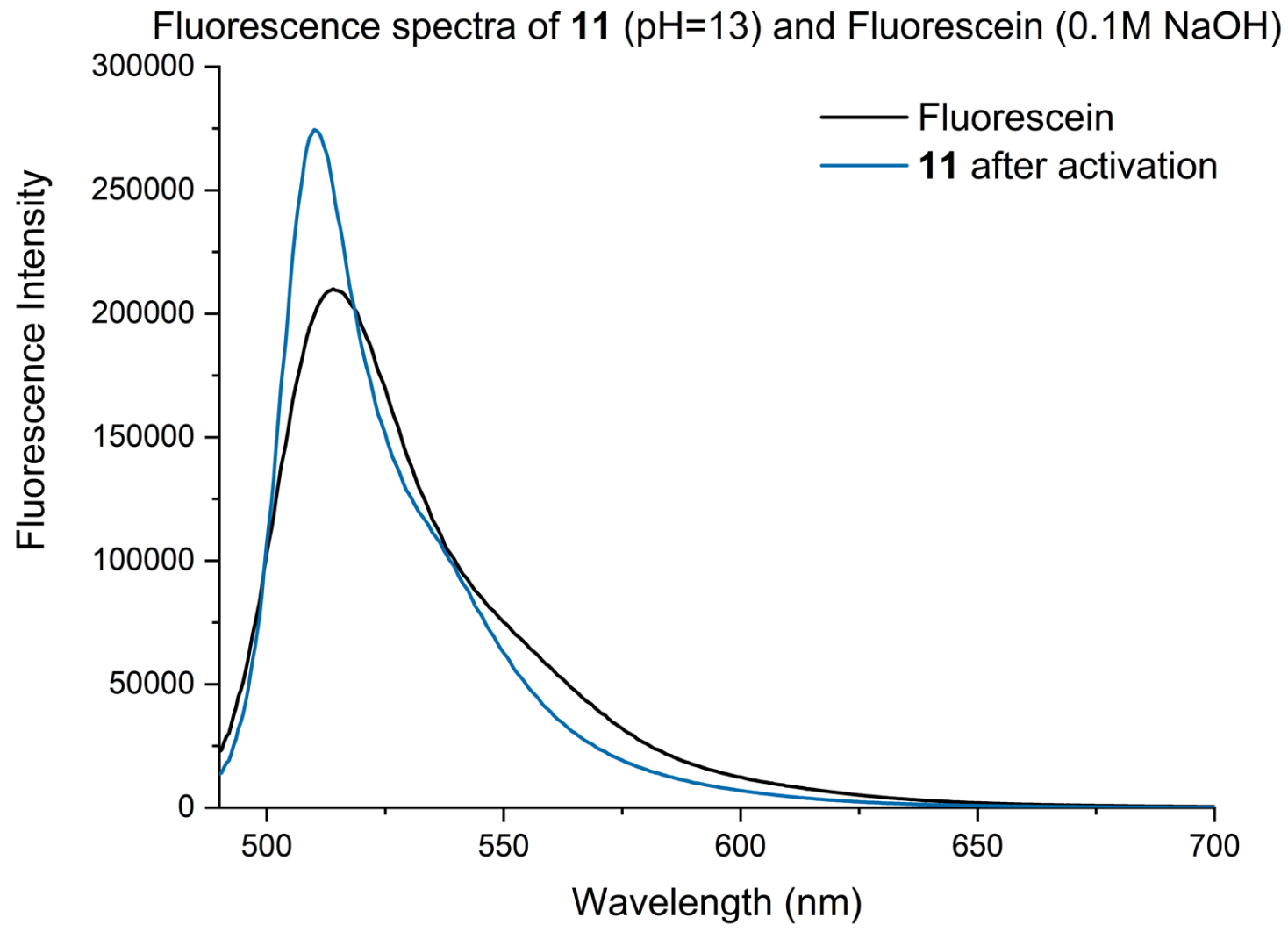

Figure S34.Fluorescence spectra of fluorescein in a solution of 0.1M NaOH, and **11** (1µM) at pH=12 after 12 hours of activation. Excitation wavelength: 484 nm. Emission wavelength: 487 nm.

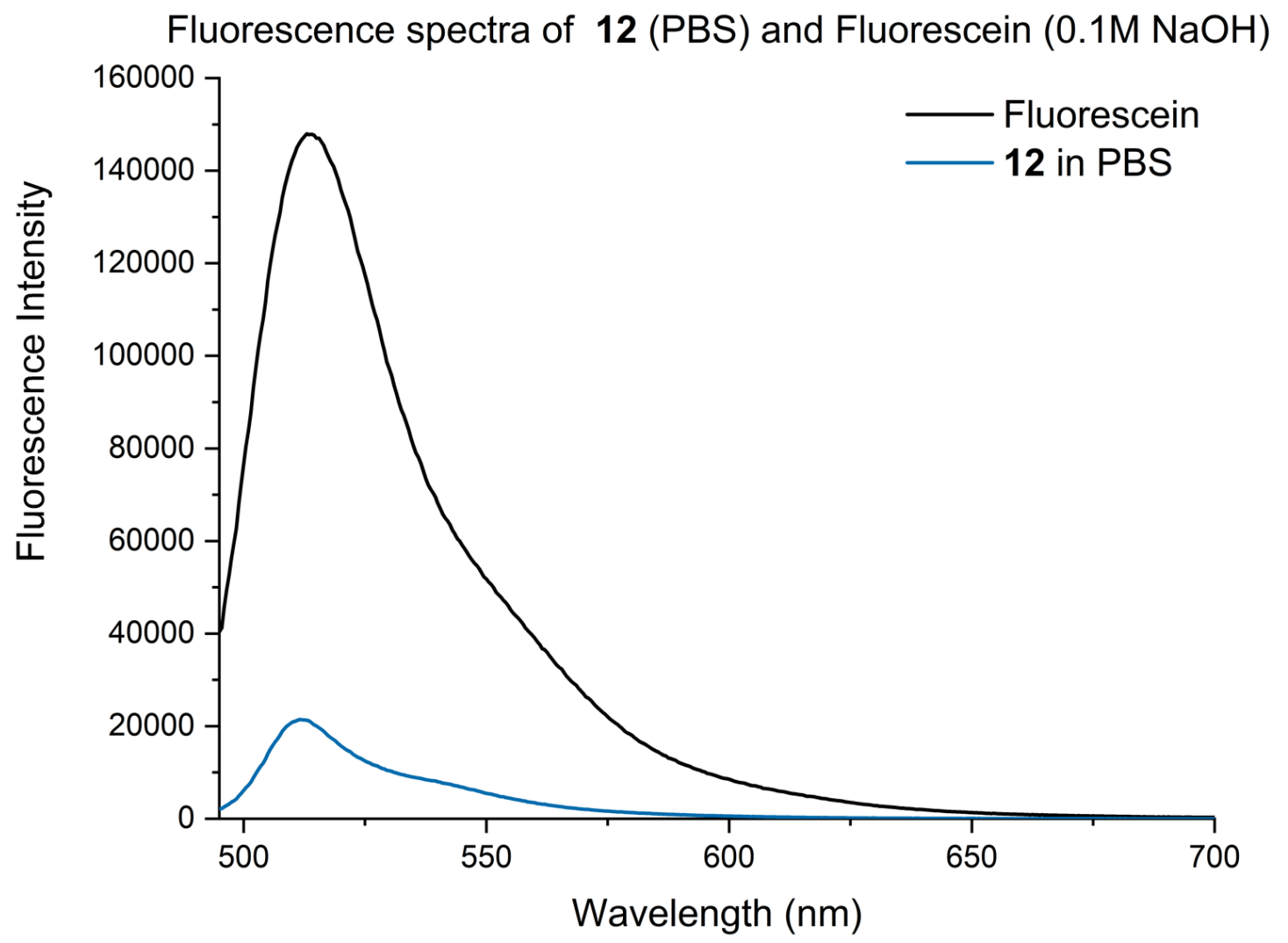

Figure S35. Fluorescence spectra of fluorescein in a solution of 0.1M NaOH, and **12** (1µM) in PBS. Excitation wavelength: 484 nm. Emission wavelength: 487 nm.

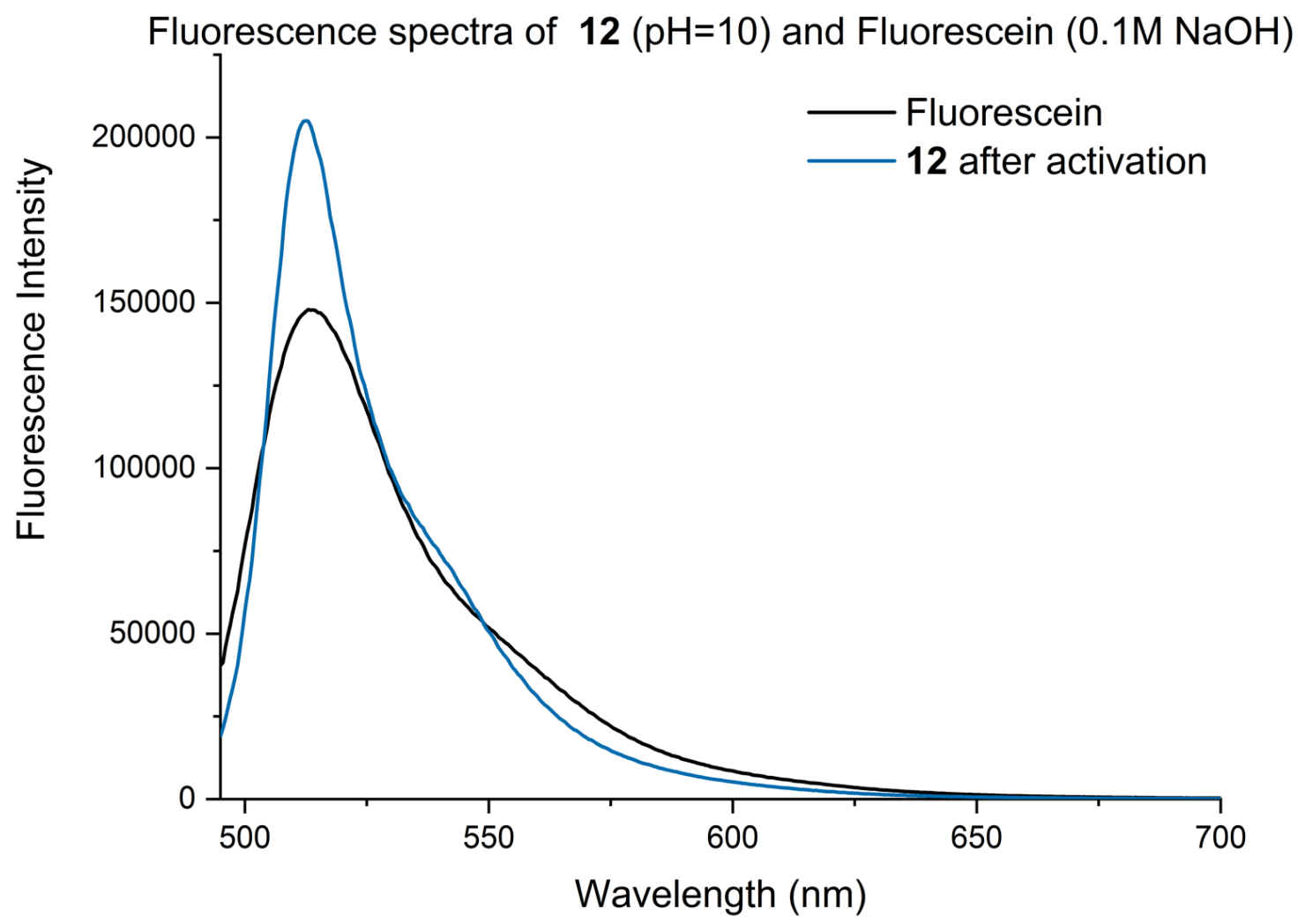

Figure S36. Fluorescence spectra of fluorescein in a solution of 0.1M NaOH, and **12** (1μM) at pH=10 after 2 hours of activation. Excitation wavelength: 484 nm. Emission wavelength: 487 nm.

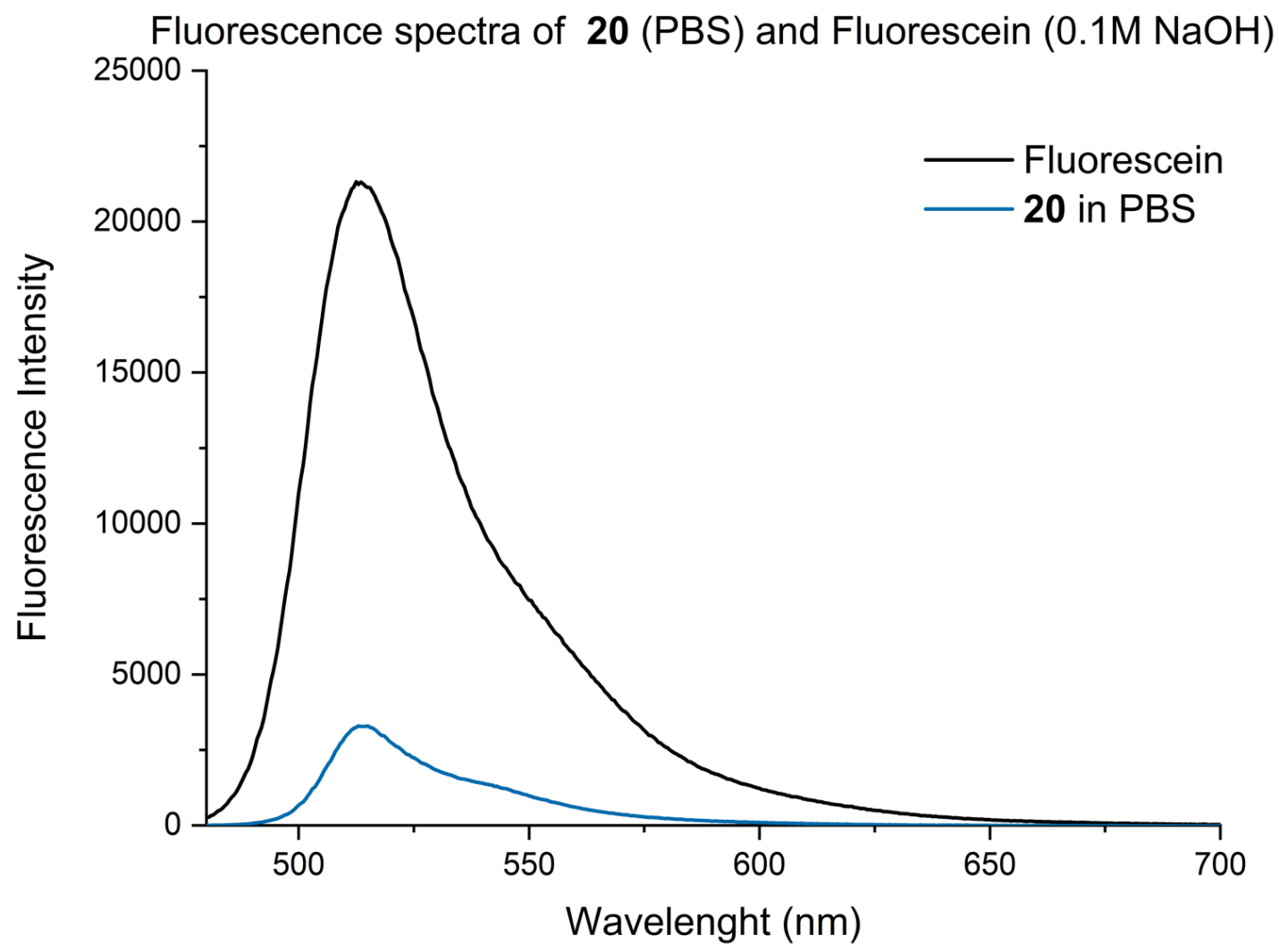

Figure S37. Fluorescence spectra of fluorescein in a solution of 0.1M NaOH, and **20** (1μM) in PBS. Excitation wavelength: 484 nm. Emission wavelength: 487 nm.

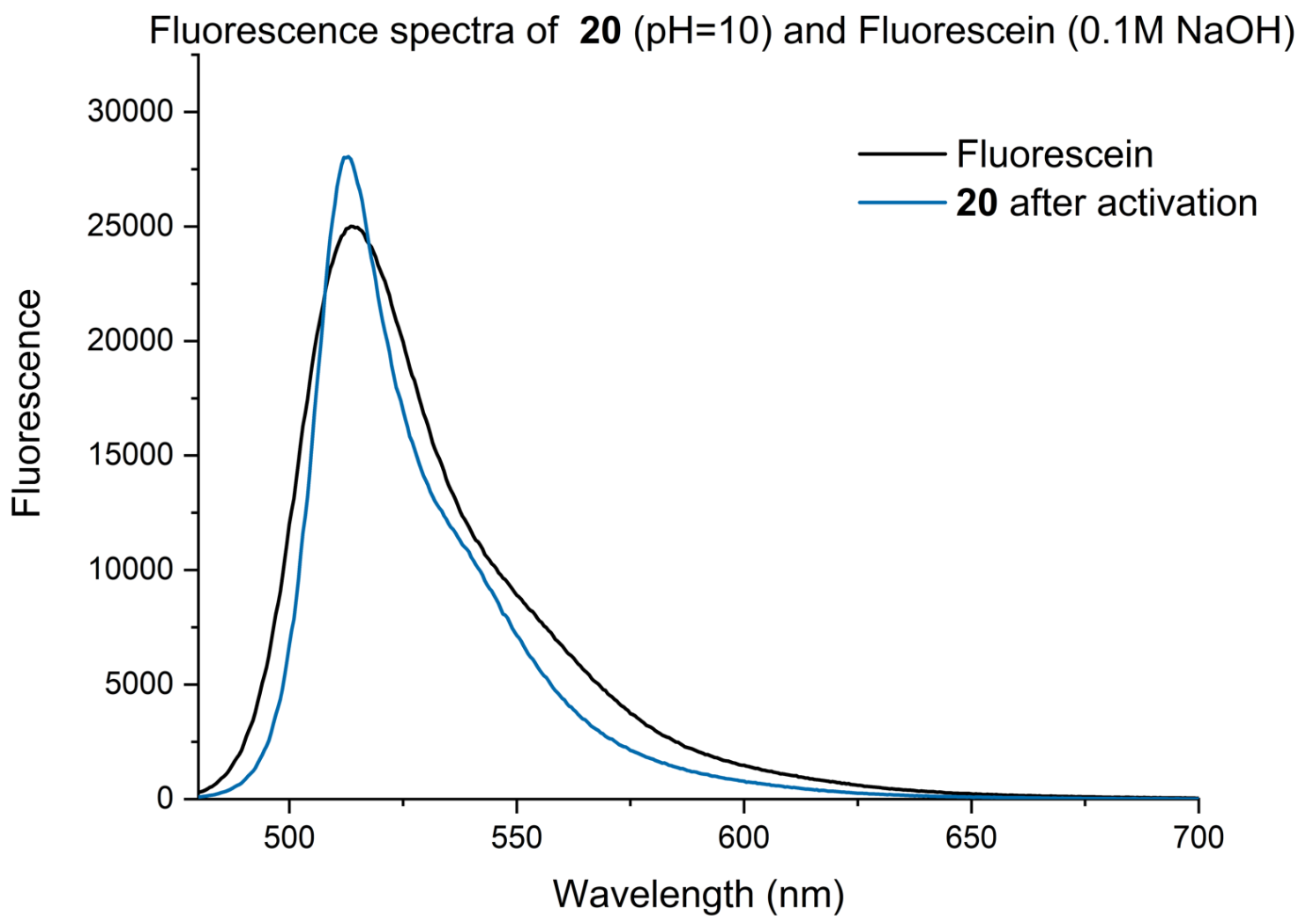

Figure S38. Fluorescence spectra of fluorescein in a solution of 0.1M NaOH, and **20** (1μM) at pH=10 after 2 hours of activation. Excitation wavelength: 484 nm. Emission wavelength: 487 nm.

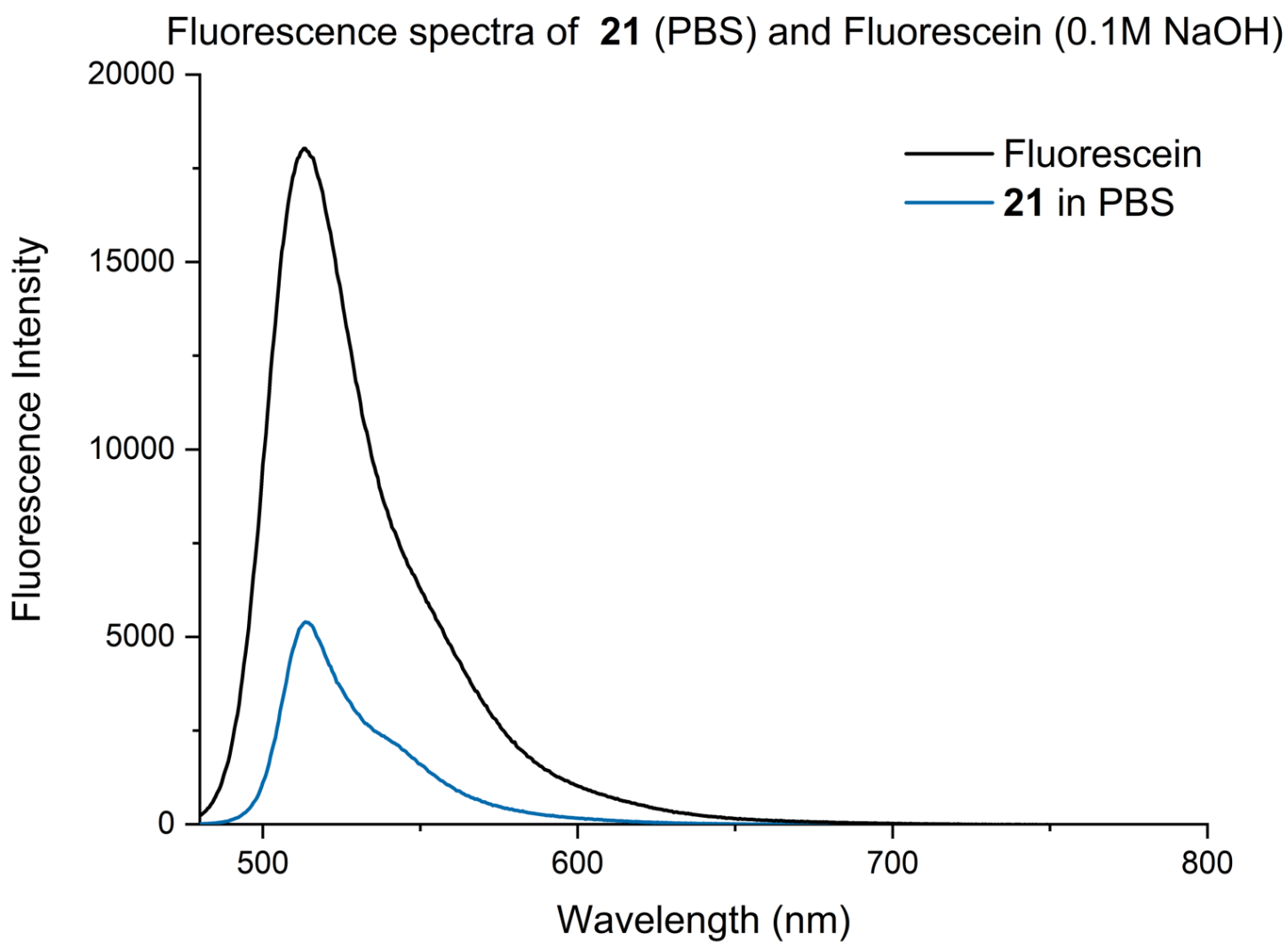

Figure S39. Fluorescence spectra of fluorescein in a solution of 0.1M NaOH, and **21** (1μM) in PBS. Excitation wavelength: 484 nm. Emission wavelength: 487 nm.

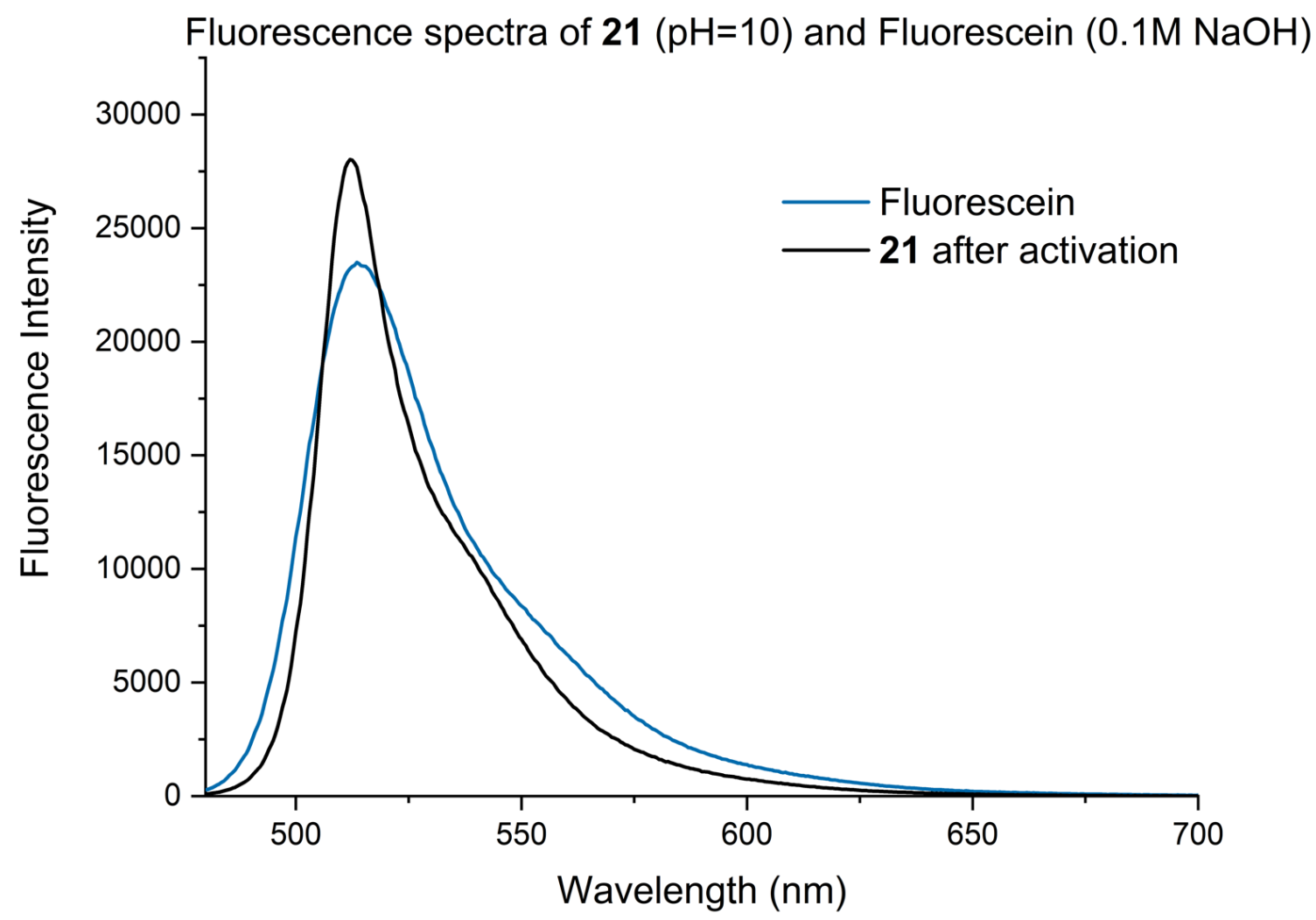

Figure S40. Fluorescence spectra of fluorescein in a solution of 0.1M NaOH, and **21** (1μM) at pH=10 after 2 hours of activation. Excitation wavelength: 484 nm. Emission wavelength: 487 nm.

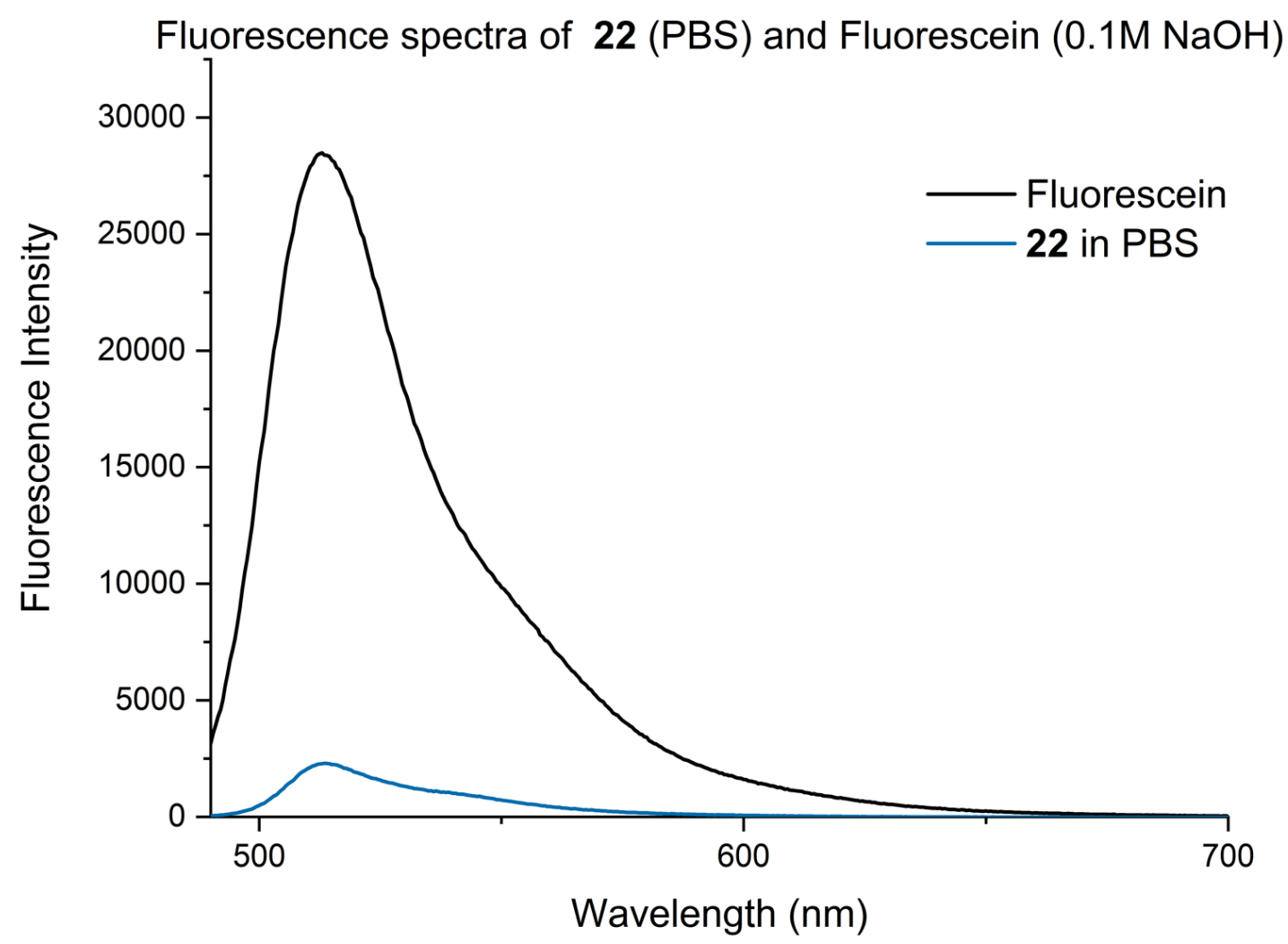

Figure S41. Fluorescence spectra of fluorescein in a solution of 0.1M NaOH, and **22** (1μM) in PBS. Excitation wavelength: 484 nm. Emission wavelength: 487 nm.

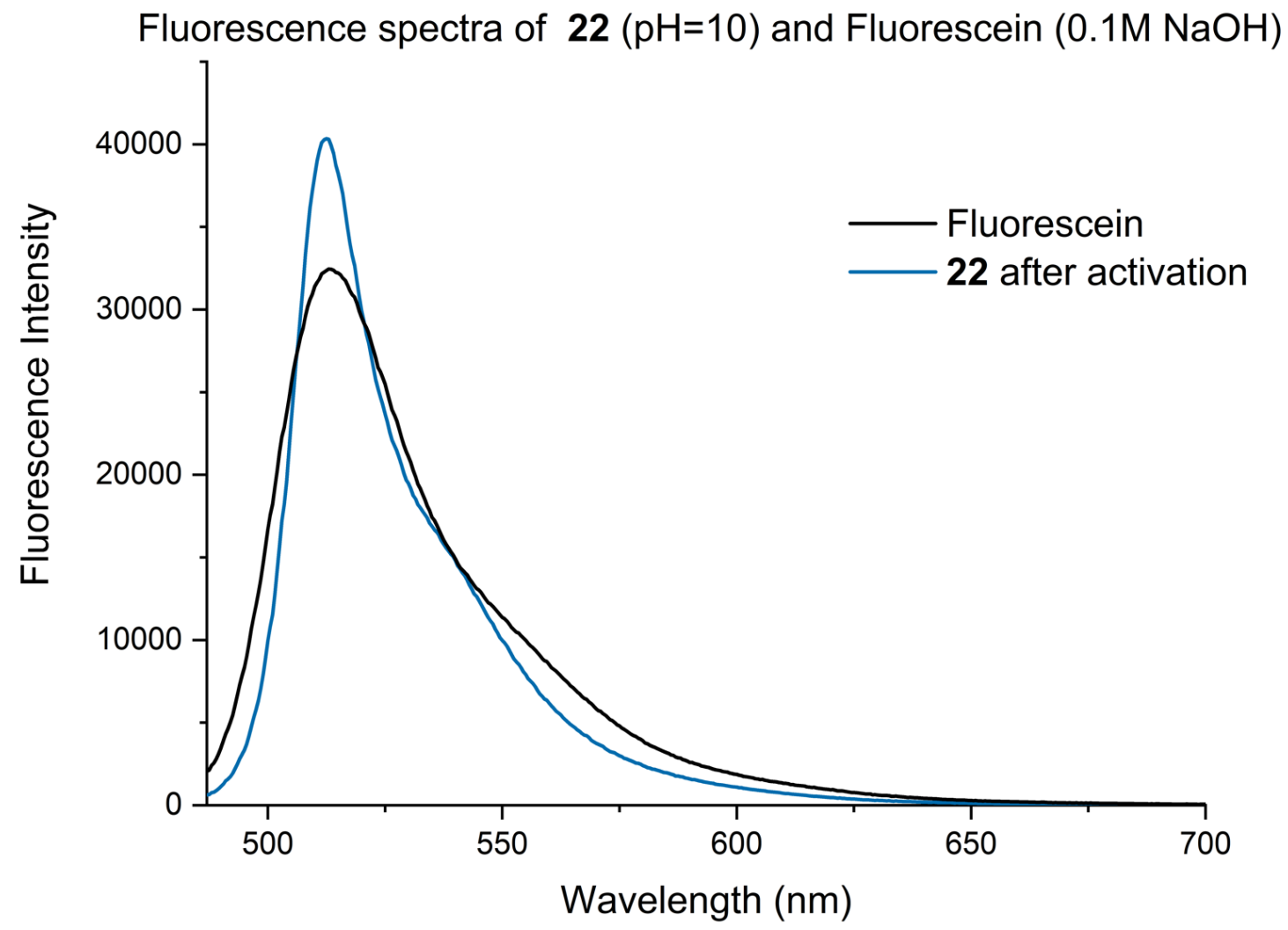

Figure S42. Fluorescence spectra of fluorescein in a solution of 0.1M NaOH, and **22** (1μM) at pH=10 after 2 hours of activation. Excitation wavelength: 484 nm. Emission wavelength: 487 nm.

Table S1. Summary of the calculated brightness values for the qABPs, derived from their photophysical parameters.

qABP	Φ_F	Brightness ($M^{-1}cm^{-1}$)
**20** (PBS)	11	$5{,}94 \times 10^4$
**20** (after activation)	79	$6{,}00 \times 10^5$
**21** (PBS)	21	$7{,}98 \times 10^4$
**21** (after activation)	77	$4{,}62 \times 10^5$
**22** (PBS)	6	$2{,}70 \times 10^4$
**22** (after activation)	80	$5{,}28 \times 10^5$

Activation of qABPs with Pancreatic Porcine Elastase

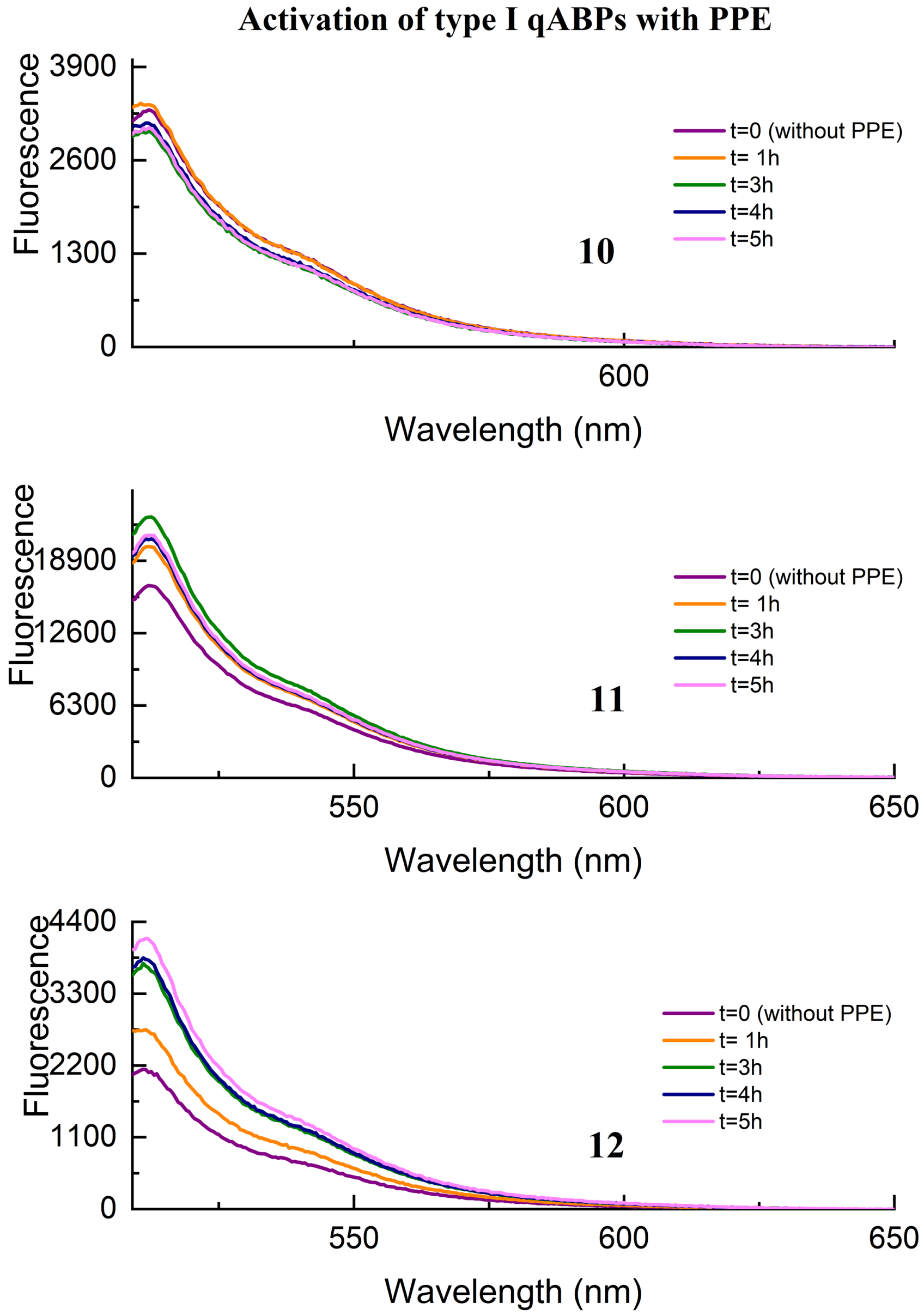

Figure S43. Fluorescence spectra over time of the activation of **10**, **11** and **12** (1.5 µM in 100 mM HEPES buffer with 0,07% DMSO, pH 7.4 at 37 °C.) with PPE. Excitation wavelength: 484 nm. Emission wavelength: 487 nm.

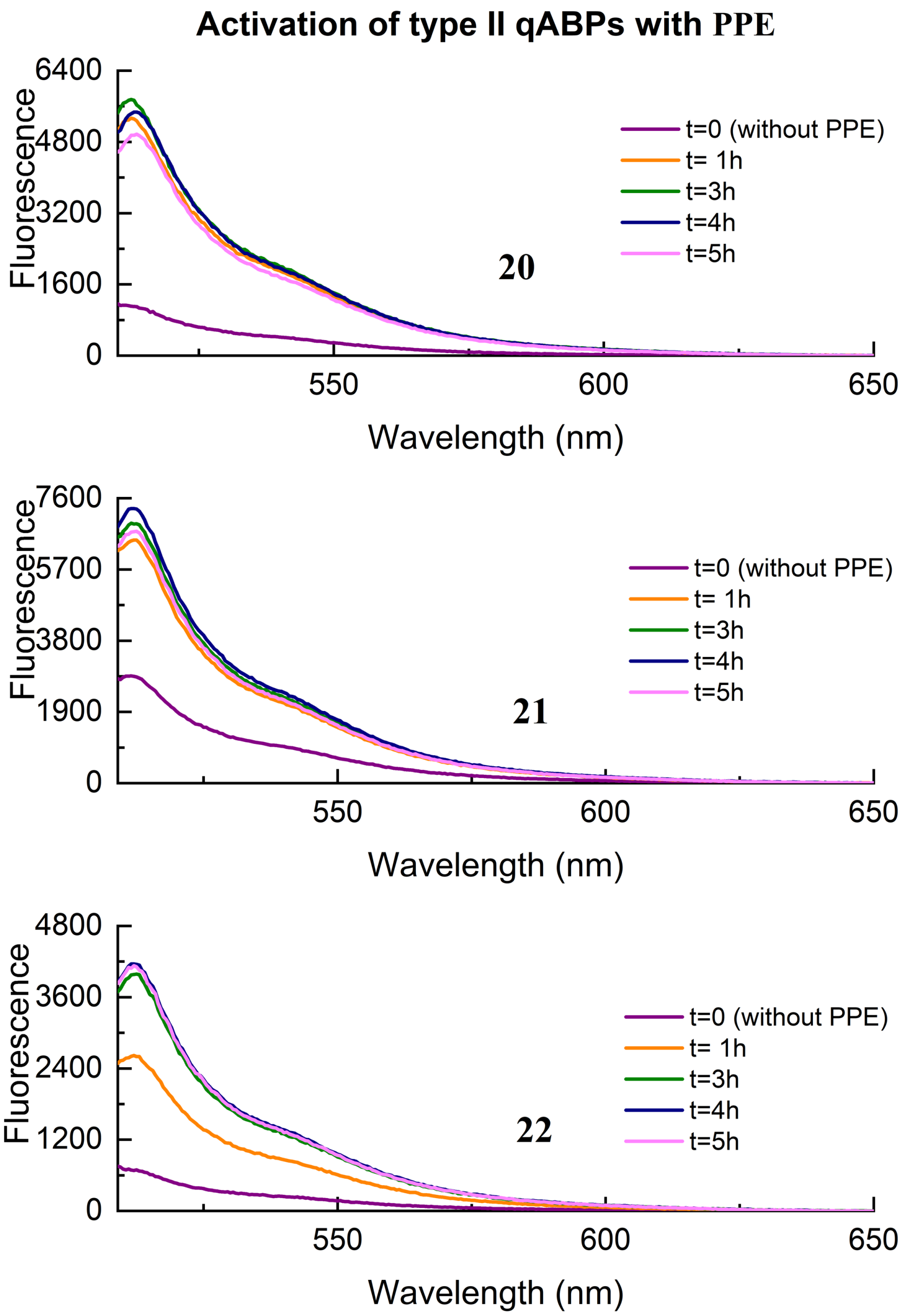

Figure S44. Fluorescence spectra over time of the activation of **20**, **21** and **22** (1.5 µM in 100 mM HEPES buffer with 20% DMSO, pH 7.4 at 37 °C.) with PPE. Excitation wavelength: 484 nm. Emission wavelength: 487 nm.

Jump Dillution Assay

HNE (2 µM) was incubated with the qABPs **19iii** and **22** (20 µM) in PBS pH7.4 for 1 h at 37 °C. After this period, the mixtures were diluted 100-fold into PBS (pH 7.4). The substrate MeOSuc-Ala-Ala-Pro-Val-pNA was then added to reach a final concentration of 1 mM. Absorbance was monitored every 30 s for 1 h, and the data are shown as changes in absorbance relative to the moment of substrate addition. (Figure S45)

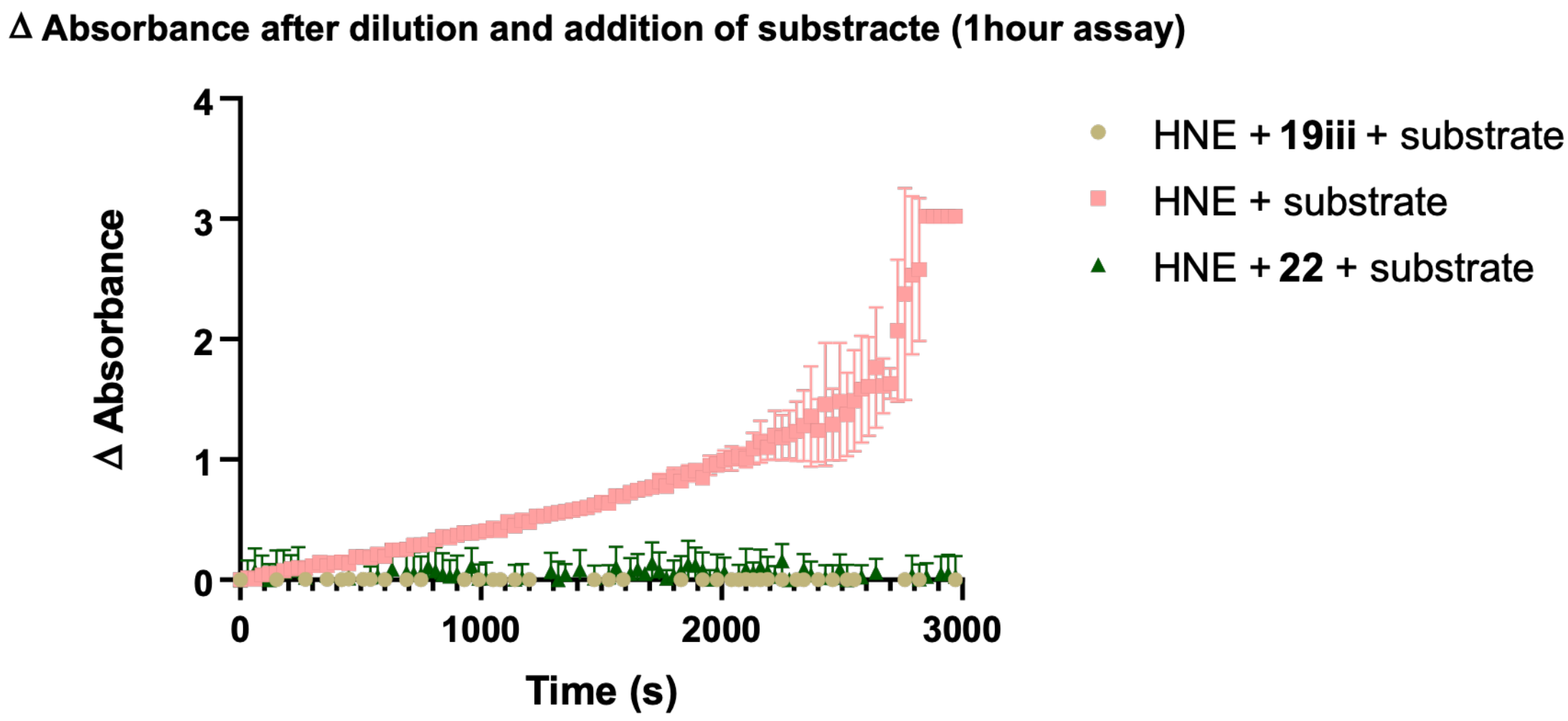

Figure S45. Time dependent absorbance traces. For the samples pre incubated with the qABPs, the absorbance remains unchanged after substrate addition, indicating that the enzyme has been irreversibly inactivated.

qABPs inhibitory ability against HNE

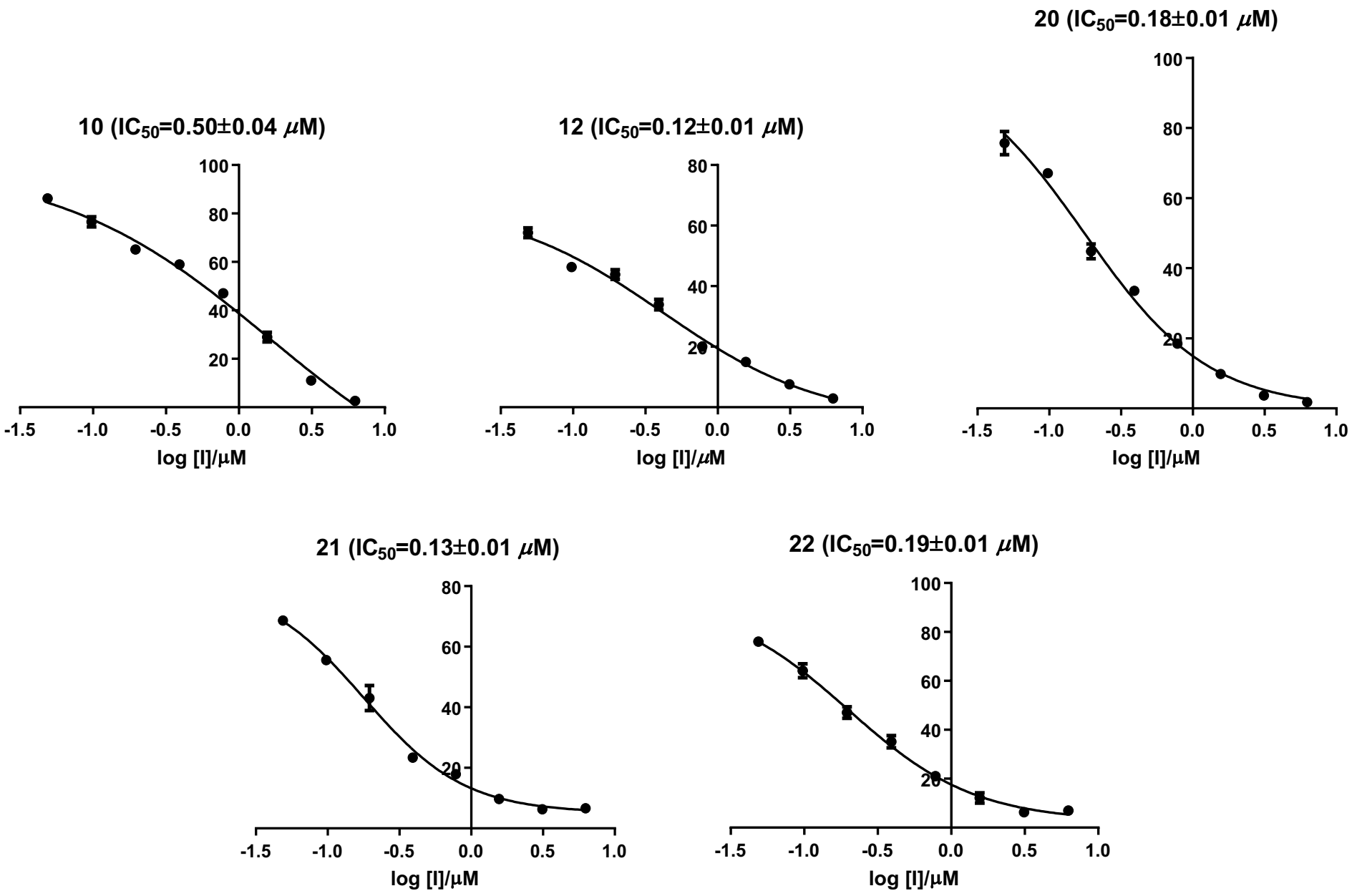

Figure S46. Dose-response curves for HNE inhibition by the synthesised qABPs

Table S2. IC$_{50}$ values of HNE inhibition of the synthesised qABPs. (All experiments were run in triplicate).

	qABP	IC$_{50}$ (μM)	SD (μM)
Type I	**10**	0.50	0.04
	**12**	0.12	0.01
Type II	**20**	0.18	0.01
	**21**	0.13	0.01
	**22**	0.19	0.01

Docking results

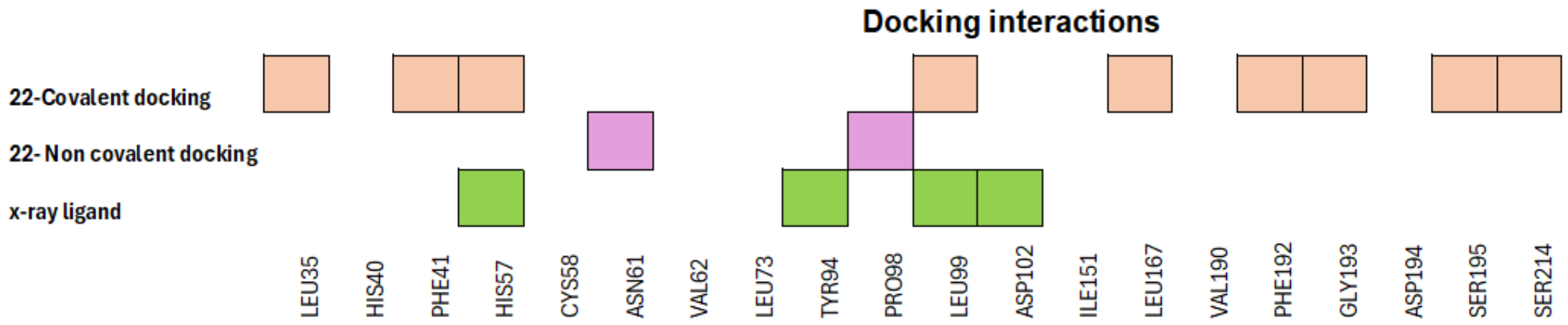

Figure S47. Covalent and no-covalent docking interactions of qABP **22** with HNE.

Table S3. Residue equivalence table between the crystallographic structures of HNE, 1HNE and 9SI4.

1HNE	9SI4	1HNE	9SI4	1HNE	9SI4	1HNE	9SI4	1HNE	9SI4
16 ILE	16 ILE	63 ARG	64 ARG	111 SER	112 SER	160 VAL	160 VAL	219 GLY	208 GLY
17 VAL	17 VAL	64 ALA	65 ALA	112 ALA	113 ALA	162 THR	161 THR	220 CYS	209 CYS
18 GLY	18 GLY	65 VAL	66 VAL	113 THR	114 THR	163 VAL	162 VAL	221 ALA	210 ALA
19 GLY	19 GLY	65A ARG	67 ARG	114 ILE	115 ILE	164 VAL	163 VAL	222 SER	211 SER
20 ARG	20 ARG	66 VAL	68 VAL	115 ASN	116 ASN	165 THR	164 THR	222A GLY	212 GLY
21 ARG	21 ARG	67 VAL	69 VAL	116 ALA	117 ALA	166 SER	165 SER	223 LEU	213 LEU
22 ALA	22 ALA	68 LEU	70 LEU	117 ASN	118 ASN	167 LEU	166 LEU	224 TYR	214 TYR
23 ARG	23 ARG	69 GLY	71 GLY	118 VAL	119 VAL	168 CYS	167 CYS	225 PRO	215 PRO
24 PRO	24 PRO	70 ALA	72 ALA	119 GLN	120 GLN	177 ARG	168 ARG	226 ASP	216 ASP
25 HIS	25 HIS	71 HIS	73 HIS	120 VAL	121 VAL	178 ARG	169 ARG	227 ALA	217 ALA
26 ALA	26 ALA	72 ASN	74 ASN	121 ALA	122 ALA	179 SER	170 SER	228 PHE	218 PHE
27 TRP	27 TRP	73 LEU	75 LEU	122 GLN	123 GLN	180 ASN	171 ASN	229 ALA	219 ALA
28 PRO	28 PRO	74 SER	76 SER	123 LEU	124 LEU	181 VAL	172 VAL	230 PRO	220 PRO
29 PHE	29 PHE	75 ARG	77 ARG	124 PRO	125 PRO	182 CYS	173 CYS	231 VAL	221 VAL
30 MET	30 MET	76 ARG	78 ARG	125 ALA	126 ALA	183 THR	174 THR	232 ALA	222 ALA
31 VAL	31 VAL	77 GLU	79 GLU	126 GLN	127 GLN	184 LEU	175 LEU	233 GLN	223 GLN
32 SER	32 SER	78 PRO	80 PRO	127 GLY	128 GLY	185 VAL	176 VAL	234 PHE	223 GLN
33 LEU	33 LEU	79 THR	81 THR	128 ARG	129 ARG	186 ARG	177 ARG	235 VAL	224 PHE
34 GLN	34 GLN	80 ARG	82 ARG	129 ARG	130 ARG	186A GLY	178 GLY	236 ASN	225 VAL
35 LEU	35 LEU	81 GLN	83 GLN	130 LEU	131 LEU	187 ARG	179 ARG	237 TRP	226 ASN
36 ARG	36 ARG	82 VAL	84 VAL	131 GLY	132 GLY	188 GLN	180 GLN	238 ILE	227 TRP
38 GLY	37 GLY	83 PHE	85 PHE	132 ASN	133 ASN	188A ALA	181 ALA	239 ASP	228 ILE
39 GLY	38 GLY	84 ALA	86 ALA	133 GLY	134 GLY	189 GLY	182 GLY	240 SER	229 ASP
40 HIS	39 HIS	85 VAL	87 VAL	134 VAL	135 VAL	190 VAL	183 VAL	241 ILE	230 SER
41 PHE	40 PHE	86 GLN	88 GLN	135 GLN	136 GLN	191 CYS	184 CYS	242 ILE	231 ILE
42 CYS	41 CYS	87 ARG	89 ARG	136 CYS	137 CYS	192 PHE	185 PHE	243 GLN	232 ILE
43 GLY	42 GLY	88 ILE	90 ILE	137 LEU	138 LEU	193 GLY	186 GLY		233 GLN
44 ALA	43 ALA	89 PHE	91 PHE	138 ALA	139 ALA	194 ASP	187 ASP		
45 THR	44 THR	90 GLU	92 GLU	139 MET	140 MET	195 SER	188 SER		
46 LEU	45 LEU	91 ASP	93 ASN	140 GLY	141 GLY	196 GLY	189 GLY		
47 ILE	46 ILE	92 GLY	94 GLY	141 TRP	142 TRP	197 SER	190 SER		
48 ALA	47 ALA	94 TYR	95 TYR	142 GLY	143 GLY	198 PRO	191 PRO		
49 PRO	48 PRO	95 ASP	96 ASP	143 LEU	144 LEU	199 LEU	192 LEU		
50 ASN	49 ASN	98 PRO	97 PRO	144 LEU	145 LEU	200 VAL	193 VAL		
51 PHE	50 PHE	99 VAL	98 VAL	145 GLY	146 GLY	201 CYS	194 CYS		
52 VAL	51 VAL	99A ASN	99 ASN	147 ARG	147 ARG	204 ASN	195 ASN		
53 MET	52 MET	99B LEU	100 LEU	148 ASN	148 ASN	205 GLY	196 GLY		
54 SER	53 SER	100 LEU	101 LEU	149 ARG	149 ARG	208 LEU	197 LEU		
55 ALA	54 ALA	101 ASN	102 ASN	150 GLY	150 GLY	209 ILE	198 ILE		
56 ALA	55 ALA	102 ASP	103 ASP	151 ILE	151 ILE	210 HIS	199 HIS		
57 HIS	56 HIS	103 ILE	104 ILE	152 ALA	152 ALA	211 GLY	200 GLY		
58 CYS	57 CYS	104 VAL	105 VAL	153 SER	153 SER	212 ILE	201 ILE		
59 VAL	58 VAL	105 ILE	106 ILE	154 VAL	154 VAL	213 ALA	202 ALA		
60 ALA	59 ALA	106 LEU	107 LEU	155 LEU	155 LEU	214 SER	203 SER		
61 ASN	60 ASN	107 GLN	108 GLN	156 GLN	156 GLN	215 PHE	204 PHE		
62 VAL	61 VAL	108 LEU	109 LEU	157 GLU	157 GLU	216 VAL	205 VAL		
62A ASN	62 ASN	109 ASN	110 ASN	158 LEU	158 LEU	217A ARG	206 ARG		
62B VAL	63 VAL	110 GLY	111 GLY	159 ASN	159 ASN	218 GLY	207 GLY		

UV-Stability

The stability of the compounds was evaluated using UV-Visible spectrophotometry in a Thermo Scientific Evolution 201 UV-Visible spectrophotometer. In the case of type I qABPs, 3 *μ*l of a 15 mM stock solution in DMSO were diluted in 3 ml of PBS and the UV spectrum was measured over time until 48h, and in the case of type II a stock solution of the probes (15 mM) in DMSO was diluted in PBS/DMSO (final percentage of DMSO 20%) to a concentration of 1 *μ*M. All the compounds were also tested at pH 9, 10, 11, 12, and 13 until evidence of reaction was observed.

Gel-Based Assays

HEK-293 (CRL-1573, ATCC) and A431 (CRL-1555, ATCC) cells were grown in appropriate T75 culture flasks with DMEM medium supplemented with 10% FBS and 1% Penicillin/Streptomycin. U-937 (CRL-1593.2, ATCC) cells were grown in appropriate T25 culture flasks with RPMI medium supplemented with 10% FBS and 1% Penicillin/Streptomycin. After 48 h, cells were detached with trypsin, the volume of the cell suspension was made up to 10 ml with DMEM or RPMI, and the samples were centrifuged at 400 rcf. Human neutrophils were isolated from blood according to the procedure described in Kuang *et al*. [38] to obtain a stock (1.5 ml) of 4.5x106 cells/ml. The stock solution was centrifuged at 400 rcf. Pellets were dissolved in PBS and the lysis was performed with an ultrasonic homogenizer. The concentration of protein in whole cell lysates was calculated through a protein concentration assay (DCTM protein assay, BioRad), using as a reference Bovine Serum Albumin (BSA) and was normalized to 1 mg/ml or 0.6 mg/ml of protein. Regarding labelling experiments, 1 *μ*l of the tested probe (50x concentrated stock solution) or DMSO was incubated for 2 hours with 50 *μ*l of lysate at 37 °C. In the case of competitive assays, 1 *μ*l of ONO-6818 (50X concentrated stock solution) was added and incubated for 30 minutes with 50 *μ*l of lysate at 37 °C, followed by 1 *μ*l of probe (50x concentrated stock solution) for an additional 2 hours. The reaction was quenched by adding 4x gel loading buffer (17 *μ*l). Proteins were resolved by SDS-PAGE (15% acrylamide gel, using 40 V for 30 minutes, followed by approximately 1 hour at 100 V). In-gel fluorescence scanning was performed on an iBright™ FL1500 (TermoFisher Scientific) using as dyes Alexa Fluor 488 (excitation: 490 nm, emission: 525 nm), Cyanine3 (excitation: 554 nm, emission: 566 nm) and Cyanine5 (excitation: 647 nm, emission: 665 nm).

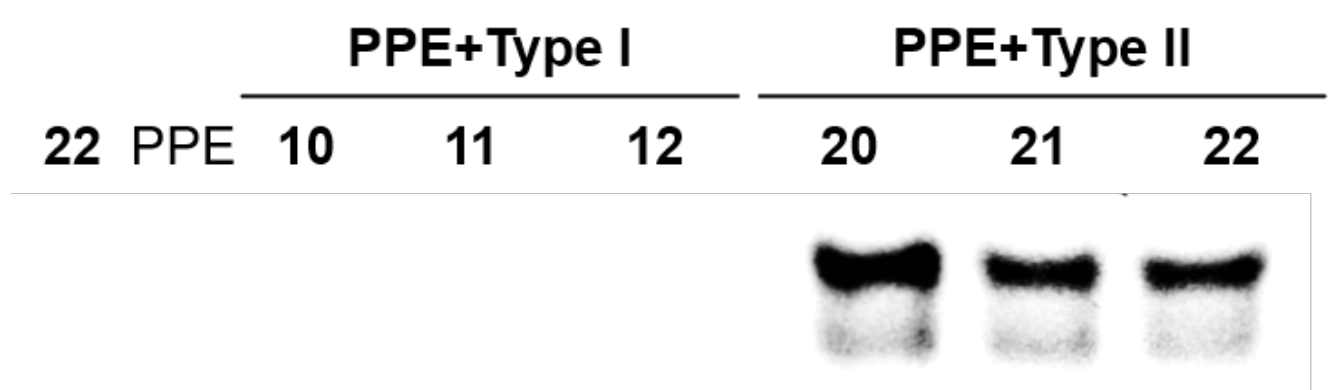

Figure S48. Incubation of the probes (1 μM) with PPE.

Limit of Detection Calculation

Fluorescence intensities were determined by densitometric analysis of the in-gel fluorescence bands shown in Figure 8 using the iBright Analysis Software (Thermo Fisher Scientific). Linear regression analysis was performed by plotting fluorescence intensity against HNE concentration. The limit of detection (LOD) was calculated according to the equation LOD = 3.3σ/slope, where σ is the standard deviation of the blank samples and the slope was obtained from the corresponding calibration curve.

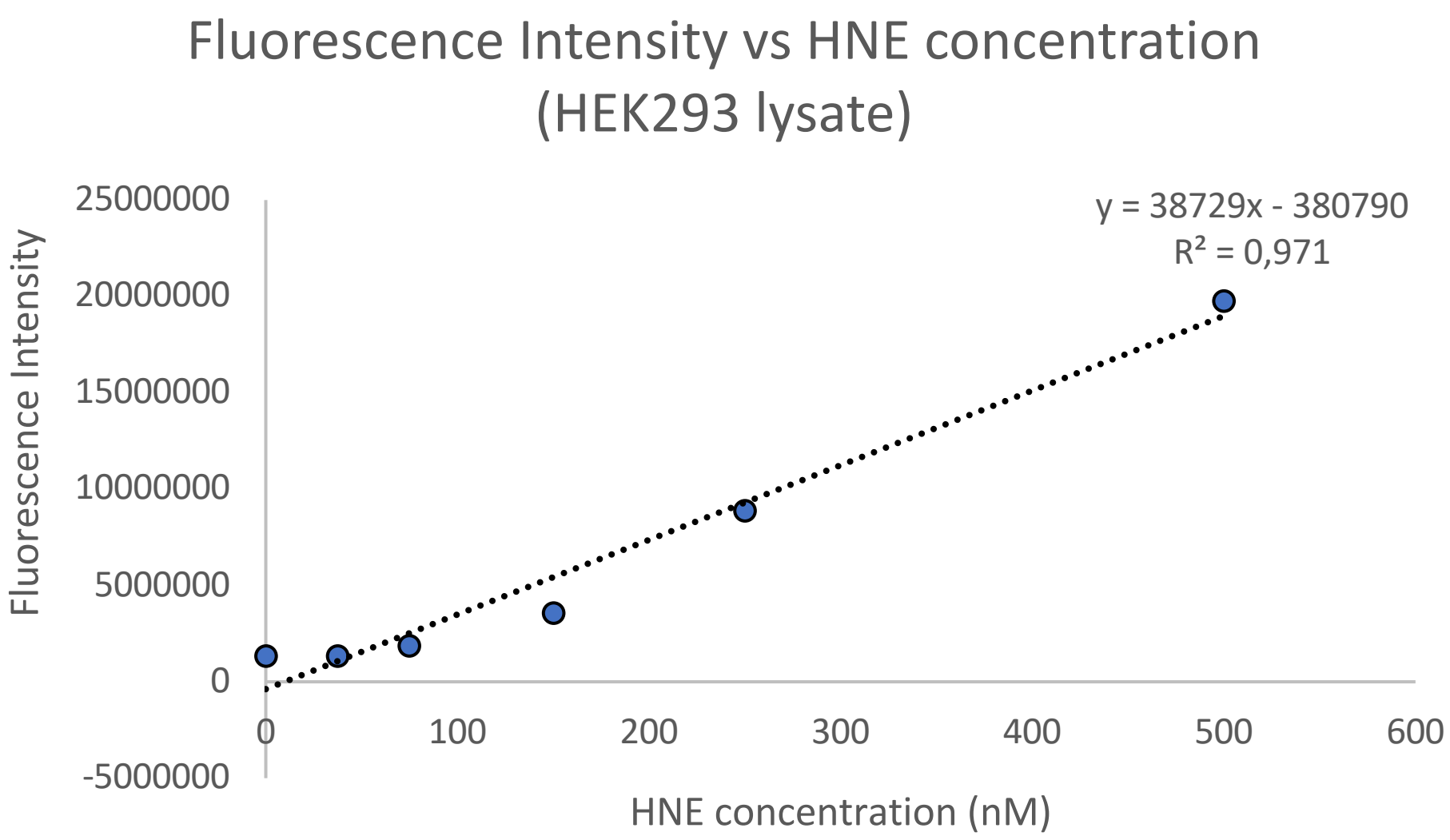

Figure S49. Correlation between HNE concentration and fluorescence signal intensity obtained with probe 22 in HEK cell lysate.

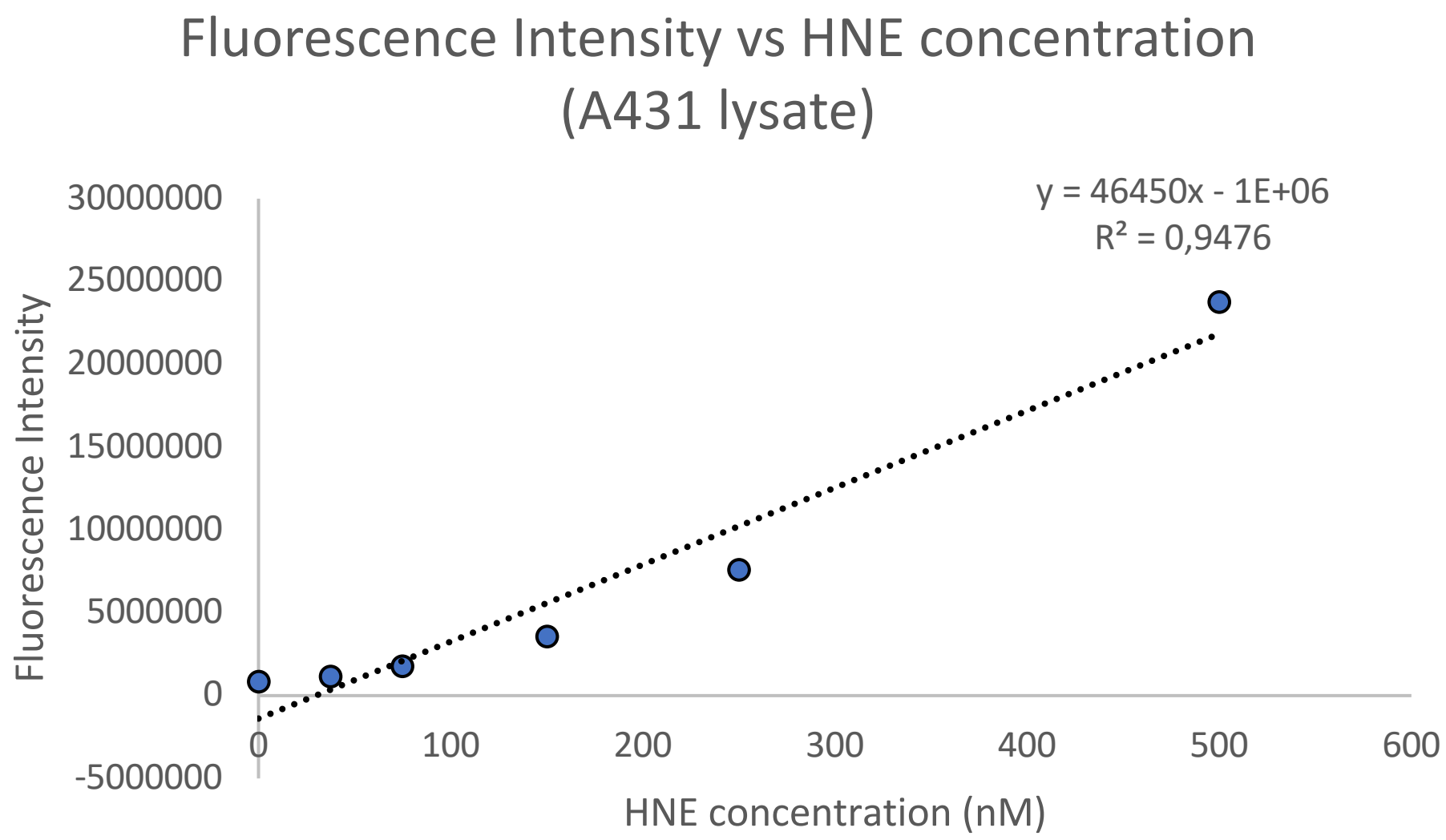

Figure S50. Correlation between HNE concentration and fluorescence signal intensity obtained with probe 22 in A431 cell lysate.

Fluorescence Studies

Fluorescence Scan

Fluorescence measurements were done on a SHIMADZU Spectro fluorophotometer RF-6000 instrument. Fluorescence was used to detect the activation with hydroxide, the fluorescence spectra of the samples used in UV-visible were measured before and after activation, obtained using as excitation wavelength the maximum absorption of the probe. The fluorescence quantum yield of the synthesized probes was calculated with an indirect method using Fluorescein as a reference (Φ_F=89% in 0.1M NaOH), as described in the literature. (39) Enzyme assays to observe the reaction with qABPs were performed in 100 mM HEPES buffer (pH 7.4) at 37 °C. A total of 15 μL purified enzyme (20 μM) was added to 3 ml of buffer containing probes (1.5 μM). The samples were excited at the maximum of emission of the probes, and the fluorescence intensity enhancement was monitored.

Fluorescence kinetics with HNE and neutrophils

Fluorescence with pure HNE and neutrophil lysates (prepared in gel-based assays) was performed in a 96-well plate and read in an Anthos multimode fluorometer Zenyth 3100, using as method Fluorescein Top (0.4s), 5 seconds shaking before each reading, and an excitation filter of 485 nm and emission filter

of 535 nm. Each well contained 10 μl of pure HNE (stock solution 4 μM) or 50 μl of neutrophils lysate, 1 μl of the probe (stock solution 50 μM) and HEPES buffer (HNE) or PBS (neutrophils lysate) up to 200 μl. The fluorescence was read in 2 minutes interval for 2 hours. The assays were performed in triplicate.

Biological activity

HNE inhibition activity was carried out, as described in the literature [36], in 200 μl assay buffer containing 50 μl of 80 nM HNE (Merck, Germany, from stock solution 2 μM in 0.05 M acetate buffer, pH 5.5) in assay buffer, 95 μl assay buffer and 5 μl of each concentration of tested inhibitors. After a period of 30 minutes of incubation at 25 °C the reaction was initiated by the addition of 50 μl of 4 mM chromogenic substrate (N-MeOSuc-Ala-Ala-Pro-Val-p-nitroanilide, Sigma, UK) in assay buffer, and activity was monitored at 410 nm for 60 min, at 25 ºC. Due to solubility issues for more concentrated solutions, 90 μl assay buffer were used instead of 95 μl while 5 μl of DMSO were to all solutions used for progress curve evaluation, activity was monitored at 410 nm at 25 °C for 120 min.

Selected compounds were evaluated for their ability to inhibit other human serine proteases, namely chymotrypsin, thrombin, kallikrein, urokinase, and Porcine Pancreatic Elastase (PPE), in 200 μL reaction volumes at 25 °C according to the previously published experimental methods. [36] Briefly, the analysis of chymotrypsin (Calbiochem) inhibition was performed in reaction mixtures containing 0.05 M Tris-HCl, 0.138 M NaCl, pH 8.0, 30 nM human pancreas chymotrypsin, test compounds, and 100 μM substrate (Suc-Ala-Ala-Pro-Phe-7-amino-4-methylcoumarin) (Bachem). The thrombin (Calbiochem) inhibition was evaluated in reaction mixtures containing 0.01 M sodium phosphate, 0.138 M NaCl, 0.1% PEG 6000, pH 7.0, 1.7 U/ml human plasma thrombin, test compounds, and 50 μM substrate (Z-Gly-Gly-Arg-AMC.HCl) (Bachem). The analysis of kallikrein (Calbiochem) inhibition was performed in reaction mixtures containing 0.05 M Tris-HCl, 0.138 M NaCl, pH 8.0, 2 nM human plasma kallikrein, test compounds, and 50 μM substrate (H-Pro-Phe-Arg-AMC acetate salt) (Bachem). The analysis of urokinase (Calbiochem) inhibition assay was performed in reaction mixtures containing 0.05 M Tris-HCl, 0.138 M NaCl, pH 8.0, 30 U/ml human urine urokinase, test compounds, and 50 μM substrate (Z-Gly-Gly-Arg-AMC.HCl). The analysis of PPE (Calbiochem) inhibition was performed in reaction mixtures containing 100mM HEPES buffer, pH 7.4, and 20 μL of 50 μM PPE, test

compounds, and 200 µM substrate (Z-Gly-(MeO-Suc-Ala-Ala-Pro-Val-AMC (Merck, Germany).

For all serine proteases activity was measured at excitation and emission wavelengths of 360 and 460 nm, respectively in a microplate reader (FLUOstar Omega, BMG Labtech, Germany). For all compounds tested, each concentration was tested in triplicate, and the concentration of inhibitor that caused 50% inhibition of the enzymatic reaction (IC_{50}) was determined by non-linear regression using GraphPad PRISM software as previously published. [36]

Probe Internalisation by Flow Cytometry

Probe internalisation was tested in U-937 cell line in MEM without red phenol, at 100,000 cells per well. Cells were incubated with qABP **22** at 5 µM per triplicate; cells without qABP, were used as negative control. After 2h, cells were analysed by flow cytometry (Accuri C6 Plus Cytometer, BD Biosciences, Eysins, Switzerland). Viable cells were gated based on forward scatter-area and side scatter-area characteristics. Histograms were generated by plotting the x-axis represents the fluorescence intensity measure in FL1 channel (with 530 nm filter excited by 488 nm laser), and the cell count is showed in the y-axis.

Live Cell Imaging

Probe internalisation was tested in human neutrophils, isolated from blood as it is described above, in Gel-based assay subsection. Frozen neutrophils were thawed and directly seeded in Corning® 384-well Black and Clear Bottom Microplates with 10.000 cells per well in neutrophils buffer (5 mM HEPES, 0.2%BSA in HBSS). Neutrophils were incubated with qABP **22** at 5 µM per triplicate; cells without qABP, were used as negative control. After 2h, live imaging of cells was acquired by Operetta CLS High-Content Analyse System (Revvity, Waltham, Massachusetts, United States) with brightfield and FITC (460-490 nm/500-550 nm excitation/emission) channels, with 63x water objective.

Molecular docking calculations

The three-dimensional structure of human neutrophil elastase was obtained from the Protein Data Bank (PDB ID: 9SI4[39]). Protein preparation was carried out in Molecular Operating Environment (MOE) 2024.0601 (Molecular Operating Environment (MOE), 2024.0601; Chemical Computing Group ULC: Montreal, QC, Canada, 2026, chemcomp.com) by adding missing side chains and hydrogen

atoms, assigning bond orders, removing solvent, and setting protonation states with Protonate3D at pH 7.4. The hydrogen-bonding network was optimized, and the structure was subjected to restrained energy minimization to relieve local strain while preserving the experimental backbone geometry. The prepared receptor was then imported into the Schrödinger suite (Schrödinger Release 2025-1: Maestro; Schrödinger, LLC: New York, NY, 2025) and further refined using the OPLS4 force field. [40]

Ligand structures were built in MOE 2024.0601, and protonation/tautomeric states were assigned at pH 7.4. Each ligand was geometry-optimized with OPLS4, and the resulting low-energy conformers were retained for docking.

Noncovalent docking was performed with Glide. [41] Prior to production calculations, the protocol was evaluated by self-docking (redocking of the co-crystallized ligand) and cross-docking using Smina, [42] GNINA 1.3, [43] and Glide to assess pose reproducibility and scoring consistency. Receptor grids were generated in Glide and centred on the binding site defined by the co-crystallized ligand. During grid generation and docking, a ligand van der Waals scaling factor of 0.80 and a partial charge cutoff of 0.15 were applied to soften nonpolar contacts. Docking was conducted in extra precision (XP) mode with ligand flexibility enabled. Up to 10 poses per ligand were generated, energy-minimized, and ranked by GlideScore.[41] The top-ranked poses were retained for subsequent interaction and structural analysis.

Covalent docking was performed using CovDock (Schrödinger[40]). Ser188 (or Ser195) was specified as the reactive residue, and the electrophilic warhead was defined to form a covalent adduct consistent with a *β*-lactamase-like ring-opening mechanism (*β*-lactam addition). The CovDock workflow comprised an initial noncovalent sampling stage, followed by covalent bond formation and local refinement of the adduct using OPLS4. Final poses were evaluated using the CovDock scoring function, and the 10 highest-scoring covalent complexes were selected for binding and structural assessment.
